# The brachyceran de novo gene PIP82, a phosphorylation target of aPKC, is essential for proper formation and maintenance of the rhabdomeric photoreceptor apical domain in *Drosophila*

**DOI:** 10.1371/journal.pgen.1008890

**Published:** 2020-06-24

**Authors:** Andrew C. Zelhof, Simpla Mahato, Xulong Liang, Jonathan Rylee, Emma Bergh, Lauren E. Feder, Matthew E. Larsen, Steven G. Britt, Markus Friedrich

**Affiliations:** 1 Department of Biology, Indiana University, Bloomington, Indiana, United States of America; 2 Department of Neurology and Ophthalmology, Dell Medical School, University of Texas, Austin, Texas, United States of America; 3 Department of Biological Sciences, Wayne State University, Detroit, Michigan, United States of America; New York University, UNITED STATES

## Abstract

The *Drosophila* apical photoreceptor membrane is defined by the presence of two distinct morphological regions, the microvilli-based rhabdomere and the stalk membrane. The subdivision of the apical membrane contributes to the geometrical positioning and the stereotypical morphology of the rhabdomeres in compound eyes with open rhabdoms and neural superposition. Here we describe the characterization of the photoreceptor specific protein PIP82. We found that PIP82’s subcellular localization demarcates the rhabdomeric portion of the apical membrane. We further demonstrate that PIP82 is a phosphorylation target of aPKC. PIP82 localization is modulated by phosphorylation, and *in vivo*, the loss of the aPKC/Crumbs complex results in an expansion of the PIP82 localization domain. The absence of PIP82 in photoreceptors leads to misshapped rhabdomeres as a result of misdirected cellular trafficking of rhabdomere proteins. Comparative analyses reveal that PIP82 originated de novo in the lineage leading to brachyceran Diptera, which is also characterized by the transition from fused to open rhabdoms. Taken together, these findings define a novel factor that delineates and maintains a specific apical membrane domain, and offers new insights into the functional organization and evolutionary history of the *Drosophila* retina.

## Author summary

Photoreceptors are the critical cells for detecting light. Changes in their morphology, organization and wiring in visual systems can greatly influence light sensitivity and visual acuity. Here we address the role of the protein PIP82 in regulating photoreceptor morphology and its potential role in the adaptive transformation from fused to open rhabdoms in insect photoreceptor organization. Our data indicate PIP82 is a downstream effector molecule of a conserved transcriptional pathway regulating photoreceptor differentiation. However, our phylogenetic analysis demonstrates that PIP82 is not present in all insects. PIP82 presence correlates with the appearance of open rhabdoms in brachyceran flies and the specialization of the photoreceptor apical domain into two distinct functional domains, the rhabdomere and stalk membrane. We find PIP82 only localizes to the rhabdomere apical domain. Moreover, the localization of PIP82 is regulated by aPKC dependent phosphorylation revealing a cellular mechanism to potentially delineate the boundary between the two apical domains of open rhabdoms. Loss of function analysis demonstrates PIP82 is necessary to generate and maintain rhabdomere morphology via the proper localization of proteins to the rhabdomere. Taken together our findings reveal a process in which a potential *de novo* protein intersects with known regulators of apical/basal polarity and cellular trafficking which facilitated the evolutionary transition from fused to open rhabdoms in early brachyceran flies.

## Introduction

Apposition compound eyes, the common visual structures found in many arthropods [1] consist of individual units known as ommatidia, which contain defined sets of photoreceptor cells. The photoreceptors are characterized by the formation of an apical phototransduction organelle, the rhabdomere, and axons that segregate into the lamina and medulla visual neuropils of the central nervous system. Changes in the size and organization of phototransduction organelles and/or the wiring of the photoreceptor axons permit adaptive fine tuning of light sensitivity and visual acuity [1–6]. *Drosophila* including other higher order Brachycera flies combine two adaptations, open rhabdoms and neural superposition, to increase light sensitivity without a commensurate loss of visual resolution [2, 4, 7, 8]. Rhabdoms, the collection of all rhabdomeres within a single ommatidium, exist in two different types of organizations among apposition compound eyes. The most frequent type is the fused rhabdom, in which all the rhabdomeres within a single ommatidium are juxtaposed to each other. The less frequent type, open rhabdoms, are characterized by a pronounced inter-rhabdomeral space (IRS), an extracellular matrix, that separates each individual photoreceptor rhabdomere from the others.

Neural superposition, which correlates with open rhabdom organization, refers to how photoreceptor axons project into the lamina cartridge of the retina neuropil. In this case, the axons of photoreceptor cells in adjacent ommatidium that “see” the same point in space converge onto the same lamina cartridge in the lamina neuropil [2, 4]. In insect eyes with fused rhabdoms, by contrast, the photoreceptor axons project from a single ommatidium to converge on a single lamina cartridge. Of note, the presence of both adaptations is required for a functional visual system. Moreover, it is the correlated evolution of open rhabdoms and neural superposition which is thought to have permitted some diurnal dipterans to diversify into niches characterized by low light [8].

How neural superposition and open rhabdom organization emerged at the cellular and molecular levels remain incompletely understood. Foundational work on axon pathfinding in *Drosophila* has established the guiding principles that permit the sorting of photoreceptor axons in the retina neuropil [9–11]. However, how these principles are different or the molecules that generate the differences in wiring in open versus fused visual systems remains unknown (reviewed in [7]). With respect to open rhabdom organization, genetic analysis in *Drosophila* has identified two critical proteins, Eyes Shut (EYS also referred to as Spacemaker) and Prominin, that are components of a cellular network that generates the IRS involving the secretion of an extracellular matrix, steric hindrance of adhesion and cellular contraction [12–16]. Functional comparative analyses of Prominin and EYS in different insects with open or fused rhabdoms demonstrated at least three molecular changes that occurred during the transition from fused to open in the ancestral lineage to brachyceran Diptera. Accordingly, this included changes in the cis-regulatory network regulating EYS expression, changes in EYS protein sequence permitting the transfer of EYS to the apical surface and the origination of a novel interaction between EYS and Prominin [17].

Despite this progress much remains to be learned how the development of open rhabdoms compares to that of fused rhabdoms (reviewed in [7]). Here we evaluate an orphan protein, PIP82, which was nominally mentioned as a potential component or downstream effector molecule of phototransduction [18]. Our studies indicate PIP82 is a downstream effector molecule of the conserved Glass/Pph13 transcriptional pathway required for rhabdomeric photoreceptor differentiation among Pancrustaceans [19–21]. Nonetheless, the gene is only present in a select group of insects. Phylogenetic analysis indicates that PIP82 is limited to brachyceran Diptera. PIP82 presence thus corelates with the appearance of open rhabdoms in this clade and the specialization of the photoreceptor apical domain into two distinct functional domains, the rhabdomere and stalk membrane. Functionally, we demonstrate that PIP82 demarcates the rhabdomeric portion of the photoreceptor apical membrane. Moreover, PIP82 is a direct phosphorylation target of aPKC, thus generating a molecular link between the role of Crumbs in maintaining the stalk membrane [22, 23] and highlighting a potential mechanism for delineating a boundary between the two apical domains. Our mutational analysis reveals that PIP82 is required for the establishment and maintenance of rhabdomere shape and integrity due to the inability to traffic or retain rhabdomeric proteins. Taken together, our findings reveal a process in which a *de novo* protein intersects with known regulators of apical/basal polarity and cellular trafficking to potentially strengthen the evolutionary transition from fused to open rhabdoms in early brachyceran flies.

## Results

### PIP82 is a photoreceptor specific terminal differentiation gene

PIP82 was first noted as a potential light dependent phospho-regulated protein [18]. Subsequently, we identified PIP82 in two different transcriptome studies designed to identify genes required for the differentiation of *Drosophila* photoreceptors [24, 25]. In particular, our transcriptome analyses suggested that PIP82 is part of the Glass-Pph13 dependent transcriptional cascade that is essential for photoreceptor cell development [19, 25, 26]. Recent studies revealed that Glass has two temporal roles with respect to retina development, specification and differentiation [19, 25, 26]. With respect to differentiation, the function of Glass is aided by the Glass mediated transcription of the homeodomain containing transcription factor Pph13 [25, 27, 28]. To elucidate the indicated role of PIP82 in retina development as well as to decipher the potential transcriptional input of both Glass and Pph13 in regulating PIP82 expression, we generated a transcriptional reporter for and antibody against PIP82.

**Fig 1:**
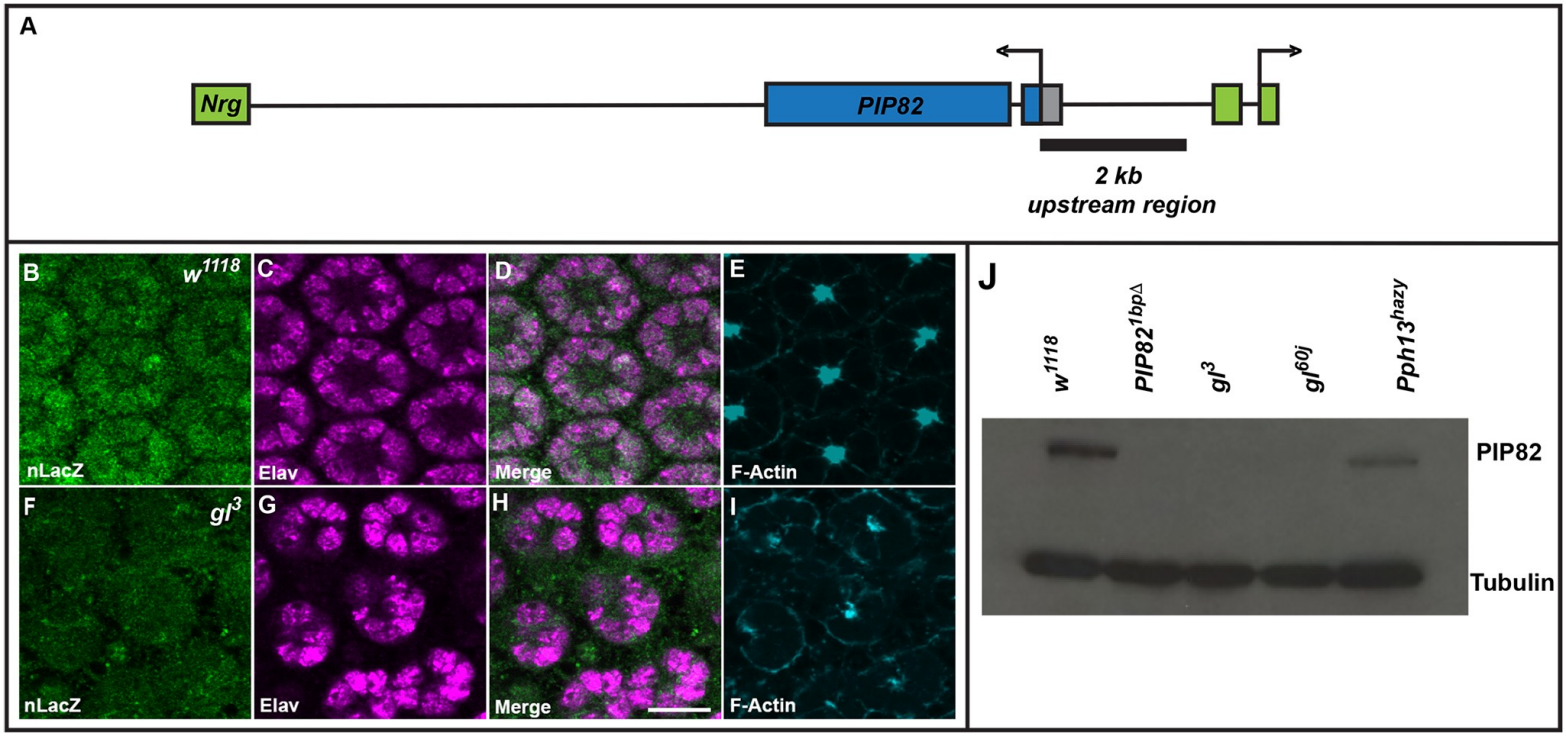
PIP82 is a photoreceptor specific terminal differentiation gene. A. Schematic of *PIP82* locus with respect to *neuroglian* and location of the 2kb region utilized in the *PIP82* transcriptional reporter. B-E. Immunofluorescence staining of wild type photoreceptors imaged for the *PIP82* transcriptional reporter, nLacZ,(B), Elav (C), F-Actin (E). F-I. Immunofluorescence staining of *glass* mutant photoreceptors imaged for the *PIP82* transcriptional reporter, nLacZ,(F), Elav (G), F-Actin (I). Panels D and H represent merge images of B/C and F/G respectively. Each image is a single confocal section and retinas were examined at 48 hours after puparium formation. Scale bar is 10uM. J. Western analysis of PIP82 and α-Tubulin expression in wild type (*w*^*1118*^), *PIP82* mutant (*PIP82*^*1bpΔ*^), *glass* mutants (*gl*^*3*^ and *gl*^*60j*^), and *Pph13* mutant (*Pph13*^*hazy*^) whole head extracts.

The *PIP82* locus resides on the X chromosome in the first intron of the gene *neuroglian* (*nrg*) and is transcribed in the opposite direction of *nrg* (www.Flybase.org). An approximate 2kb upstream region extending from the first coding methionine of PIP82 was utilized to drive expression of nLacZ and reproduced the endogenous expression (Fig 1A). Both reporter gene expression and immunofluorescence detection of PIP82 revealed PIP82 is only expressed in photoreceptors and not detected in any other tissue, in agreement with modENCODE data [29]. Notably, PIP82 expression was not detected in photoreceptors of the 3^rd^ instar eye imaginal disc upon the initiation of Glass expression. Rather, expression was first observed approximately 48 hrs after puparium formation (APF) and maintained post-eclosion only in photoreceptors (Fig 1B–1E), suggesting PIP82 does not have a role in Glass mediated retina specification. Notwithstanding this finding, we further found that PIP82 expression was eliminated upon the removal of Glass (Fig 1F–1I and 1J) and that the loss of Pph13 resulted in lower levels of PIP82 (Fig 1J) in agreement with transcriptome data from *glass* and *Pph13* mutants [24, 25]. Together these analyses suggested that PIP82 has a role in photoreceptor terminal differentiation and that its transcriptional expression is driven by a Glass-Pph13 feed forward loop [30]. Specifically, Glass appears to be required for the activation of both PIP82 and Pph13, while Pph13 promotes the maximal expression of PIP82.

### PIP82 is a phosphorylation target of aPKC and localizes to the rhabdomeric membrane

**Fig 2:**
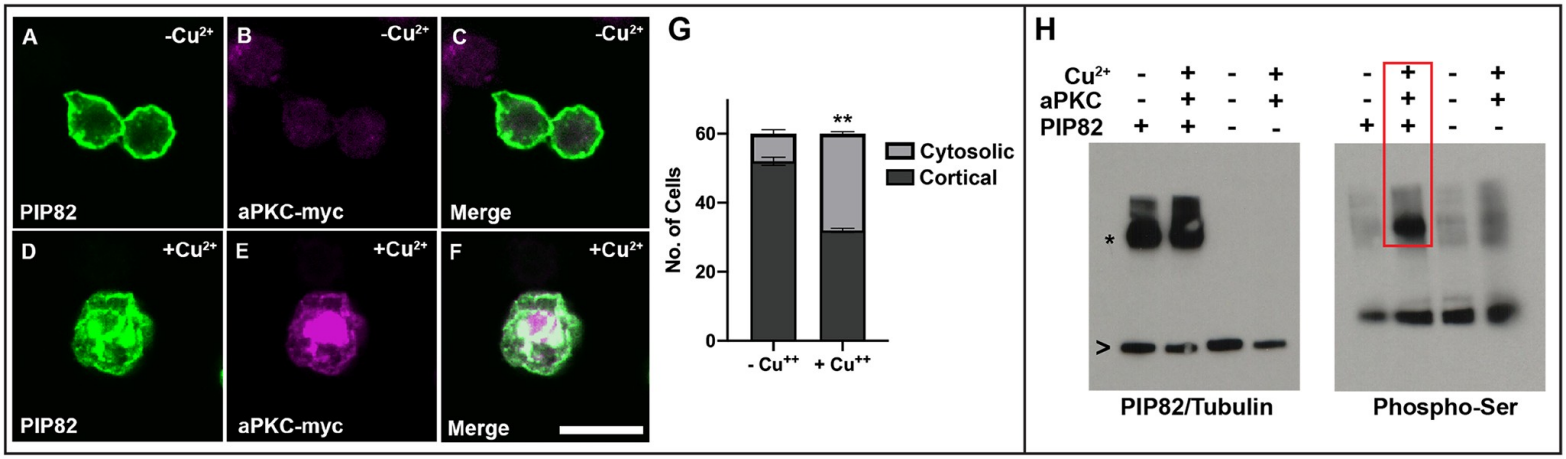
PIP82 is a cortical membrane protein and phosphorylation target of aPKC. Expression of PIP82 in *Drosophila* S2 cells in the absence (A-C) or presence of aPKC (D-F). PIP82 is in green (A,D) and aPKC in magenta and aPKC expression was induced in the presence of Cu^2+^. Each image is a single confocal section. Scale bar is 10uM. G. Quantification of the number of cells demonstrating the localization pattern of PIP82 in the absence and presence of aPKC. Cortical localization is representative of the pattern seen in panel A and cytosolic is representative of the pattern seen in panel B. ** p = 0.0025, paired t-test, n = 3. H. Western analysis of whole cell extracts, a sample of each extract was utilized for each blot, and probed for either PIP82 (asterisk) and α-Tubulin (carrot) or Phospho-Serine. The red box marks the PIP82/aPKC dependent phosphorylation event.

PIP82 is an *1195* amino acid protein with a candidate phospho-regulated basic and hydrophobic (PRBH) domain as its only identifiable signature structure (S1 Fig). PRBH domains directly interact with phospholipids permitting cortical localization. Phosphorylation of the PRBH domain by atypical Protein Kinase C (aPKC) inhibits the PRBH domain interaction with the membrane [31]. Previous results demonstrated that a PIP82 PRBH domain:GFP fusion protein showed enriched cortical localization in *Drosophila* tissue culture cells. This localization was mediated by the presence of aPKC, and the PRBH domain was phosphorylated by aPKC in an *in vitro* kinase assay [31]. To extend these results to the full-length PIP82 protein and to confirm this potential interaction *in vivo*, we first transfected *Drosophila* S2 cells with an expression plasmid for the full-length PIP82 protein; PIP82 is not expressed in native S2 cells [29]. Immunofluorescence detection upon transfection demonstrated that PIP82 localizes to the entire cortical surface (Fig 2A–2C). Upon the co-transfection and induction of aPKC, PIP82 is more likely to be displaced from the cortical membrane and found in the cytosol (Fig 2D–2G and S2 Fig). Consistent with this observation, a PIP82-aPKC dependent phosphorylation event could be detected in cellular extracts (Fig 2H).

**Fig 3:**
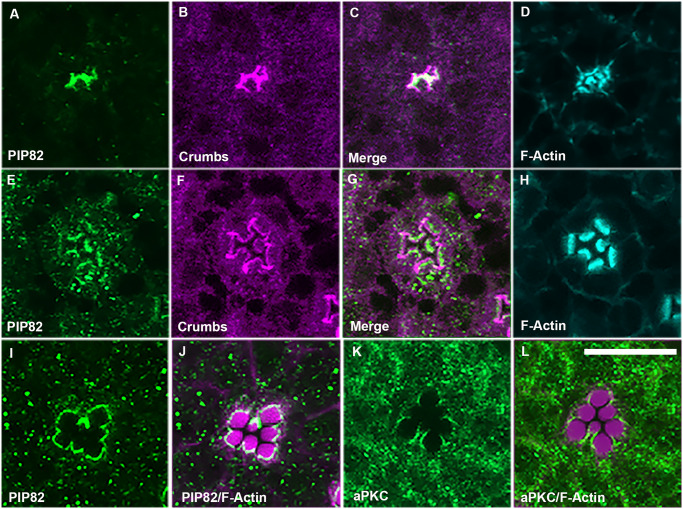
PIP82 and Crumbs/aPKC resolve and demarcate the two apical domains during photoreceptor differentiation. A-D. Immunostaining of PIP82 (green), Crumbs (magenta), and F-Actin (cyan) in wild type photoreceptors at 48 hrs APF. E-H. Immunostaining of PIP82 (green), Crumbs (magenta), and F-Actin (cyan) in wild type photoreceptors at 72 hrs APF. I,J. Immunostaining of PIP82 (green) and F-Actin (magenta) in wild type photoreceptors at 1 day post eclosion. K,L. Immunostaining of aPKC (green) and F-Actin (magenta) in wild type photoreceptors at 1 day post eclosion. Each image is a single confocal section. Scale bar is 10uM.

**Fig 4:**
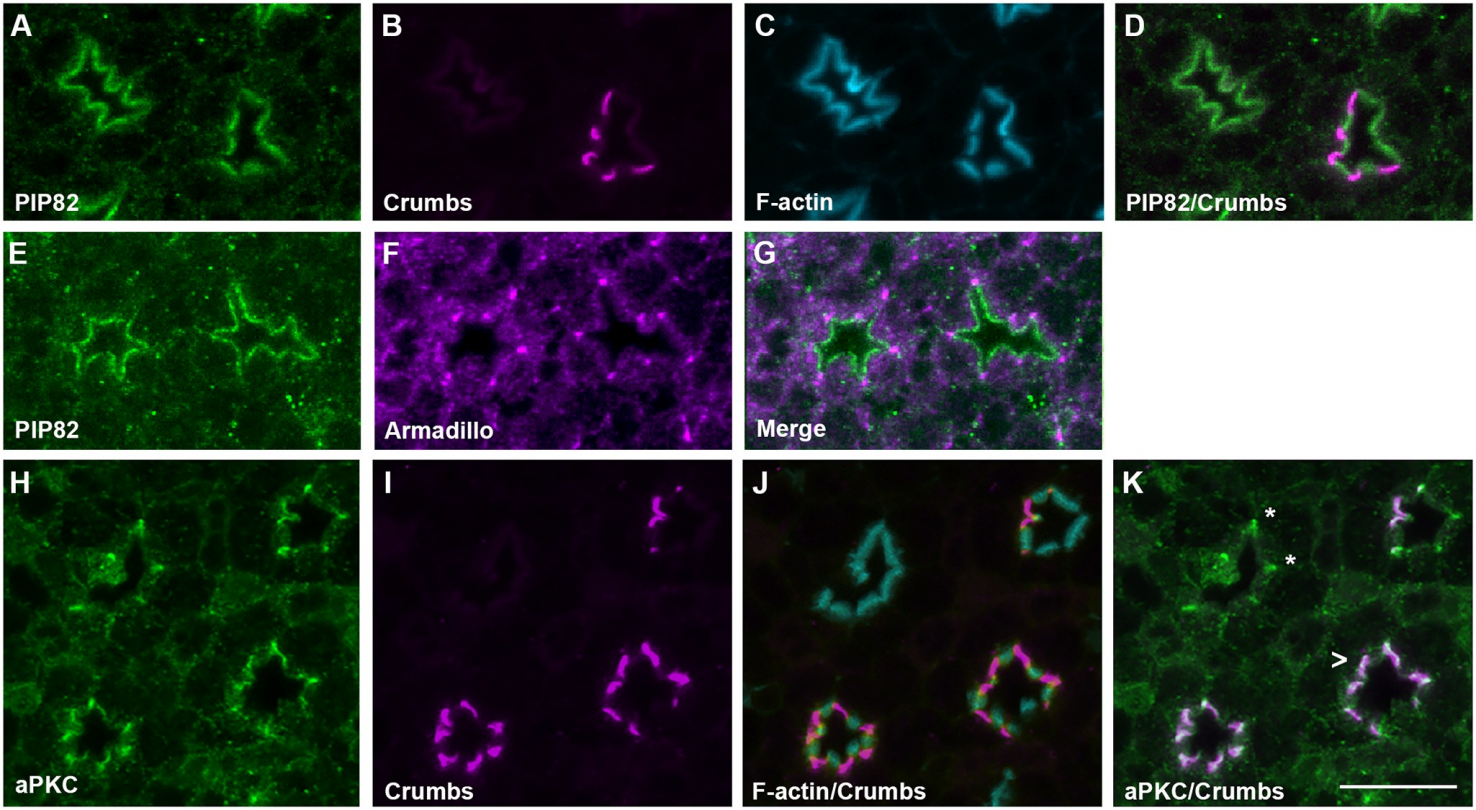
PIP82 localizes to the entire apical surface upon the loss of the Crumbs/aPKC complex. A-D. Immunostaining of PIP82 (green), Crumbs (magenta), and F-Actin (cyan) in mosaic clones containing both wildtype and mutant *crumb* photoreceptors at 84 hrs APF. Wild type photoreceptors are indicated by the presence of Crumbs staining. Note in the merge image (D), in the presence of Crumbs PIP82 is limited to only the rhabdomeric portion of the apical surface. E-G. Immunostaining of PIP82 (green), and Armadillo (magenta), in *crumb* mutant photoreceptors as noted by the localization pattern of PIP82 at 84 hrs APF. H-J. Immunostaining of aPKC (green), Crumbs (magenta), and F-Actin (cyan) in mosaic clones containing both wildtype and mutant *crumb* photoreceptors at 84 hrs APF. Wild type photoreceptors are indicated by the presence of Crumbs staining. Note in merge image (K), aPKC localizes on the putative stalk membrane only in the presence of Crumbs (carrot) and in the absence of Crumbs to the adherens junctions (asterisk). Scale bar is 10uM.

In photoreceptors, aPKC is critical for establishing apical/basal polarity. Moreover, the absence of aPKC results in photoreceptor cell death prior to differentiation [32]. The initial apical identity of photoreceptors is established by Cdc42 with the subsequent recruitment of Bazooka and the PAR6/aPKC complex [33]. In addition, the retention of aPKC by the transmembrane protein Crumbs at the apical surface reinforces the asymmetry between the apical and lateral membranes [34–39]. Moreover, Crumbs is also critical for defining and the morphogenesis of the apical stalk membrane of photoreceptors. Notably, while the levels of Crumbs can directly modulate the size of the stalk membrane, the complete loss of Crumbs does not eliminate the appearance of the stalk membrane but results in a greatly reduced stalk membrane and defects in rhabdomere shape and length [22, 23, 40]. Based on our cell culture data, we expected aPKC and PIP82 to engage in an antagonistic relationship; specifically, we hypothesized that the presence of aPKC would eliminate PIP82 from the cortical membrane. As a corollary of this, we further expected to observe two distinct non-overlapping domains of PIP82 and Crumbs/aPKC develop as the photoreceptor apical membrane is subsequently divided into two compartments. At approximately 48 hrs after puparium formation (APF), when the entire photoreceptor apical domain contains loosely associated microvilli projections [41, 42], we observed a spatial overlap of PIP82 and Crumbs localization by antibody staining (Fig 3A–3D). However, at 72 hrs APF, when two distinct apical domains are distinguishable, the intersection between PIP82 and Crumbs was greatly diminished; PIP82 localized to the base of the rhabdomere while Crumbs was concentrated to the stalk membrane with some Crumbs residual staining in the rhabdomere (Fig 3E–3H); PIP82 colocalization with Rhodopsin 1 (Rh1) at the base of the rhabdomere suggested PIP82 is not in the microvilli but rather found in the region of the rhabdomere terminal web [43] (S7 Fig). Moreover, utilizing an antibody that recognizes aPKC [33], we observed a non-overlapping spatial pattern of the PIP82 and aPKC domains at one day post eclosion (Fig 3I–3L). In this case, PIP82 demarcates the base of the rhabdomere and aPKC the stalk membrane of each photoreceptor. To further explore the hypothesis that the Crumbs/aPKC complex potentially regulates the localization of PIP82, we examined PIP82 localization in *crumbs* mutant clones at ~ 84 hrs after puparium formation. If Crumbs was present, PIP82 localization was limited to the rhabdomeric apical membrane and excluded from the putative stalk membrane of the photoreceptor (Fig 4A–4D). However, in the absence of Crumbs, PIP82 localization extended along the entire apical surface (Fig 4A–4D) from adherence junction to adherence junction as visualized with the adherence junction marker protein β-Catenin, Armadillo (Fig 4E–4G). Moreover, the localization of PIP82 along the entire apical surface correlated with the loss of aPKC on the apical surface; in the absence of Crumbs, aPKC was detected at the putative adherens junctions (Fig 4H–4J). Altogether, these results are consistent with the idea that PIP82 is a target of aPKC function and the phosphorylation of PIP82 delineates the boundaries of PIP82 localization. Furthermore, our data suggests that phenotypes associated with *crumbs* mutant photoreceptors, i.e. shortened stalk membranes and irregular shaped rhabdomeres, may be a direct result of aPKC participating in the morphogenesis of the apical photoreceptor membrane via the regulation of PRBH domain containing proteins as exemplified by the localization of PIP82 to a specific region of the apical surface.

### PIP82 is required for the development and maintenance of rhabdomere morphology

**Fig 5:**
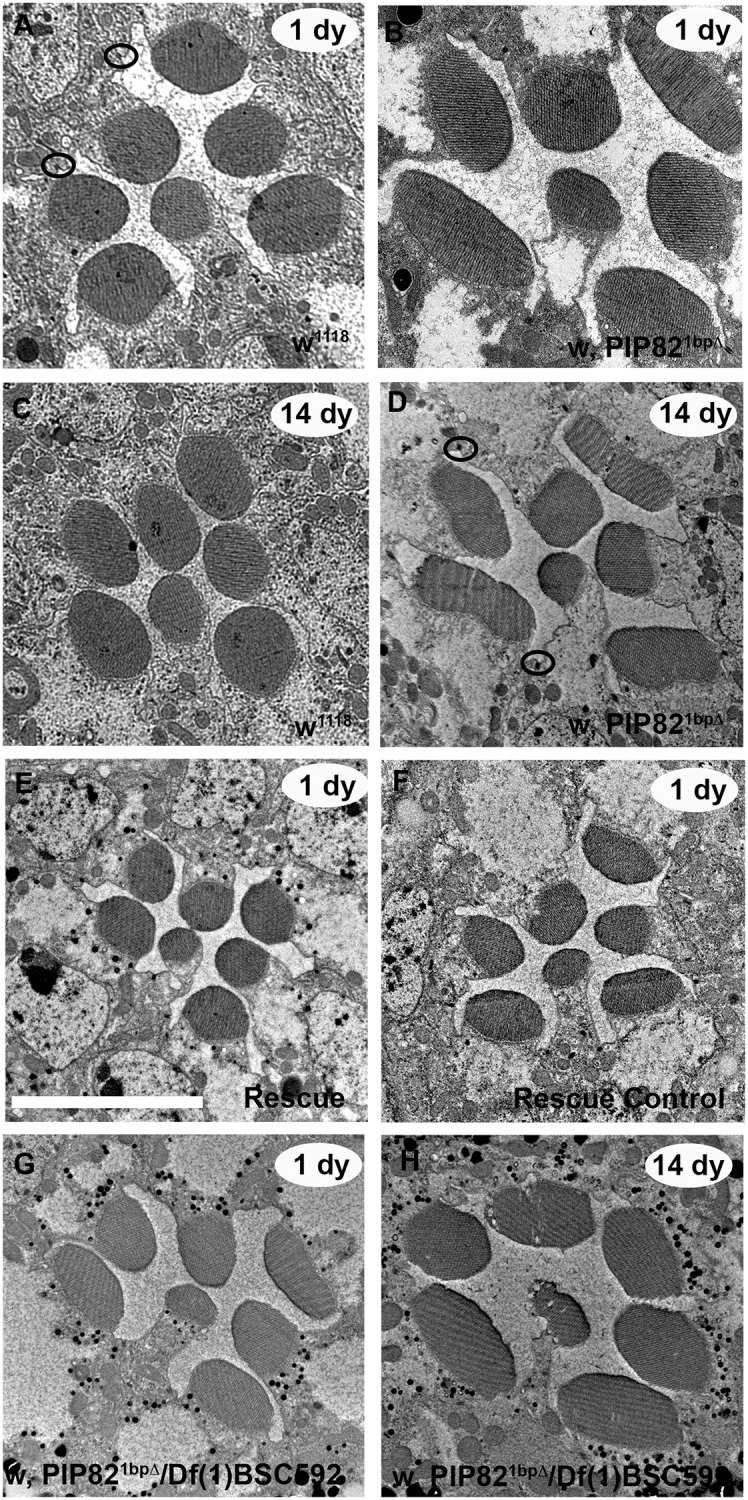
Transmission Electron Microscopy analysis of *PIP82* mutant photoreceptors. A,B. Rhabdomere structure in a single ommatidium of wild type (A) and *PIP82* mutant (B) one day (1 dy) post eclosion. C,D. Rhabdomere structure in a single ommatidium of wild type (A) and *PIP82* mutant (B) fourteen days (14 dy) post eclosion. E,F. Rescue (D) of *PIP82* mutant phenotype as compared to control is a single ommatidium. The rescue phenotype is *w*, *PIP82*^*1bpΔ*^/Y;+/+; UAS-PIP82/Pph13-GAL4 and the control phenotype is *w*, *PIP82*^1bpΔ^/ *w*, *PIP82*^*1bpΔ*^;+/+; UAS-PIP82/UAS-PIP82. G,H. Rhabdomere structure in a single ommatidium of 1 day and 14 day *PIP82* mutant over a deletion that uncovers the *PIP82* locus, *w*, *PIP82*^*1bpΔ*^*/Df(1)BSC592*. Scale bar is 5uM. Black circles in A and D highlight the adheren junctions that separate the stalk membranes of adjacent photoreceptors.

To investigate the role of PIP82 in rhabdomere morphogenesis, we utilized CRISPR/Cas9 to generate a mutant allele. The targeting of the second exon resulted in a single base pair deletion that would result in a frame shift, truncating the protein to only 107 aa extended by additional 38 unrelated amino acids. Importantly, if this truncated mutant protein is produced, it would lack the PRBH domain and western analysis confirmed the absence of the full-length native protein (S1 Fig and Fig 1J). As expected for the lack of function phenotype of a photoreceptor specific protein, the *PIP82* mutant is homozygous viable. TEM analysis of mutant retinas demonstrated that all photoreceptors were present and differentiated. Moreover, the photoreceptor apical membrane still developed two unique domains in the absence of functional PIP82. However, whereas wildtype rhabdomeres are circular in nature, the loss of functional PIP82 resulted in the formation of flat oblong rhabdomeres (Fig 5A and 5B). To determine whether the loss of PIP82 resulted in defects in the subdivision of the apical membrane we measured the apical domains of photoreceptors R3, R5, and R7 from wild type and *PIP82* mutant photoreceptors (S3 Fig). We observed stalk membrane lengths of R3 and R5 decreased by approximately 31% in *PIP82* mutants (p = 0.0007, Tukey’s HSD). Rhabdomere base lengths of R3 and R5 decreased by approximately 28% (p = 0.006, Tukey’s HSD). The stalk membrane length of R7 decrease by a relatively modest 13% in *PIP82* mutants (p = 0.024, Tukey’s HSD), while rhabdomere base length decreased by almost 30% on average (p = 0.043, Tukey’s HSD). These differences indicated that both the stalk membrane and rhabdomere decrease in size in *PIP82* mutants. This is also indicated by a lack of significance when comparisons are made using ratios of stalk to rhabdomere (p = 0.998), or rhabdomere to total measured membrane (p = 0.942). Moreover, given PIP82 localizes to the base of the rhabdomere with the region of the rhabdomere terminal web, we did not detect any structural abnormalities in this region, the stalk membrane of adherens junctions in mutant photoreceptors (S4 Fig).

The misshapen rhabdomeres were detected in both the homozygous mutant or heterozygous combination with a deficiency that removes the entire *PIP82* locus (Fig 5G and 5H). This is consistent with *PIP82*^*1bpΔ*^ allele being an amorphic loss of function allele. The mutant phenotype of oblong, flat rhabdomeres and loss of the close association of the microvilli becomes increasingly severe over the course of 14 days under 12 hr light / 12 hr dark conditions (Fig 5C and 5D). The phenotype of *PIP82*^*1bpΔ*^ homozygous mutants was rescued by the expression of a PIP82 transgene in mutant photoreceptors (Fig 5E and 5F). Lastly, the serial reconstructions utilizing serial block face SEM highlighted the irregularity and change of rhabdomere structure over time along the entire length of photoreceptor axis (S1–S3 Movies). By seven days post-eclosion, the *PIP82* mutant rhabdomeres have devolved from a coherent misshapen rod into an irregular misaligned fragmented structure. The alignment or organization of the microvillar projections along the depth of the retina are not maintained resulting in the appearance of bulges and complete irregularity of shape, suggesting that PIP82 is critical for influencing and maintaining the overall shape of the rhabdomeres as the photoreceptors age.

**Fig 6:**
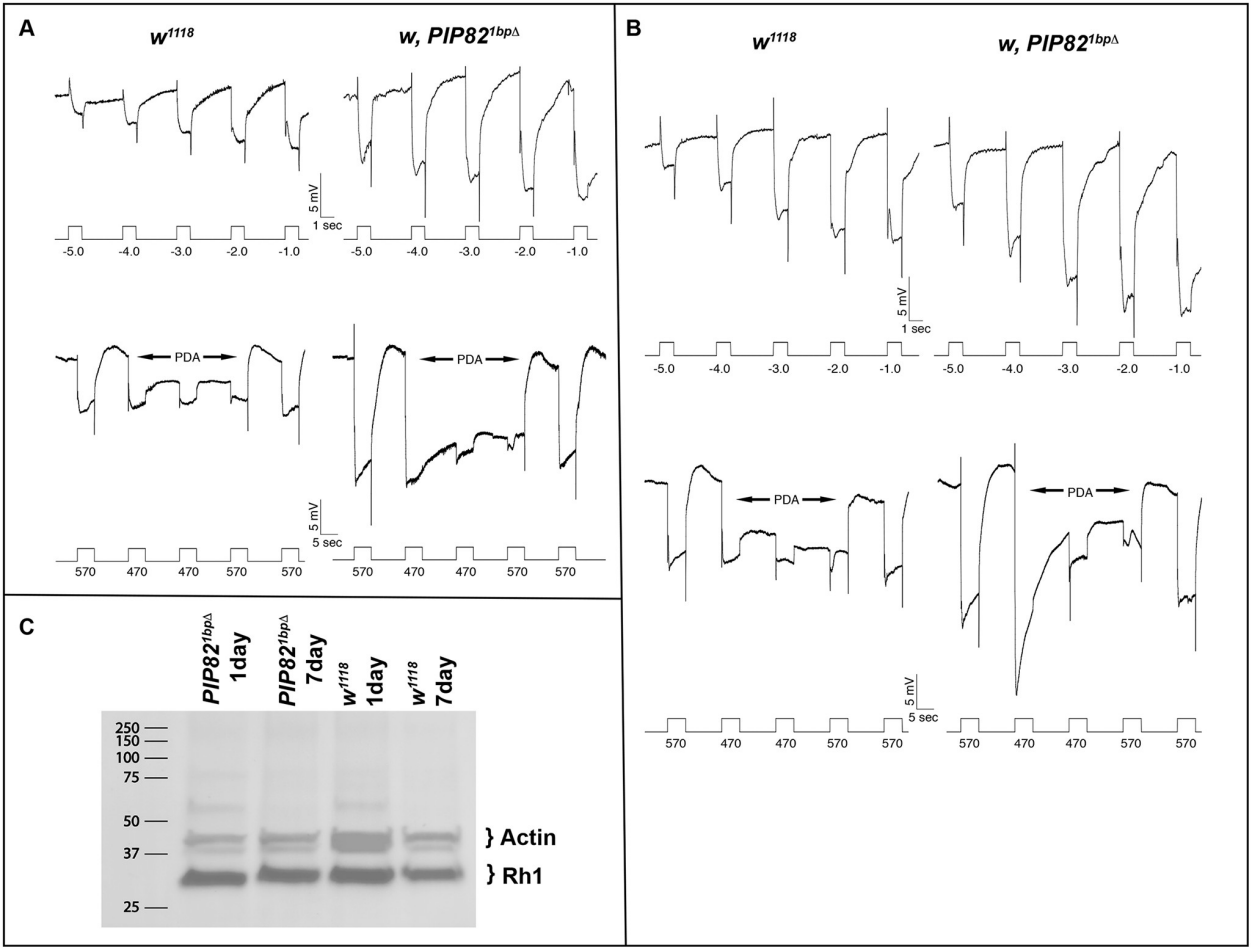
Electroretinogram and Rh1 analysis of wild type and *PIP82* mutant photoreceptors. Electroretinogram recordings from 1-day old (A) and 7-day-old (B) wild-type controls *w*^*1118*^, left column in both panels and w, *PIP82*^*1bpΔ*^ mutants, right column in both panels. Two stimulus paradigms were used to evaluate the light response of the animals. In the top row of both panels (A, B) flies were stimulated with increasing intensities of light (from -5.0 log attenuation to -1.0 log attenuation) at 470 nm. In the second row of both panels, flies were stimulated with full intensity light at the wavelengths indicated. In both control and mutant animals, a prolonged-depolarizing afterpotential (PDA) is induced. Full intensity (unattenuated) light was 1.6 mW/cm^2^ at 470 nm and 2.0 mW/cm^2^ at 570 nm. C. Western analysis of Rh1 levels as compared to Actin in *w*, *PIP82*^*1bpΔ*^ mutants and *w*^*1118*^ controls.

Numerous processes are known to contribute to rhabdomere integrity: phototransduction, cellular trafficking, and apical/basal polarity formation (reviewed in [38, 44, 45]). To define the mechanism leading to the loss of rhabdomere integrity in *PIP82* mutant photoreceptors we probed for all three underlying processes. To evaluate visual system function in *PIP82* mutants and to reveal a possible role in phototransduction as previously hypothesized [18], we recorded electroretinograms from 1- and 7-day old animals (Fig 6A and 6B) and compared them to *w* controls (*w*^*1118*^). At both ages, *w* controls and *PIP82* mutants responded to increasing intensities of light at 470 nm with depolarizations of increasing amplitude, as well as on- and off-transients at the beginning and end of the stimulus, respectively. In addition, we used a stimulus paradigm to test for a prolonged-depolarizing afterpotential (PDA). A PDA was induced when the fly was stimulated with bright light at 470 nm (thus producing a large amount of activated M-form of rhodopsin). The depolarization was maintained after the cessation of the stimulus, and the photoreceptor cells were relatively inactivated to further stimuli. When the fly was again stimulated with 570 nm light, the PDA was terminated by the photoconversion of the M-form back to the R-form. *w* control animals displayed a robust PDA. *PIP82* mutant animals also responded with a normal PDA. In both mutant and control genotypes, ERG response amplitudes appeared increased at 7 days compared to 1-day old animals. In all stimulus paradigms, the amplitude of the response appeared greater in the *PIP82* mutant animals compared to control. Quantitative analyses of ERG responses were performed on 7-day old *w* control and *PIP82* mutant animals, using the stimulus paradigm shown in Fig 6B. The response amplitudes at the two highest light intensities (-2 and -1) were significantly larger (47% and 65%, respectively) in *PIP82* mutant animals compared to w control animals (S5 Fig). These findings demonstrated that photoactivation of the visual pigment Rh1 (*ninaE*) and phototransduction occur in *PIP82* mutants and that the amplitudes of light response is enhanced at higher light intensities. Therefore, an absence of phototransduction is unlikely to contribute to the abnormal rhabdomere morphology in *PIP82* mutant photoreceptors.

With respect to the overall apical/basal polarity of the *PIP82* mutant photoreceptors, our TEM analyses did not reveal any obvious defects. All photoreceptors contained adherence junctions separating the apical and basal/lateral surfaces and the photoreceptors generated two distinct apical domains (Fig 5 and S4 Fig). Nonetheless, given the antagonistic relationship between PIP82 and aPKC, we tested whether aPKC localization was disrupted in *PIP82* mutants. Examination of mutant photoreceptors upon eclosion did not reveal any difference in aPKC localization (S6 Fig). This suggests that aPKC localization to the stalk membrane was independent of PIP82 and that PIP82 is not required to impede any spreading of aPKC into the rhabdomere.

**Fig 7:**
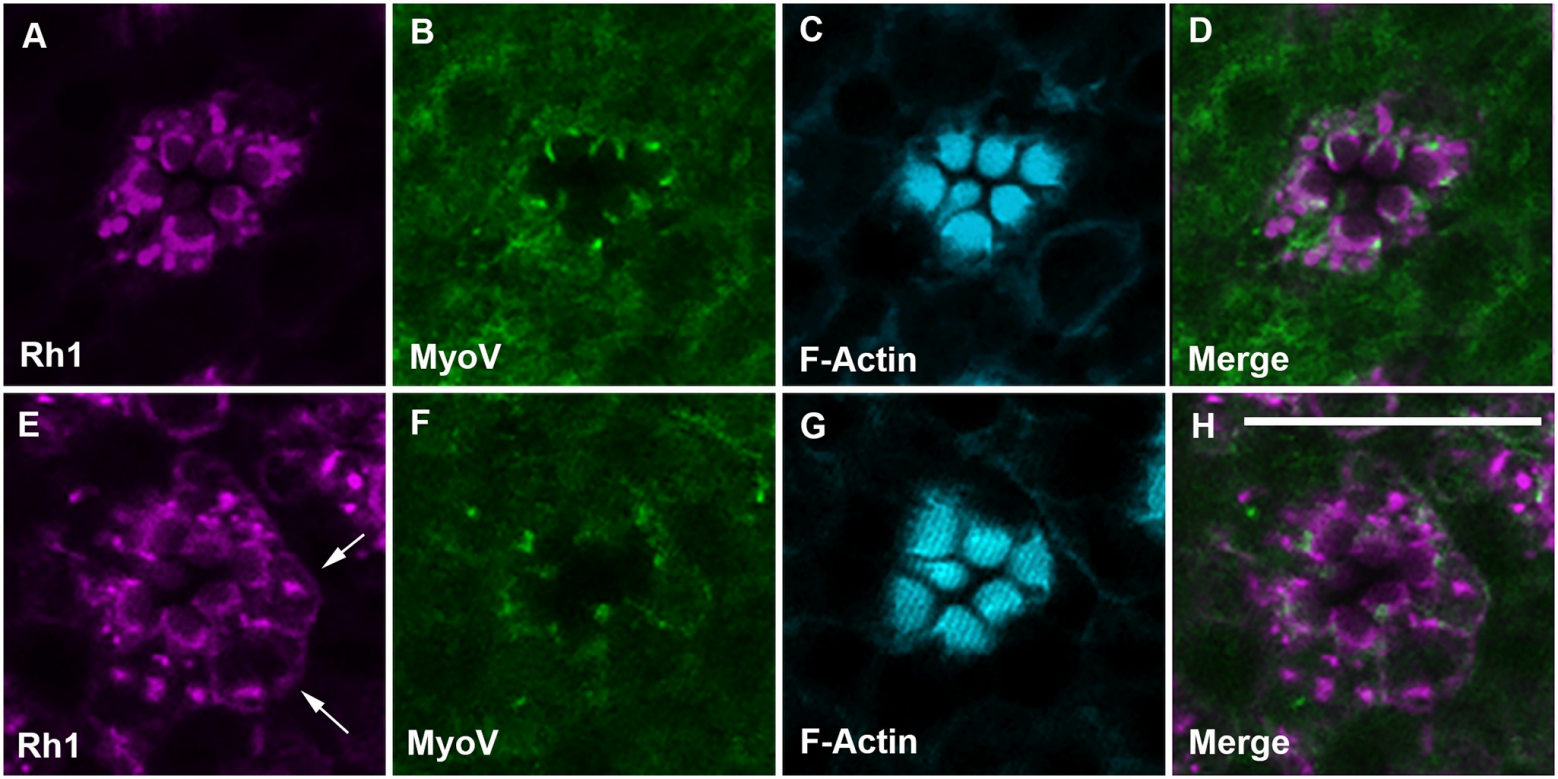
Rh1 is mislocalized in *PIP82* mutants. A-D. wild type, *w*^*1118*^, and E-H. *w*, *PIP82*^*1bpΔ*^ mutant photoreceptors stained for Rh1 (magenta) and MyoV (green) and F-Actin. The arrows highlight the distribution of Rh1 on the basal-lateral membrane. Each image is a single confocal section. All retinas are 1-day old and light exposed. Scale bar is 10uM.

Rhabdomere morphology and maintenance are dependent upon the correct delivery of material to the base of the rhabdomere at the interface between the rhabdomere terminal web and the cytoplasm. The delivery of secretory vesicles and Rhodopsin, for instance, is dependent upon MyoV/Rab11/dRip11complex. The disruption of this complex results in misshapen rhabdomeres but leaves the stalk membrane intact [46]. We therefore examined both Rhodopsin 1 (Rh1) and Myosin V (MyoV) expression in wild type and *PIP82* mutant photoreceptors. In light exposed wild type photoreceptors Rh1 and MyoV immunofluorescence is only detected at the base of the rhabdomere and on vesicles in the vicinity of the rhabdomere terminal web [46, 47]. We found that PIP82 does not colocalize with Rh1 and MyoV on vesicles but does colocalize with Rh1 localization at the base of the rhabdomere, the presumptive rhabdomere terminal web, (S7 Fig) in our fixation conditions [47]. In newly eclosed *PIP82* mutant photoreceptors, Rh1 and to a much lesser extent MyoV were no longer constrained to vesicles and the rhabdomere terminal web but accumulated improperly on the basal lateral membranes (Fig 7), despite overall seemingly normal levels of Rh1 (Fig 6C). The misdirection of material in the *PIP82* mutant was not limited to Rh1 but also included other components of the phototransduction machinery; Trpl but not Trp was found to colocalize with Rh1 throughout the basal/lateral membranes (S8 Fig). However, there was not a general defect in trafficking or localization of proteins. The localization of the alpha subunit of Na^+^K^+^ ATPase to the basal lateral membrane was unaffected in *PIP82* mutant photoreceptors (S9 Fig). Together, these data support the idea that PIP82 localization and limitation to the base of the rhabdomere could serve as a landmark for delivery of material to the apical surface. Alternatively, PIP82 may be required for the retention and stabilization of proteins targeted to the rhabdomeric membrane.

### The evolutionary emergence of PIP82 correlates with the origin of the open rhabdom of brachyceran flies

**Fig 8:**
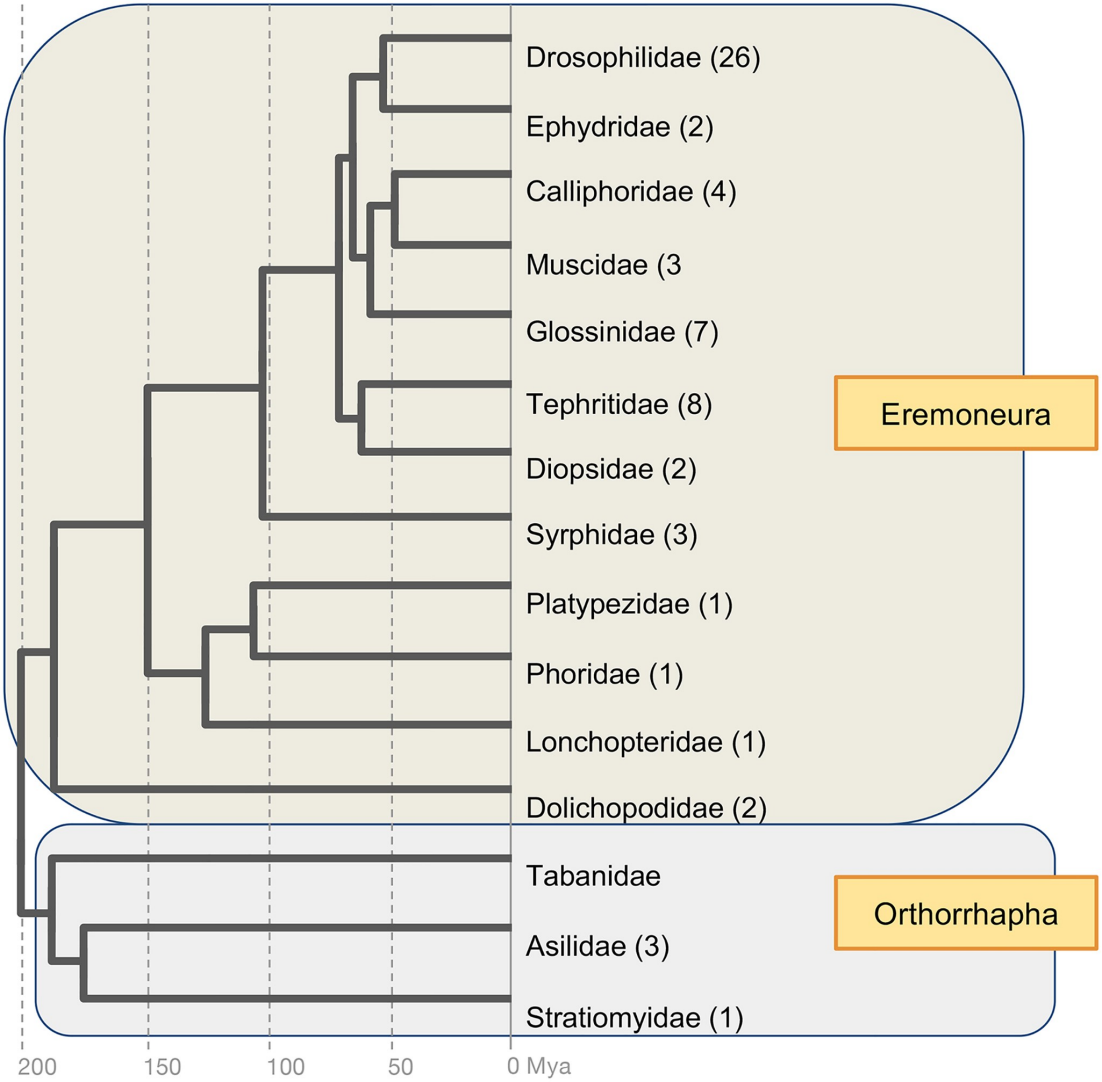
Phylogenetic analysis of PIP82 evolution. Taxonomic distribution of identified PIP82 orthologs. Numbers in parentheses represent the numbers of species in which PIP82 homologs were found. Time scale phylogeny based on Wiegmann et al. [81].

In *Drosophila*, the appearance of two distinct apical domains is a defining feature of the morphology and organization of the photoreceptors within each ommatidium and is observed in other apposition compound eyes with open rhabdoms [1, 13, 16, 48–50]. To elucidate the relationship between the evolutionary origins PIP82 and the open rhabdom organization of *Drosophila* ommatidia, which is a hallmark trait of brachyceran flies, we searched the NCBI RefSeq, TSA, and WGS databases for PIP82 homologs. Starting with the deduced amino acid sequence of *Drosophila* PIP82 as a query, these efforts yielded a compilation of over 60 high confidence singleton orthologs from 15 families representing the phylogenetic depth of brachyceran species diversity (Fig 8 and S1 File). Within this clade, most sequences were recovered from species representing the supertaxon Eremoneura, which includes *Drosophila*. However, three likely full-length sequences were also found in orthorrhaphan species, i.e. *Holcocephala fusca*, *Proctacanthus coquilletti*, both robber flies (Asilidae), and the soldier fly species *Hermetia illucens* (Stratiomyidae). Neither searches with *Drosophila* PIP82 nor one of its quite diverged orthorrhaphan orthologues produced evidence of significantly similar protein sequences in species outside the brachyceran clade. While surprising given the on average 1100 amino acids lengths of PIP82 homologs which exceeds the average of evolutionarily young *de novo* originated genes [51], this finding was further supported by the absence of detectable known protein sequence motifs as suggested by searches in the Pfam database (https://pfam.xfam.org/) with both eremoneuran and orthorrhaphan homologs of PIP82.

Interestingly, multiple sequence alignment revealed that the phospho-regulated basic and hydrophobic (PRBH) domain, responsible for regulated cortical membrane localization, was only conserved in a subset of the brachyceran PIP82 homologs, constituting the subclade Schizophora. Within the schizophoran homologs, serine 429 (based upon the *Drosophila melanogaster* sequence numbering) was strongly conserved as the likely phosphorylation target of aPKC (S10 Fig). The apparent lack of this domain in the more distantly related PIP82 homologs is probably best explained by significant evolutionary changes in the post-translational control of PIP82 function. Overall, these findings suggested that the *de novo* origin of PIP82 during early brachyceran evolution played a specific role in the structural emergence of the open rhabdom in the same superclade of flies about 200 million years ago. Consistent with this conclusion, TEM analyses in the red flour beetle *Tribolium castaneum* confirmed the entire apical domain of the photoreceptors in compound eyes with fused rhabdoms is defined by rhabdomeric microvilli only (S11 Fig). While the apical domain is defined by adherence junctions as seen in Drosophila, there is a complete absence of stalk membrane in agreement with the absence of PIP82 or PIP82-like homologs in the generally closed rhabdom species outside brachyceran Diptera.

Finally, to gain first insights into the cis-regulatory evolution of PIP82, we explored whether PIP82’s integrated enhancer and promotor organization with the *Nrg* locus in *Drosophila* is conserved in other species. Indeed, the homologs of both genes were similarly organized within the genome sequence drafts of the distantly related stable fly *Stomoxys calcitrans* (Muscidae) [52] and the even more distantly related Mediterranean fruit fly *Ceratitis capitata* (Tephritidae) [53]. In addition, both genes were located in the same contig of the high-quality genome sequence assembly of the robber fly species *P*. *coquilletti* (Asilidae) [54]. As Nrg predates the origin of dipteran insects and thus of PIP82 [55], these findings highlight a possibility that the photoreceptor-specific expression of PIP82 may have originated by the co-utilization of preexisting cis-regulatory elements in the brachyceran *nrg* locus that promote the described expression of *nrg* in photoreceptors [55–57].

## Discussion

In this study, we have characterized the *Drosophila* protein PIP82 [18]. Our and modEncode transcriptome data [24, 25, 29] suggested that PIP82 is a photoreceptor specific protein. Our immunofluorescence and transcriptional reporter data confirmed that PIP82 expression is only detected in photoreceptors. PIP82 is not required for specification of photoreceptors but rather is a terminal differentiation gene. Moreover, we demonstrate that PIP82 transcription is regulated by the known transcriptional drivers of photoreceptor differentiation Glass and Pph13 [25–28] and, functionally, that the loss of PIP82 impacts the morphological differentiation and maintenance of rhabdomere structure. We further show that PIP82 is a post-translational target of aPKC and phosphorylation of PIP82 regulates cortical membrane localization of the protein. In photoreceptors PIP82 is restricted to the rhabdomeric portion of the apical surface and upon the loss of the aPKC/Crumbs complex PIP82 expression is no longer limited to the rhabdomeric portion of the apical surface and extends between the adherens junctions.

Equally intriguing we find evidence that the evolutionary appearance of PIP82 in the Diptera correlated with the advent of open rhabdoms in brachyceran flies. Of note, the genetic, molecular and cellular studies of the adaptive transition from fused to open rhabdoms have not only identified key proteins associated with this transition but also the underlying developmental mechanisms [12–16]. Previous investigations into different adaptive morphologies have focused on changes in regulatory sequences or protein function [58–60]. Our recent data have presented a more complex picture with respect to the transition between fused and open rhabdoms by uncovering correlated changes in both regulatory sequences and protein function [17]. Here our study on PIP82 reveals a role of de novo protein emergence in promoting novel trait formation.

### PIP82 is unique to brachyceran Diptera

Analyses in several organisms suggest that the stochastic emergence of new open reading frames from non-coding DNA gives rise to novel genes, a process which is referred to as *de novo* gene evolution in contrast to gene duplication [61]. Moreover, *de novo* originated genes have been found to contribute to the generation of lineage specific adaptations (reviewed in [62–64]). Two interesting features of PIP82 suggest that the PIP82 is a de novo gene. First, PIP82 is devoid of any recognizable functional domains based upon sequence identity, lacking even small stretches of homology to other proteins. Second, PIP82 homologs are limited to brachyceran Diptera. PIP82 satisfies even a more stringent characteristic of *de novo* origination as defined by the failure to detect a homolog in a sister lineage despite conserved synteny of the de novo gene containing region [62, 63]. Given the close linkage of *PIP82* and *nrg*, which appears to be conserved in the brachyceran species, and the presence of *Nrg* homologs outside the brachyceran Diptera, the *de novo* emergence of PIP82 in early Brachycera seems also supported by this criterion. Besides the presence of candidate open reading frames, *de novo* gene evolution also requires the evolutionary emergence of transcriptional activation mechanisms. The conserved close linkage of PIP82 and *nrg* further suggests that it was the co-option of *nrg* regulatory sequences by PIP82, which facilitated the photoreceptor-specific expression of the latter.

As we have described, our results have demonstrated that PIP82 is regulated by a conserved set of transcription factors for rhabdomeric photoreceptor differentiation in insects, found in both open or fused rhabdom configurations [19, 21, 65]. Our data demonstrated the key regulator is the zinc finger protein Glass and a feed forward loop that requires the photoreceptor specific transcription homeodomain transcription factor Pph13 for maximum expression [30]. Intriguingly, the PIP82 transcript region and the upstream regulatory region that reproduces its photoreceptor specific expression are all located within the first intron of *nrg*. Nrg is a L1-type CAM cell adhesion molecule that is expressed in many cell types including neurons and the *Drosophila* photoreceptors [56, 57, 66]. Unfortunately, the regulatory regions of *neuroglian* have not been defined. Notwithstanding this, combined, our findings raise the intriguing possibility that PIP82 may have co-opted the photoreceptor regulatory regions of *nrg* to drive its own expression within photoreceptors.

### Integration of PIP82 into a signaling cascade and transition from functional to essential

Functionally, our results define PIP82 as a cortical photoreceptor protein and confirm PIP82 as a target of aPKC. The cortical localization is defined by stretches of basic hydrophobic amino acids [31] and PIP82 contains several potential aPKC binding sites (S1 and S10 Figs). Nonetheless, the combination of the two distinct features provides for a unique cellular function, aPKC regulated cortical localization. Additionally, the simplicity of generating the PRBH domain further supports the idea that PIP82 was not the result of a gene duplication or recombination event but rather *de novo* evolution. As presented in Bailey *et al*. [31], many different amino acid combinations can satisfy the electrostatic condition for binding phospholipids and an aPKC target sequence, which is simply defined by a four amino acid sequence of R/K-X-S-X-R/K. Therefore, an existing BH domain would only potentially need to accumulate a few mutations to generate linked aPKC phosphosites. Moreover, the PRBH domain is only one of a few conserved domains our comparison of homologs has now identified. Future work will functionally define these other domains and define the molecular mechanisms for the mislocalization of rhabdomeric proteins upon the loss of PIP82.

The PRBH domain permits PIP82 to integrate into an existing signaling cascade, but any *de novo* gene still needs to serve an advantageous function in order to be maintained in the genome by selection. Our phylogenetic data demonstrated a correlation with the lineage specific appearance of PIP82 and the adaptive transition from open and fused rhabdom organization. Besides the characteristic presence of inter-rhabdomeral space, TEM analysis of fused systems [13, 16, 17, 50, 67–71] showed that there is a difference in the apical membrane of photoreceptors in fused and open rhabdoms. As described, the apical membrane of open rhabdoms consist of two unique membranes: the stalk membrane and the microvilli of the rhabdomere. The stalk membrane is instrumental in positioning the rhabdomeres within each ommatidium for correct detection of light inputs. However, in fused rhabdoms this division does not seem to exist, as our analysis in *Tribolium* indicates. Instead, the entire apical surface, as defined by the adheren-like junctions, consists of rhabdomere microvilli. The combined absence of a stalk membrane and PIP82 in closed rhabdom photoreceptors suggests that PIP82 may be instrumental in supporting this morphological difference.

Also, the functional characterization of PIP82 supports this hypothesis. The presence of two different apical domains requires mechanisms for differential delivery and retention of proteins as well as mechanisms to demarcate and maintain the boundary between the two membrane compartments. Our immunofluorescence data demonstrate that PIP82 localizes to the base of the rhabdomere and its localization can be regulated by aPKC. Given that aPKC localization to the stalk membrane is dependent upon Crumbs, this result highlights a mechanism in which Crumbs, via the recruitment of aPKC, can modulate the presence of cortical membrane proteins and maintain a defined boundary between the two different apical membranes. Functionally, the loss of PIP82 does not lead to the loss of either photoreceptor apical domain. Instead, we observe that the rhabdomere shape is altered upon eclosion and is not maintained. In order for apposition compound eyes with neural superposition to interpret visual cues correctly, each photoreceptor rhabdomere must obtain and maintain a stereotypical position and shape in each ommatidium [4, 48, 49]. Otherwise each lamina cartridge is no longer detecting the neural convergence of one point in space but rather many different points. Therefore, defects in rhabdomere shape and the inability to maintain shape can potentially lead to defects in the ability to see, e.g. motion detection and visual acuity as seen in mutants of *eys* [13, 72]. Second, we observed the mislocalization of rhabdomeric proteins, in particular phototransduction proteins. This is despite the photoreceptors having normal apical/basal polarity as observed by the proper localization of Crumbs and alpha subunit of Na^+^K^+^ ATPase. The mislocalization of the phototransduction machinery does not prevent the light response, but may enhance response amplitude through potential regulatory changes. Alternatively, or in addition, this effect may be the result of altered rhabdomere morphology. These issues can be clarified in subsequent experiments, along with the cellular mechanism responsible for trafficking/retention defects of specific rhabdomeric proteins in *PIP82* mutants.

Overall, our characterization of PIP82 has now provided a molecular and cellular foundation from which we can trace the birth of the gene, through transcriptional activation, the acquisition of functionality and finally incorporation into a developmental cellular pathway that potentially reinforces a lineage-specific adaptation.

## Methods

### *Drosophila* strains and genetics

All stocks and crosses were maintained and staged at 25°C on a 12 hr light/dark cycle in a light illuminated incubator. The following stocks were utilized in this study: *Pph13-Gal4* [21], *Pph13*^*hzy*^ [28], *w*^1118^ (RRID:BDSC_3605), *Df(1)BSC592* (RRID:BDSC_25426), *gl*^*3*^ (RRID:BDSC_508), and *gl*^*60j*^ (RRID:BDSC_509), *y*,*w ey-Flp*; FRT82B *l(3)cl-R3* /TM6B and *w*+; FRT82B *crumbs*^11A22^/TM6B. The following stocks were created for this study: UAS-PIP82. cDNA clone HL06608 (DGRC #1287046) was inserted into pUASt and constructs were randomly integrated (Rainbow Transgenic Flies, Inc) and for rescue experiments an insertion on the third chromosome was utilized. To generate *PIP82*^*1bpΔ*^ the following sgRNA was generated 5’-GCAGGAGGAGGTACAGCGGG-3’ and cloned into pU6-2-BbsI-gRNA (DGRC #1363) and then subsequently injected into *w*^1118^; vas-Cas9 (RRID:BDSC_51324, Rainbow Transgenics). All the F2 progeny containing the original targeted X chromosome were screened by PCR coupled with DNA sequencing (PCR primers– 5’- CCTTAGCCAAATTGCCACAACTAAAGTTGC-3’ and 5’-GCTCACCCTCGTACATTATTAGCGAACTGATGG-3’. PIP82-nLacZ: a 2 Kbp region upstream of (5’ primer 5-GACGTGAGTAATGCGATTTACTTAACTTG-3 and 3’ primer 5-GGCACCAAGCTAGCCCAGATCTATTTAACT-3) was cloned into pLacZattB and integrated into genomic position 25C6 (attP40) (Rainbow Transgenics Flies, Inc).

### Transmission electron microscopy and immunofluorescence staining

All procedures (TEM and Immunofluoresence) were performed as previously described [14, 15]. The primary antibodies used in this study were: mouse anti-elav (9F8A9, 1:200, Developmental Studies Hybridoma Bank); mouse anti-Crumbs (Cq4, 1:100, Developmental Studies Hybridoma Bank), rabbit anti-aPKC ζ (zeta) (1:200, SAB4502380 Sigma), mouse anti-HA (6E2, 1:500, Cell Signaling), mouse anti-myc (9B11, 1:500 Cell Signaling), rat anti-Crumbs (1:500) [73], mouse anti-armadillo (N2 7A1, 1:100, Developmental Studies Hybridoma Bank). The C-terminal peptide KVNKLISRFEGGRPRLC (produced in the laboratory of Dr. Charles Zuker) was used as the antigen in rabbits, and the resulting antibody was used at a concentration of 1:200. Fluorescent conjugated secondary antibodies were obtained from either Jackson ImmunoResearch Laboratories or Life Technologies. Rhodamine or Alexa Fluor 647 conjugated phalloidin (1:200, Life Technologies) were utilized for the detection of F-Actin. Confocal images were captured on a Leica TCS SP5. TEM imaging was conducted with a JOEL 1010 and JOEL 1400. All images were processed in Adobe Photoshop.

### Membrane measurements and analysis

TEM images were taken of individual ommatidium in eyes from three different animals for each genotype at similar depths. Stalk membranes and the bases of rhabdomeres of R3, R5, and R7 were traced and measured using the segmented line and measure tools in ImageJ (https://imagej.nih.gov/ij/), respectively. Two measurements were made for each photoreceptor: 1) the total membrane from one adherens junction to the other including the base of the rhabdomere, and 2) the base of the rhabdomere alone. Stalk membrane length was determined by subtracting length of the base of the rhabdomere from the total. Measurements were made of at least 7 different ommatidia, and means were obtained for R3/R5 and R7 of each eye. The means were compared using one-way ANOVA and Tukey’s HSD post-hoc tests in JMP (SAS). Graphs of stalk membrane and rhabdomere base lengths were generated using GraphPad PRISM software.

### Serial block face

Drosophila heads were fixed in 4% formaldehyde, 3.5% glutaraldehyde, 100 mM cacodylate buffer (pH 7.4), 2 mM Calcium chloride overnight at 4°C as previously described [74]. For further processing of the tissue the NCMIR protocol SBEM Protocol v7_01_10 was followed with few changes. After fixation the samples were rinsed 3X in ice cold 0.1M cacodylate buffer containing 2mM calcium chloride. The samples were incubated for 1 hour, on ice in the solution containing equal volumes of 4% osmium tetraoxide and 3% potassium ferrocyanide in 0.3M cacodylate buffer with 4 mM calcium chloride. The samples were washed with ddH_2_O 3X at room temperature (RT) followed by incubation in freshly prepared 10% thiocarbahydrazide solution for 20 minutes at RT. The samples were post fixed in 2% aqueous Osmium tetraoxide for 30 minutes in RT after washes in ddH_2_0. Samples were rinsed in ddH_2_O and then placed in 1% aqueous uranyl acetate solution overnight at 4°C. After washing the samples in ddH_2_O, the samples were en bloc stained with Walton’s lead aspartate stain [75] for 30 min at 60°C. After washing the samples in ddH_2_O the samples were dehydrated with the graded series of ice-cold ethanol from 30% to 100% for 10 min on ice and then twice with propylene oxide at RT. The samples were infiltrated with 50% each of propylene oxide and Epon resin hard for three hours and then 100% resin for three hours. The heads were embedded in 100% resin and left to polymerize at 60°C for at least 24 hours. The resin blocks were trimmed to a square surface of 500um X 500um. Once the block is trimmed to a t-shape it was cut off from the rest of resin. The sample was glued on the aluminum pins using the two-component silver conductive epoxy. The epoxy was coated all around the sample to reduce charging and also sputter coated with 5nm layer of gold. Then the block is surfaced using diamond knife in an ultramicrotome. Samples were placed in FEI Teneo microscope. The blocks were sectioned at 80nm thickness and the images were obtained at 2.5 kV at low vacuum pressure of 50pa. The images were processed utilizing AMIRA software (ThermoFisher Scientific).

### Electroretinograms

Electroretinogram recordings were carried out immobilized white-eyed flies. Glass electrodes were filled with normal saline (0.9% NaCl, w/v). Light stimulation was by means of a xenon arc lamp (300 W USHIO, Newport Corp., Franklin, MA). The light beam was passed through a series of infra-red cut-off, neutral density, and narrow bandpass filters, followed by a shutter and then presented to the fly through a liquid light guide. The ERG signals were amplified with a World Precision Instruments (Sarasota, FL) Model DAM 50 amplifier. The instrument was controlled, and data were acquired, by a Macintosh computer (Apple Computer, Inc., Cupertino, CA) equipped with a National Instruments (Austin, TX) PCI-MIO-16XE-50 multi-function input/output board running LabView software. Quantitative comparisons of response amplitudes were calculated from the difference between baseline voltage 0.5 sec before stimulus onset and the voltage at maximum depolarization. A minimum of two recordings from each of the two animals from each genotype were used in the calculations. Statistical significance between strains for responses at each light intensity were calculated using a Student’s T-test.

### Cell transfections and westerns

Cell transfection assays and immunofluorescence were performed as previously described [14, 16], utilizing Qiagen Effectene. Cells were transfected Tub-Gal4 [76], pUASt-PIP82, aPKC-myc [31]. S2 DGRC cells were obtained from the Drosophila Genomics Resource Center (DGRC #6). Cell tissue extract was generated as described in [14] in the presence of Halt phosphatase inhibitor cocktail (#78420 Thermo Scientific). For quantification of PIP82 localization pattern, three individual transfections were performed. Each transfection contained Tub-Gal4, pUASt-PIP82, and aPKC-myc. After 24 hours, each individual transfection was split into two wells and Cu^2+^ was added only into one well of each pair. After an additional 24 hours the cells were fixed and imaged for the localization of PIP82. For each paired transfection 60 cells were scored in each condition for the localization pattern of PIP82. All samples were countered stain to ensure the absence or presence of aPKC-myc in each Cu^2+^ condition. *Drosophila* head extract was generated as previously described [16]. Proteins were separated on Mini-Protean TGX gels (BIO-RAD) and transferred to Immobilon membranes (Millipore). Antibodies utilized were and mouse monoclonal anti-alpha Tubulin (1:2500, T9026-Sigma), rabbit anti-PIP82 (1:1000), rabbit Phospho-(Ser) PKC substrate Antibody (1:1000, 2261-Cell Signaling). Signal detection for westerns was achieved with use of a HRP-conjugated anti-mouse or anti-rabbit secondary antibody (1:5000) (Jackson ImmunoResearch Laboratories) combined with Superscript West Pico Chemiluminescent Substrate (Thermo Scientific).

Rh1 western: Western blot analysis was performed as described [77]. Protein extracts from ten heads were separated by SDS-PAGE (using a Bio-Rad Mini PROTEAN Tetra Cell) and electro-transferred (using a Bio-Rad Criterion Blotter). The immuno-blot PVDF membrane (Bio-Rad) was incubated simultaneously with mouse monoclonal anti-Rh1 antibody (4C5, Developmental Studies Hybridoma Bank) and rabbit polyclonal anti-actin (Abcam ab1801) overnight at room temperature. The immunoreactive proteins were detected with polyclonal goat anti-mouse IgG (H+L) conjugated to IRDye 800CW and polyclonal goat anti-rabbit IgG (H+L) conjugated to IRDye 680CW (both from LI-COR Biosciences). The blots were scanned with a ChemiDoc MP Imaging System (Bio-Rad).

### PIP82 homolog search and analysis

PIP82 homlogs were identified by pBLAST searches of the NCBI non-redundant protein sequence (nr) and tBLASTn searches of the NCBI whole genome shotgun contigs (wgs) and transcriptome shotgun assemblies (TSA) [78]. Candidate homologs were confirmed by the reciprocal best BLAST hit analysis [79] as well as analysis of multiple protein sequence alignments generated by T-Coffee [80]. The accession numbers of all identified PIP82 protein sequences and PIP82 containing contigs and transcripts are available in data file S1.

## Supporting information

S1 Fig Schematic and resulting change of the CRISPR/CAS9 induced *PIP82*^1bpΔ^ mutant allele. A. Localization of targeted region. B. Potential open reading frame of *PIP82*^1bpΔ^ mutant allele. The targeting of the second exon resulted in a single base pair deletion resulting in a frame shift truncating the protein to only 107 aa with additional 38 unrelated amino acids. C. The amino acid sequence of the phosphor-regulated basic and hydrophobic (PRBH) domain as predicted from Bailey *et al*. [31].
(TIF)

S2 Fig Examples of PIP82 localization in the presence or absence of aPKC. PIP82 is in green and aPKC is in magenta. aPKC expression was induced in the presence of Cu^2+^. Each image is a single confocal section. Scale bar is 25uM.
(TIF)

S3 Fig Stalk membranes and rhabdomere bases are shorter in *PIP82* mutant photoreceptors. A. TEM images of *w*^*1118*^ and *w*, *PIP82*^*1bpΔ*^ retinas with stalk membranes (red), and bases of rhabdomeres (yellow) indicated. B. R3/R5, and R7 stalk membrane lengths of *w*^*1118*^ and *w*, *PIP82*^*1bpΔ*^ photoreceptors. C. Bases of rhabdomeres of R3/R5, and R7 of *w*^*1118*^ and *w*, *PIP82*^*1bpΔ*^ flies. * p < 0.05, ** p < 0.01, *** p < .001 by Tukey’s HSD post-hoc test.
(TIF)

S4 Fig PIP82 mutant photoreceptors have no apparent defect in the ultrastructure of the rhabdomere terminal web, stalk membrane, or adherens junctions. Transmission electron microscopy of wild type, *w*^*1118*^, (A-A’) and w, *PIP82*^*1bpΔ*^ mutant photoreceptors (B-B’). Scale bars are 2um and 500nm.
(TIF)

S5 Fig Electroretinogram response amplitudes of wild type and PIP82 mutant photoreceptors. Electroretinogram response amplitudes were measured for 7-day old *w*^*1118*^ controls, dotted line, and *w*, *PIP82*^*1bpΔ*^ mutants, solid lines. Flies were stimulated with increasing intensities of light (from -5.0 attenuation to -1.0 log attenuation) at 470 nm, as in Fig 6. Error bars indicate +/- standard deviations of the mean amplitudes for *w* controls (gray) and *w*, *PIP82* mutants (black). Asterisks indicate statistically significant differences between the two genotypes at -2 and -1 log attenuation, *p* = 0.003 and p = 0.004 respectively. Replicates for each intensity were *w* controls n = 6 and *w*, *PIP82* mutants n = 5.
(TIF)

S6 Fig aPKC localization in wild type and *PIP82* mutant photoreceptors. A-C. wild type, *w*^*1118*^, and D-F. w, *PIP82*^*1bpΔ*^ mutant photoreceptors stained for aPKC (green) and F-Actin (magenta). Each image is a single confocal section of a 1-day old light exposed retina. Scale bar is 10uM.
(TIF)

S7 Fig PIP82 localizes to the base of the rhabdomere and colocalizes with Rh1 in adult photoreceptors. A-C. Wild type adult photoreceptors, *w*^*1118*^, stained for PIP82 (green), Rh1 (magenta), and F-Actin (Cyan). D,E represent merged images of PIP82 and Rh1 (D) and all three proteins (E). Each image is a single confocal section of a 1-day old light exposed retina. Scale bar is 10uM.
(TIF)

S8 Fig Localization patterns of Trp and TrpL in *PIP82* mutants. A-D, I-L. wild type, *w*^*1118*^, and E-H, M-P. *w*, *PIP82*^*1bpΔ*^ mutant photoreceptors stained for Trp (green—A,C,G,E) or Trpl (green—I,K,M,O), Rh1 (magenta) and F-Actin (cyan). Each image is a single confocal section of a 1-day old light exposed retina. Scale bar is 10uM.
(TIF)

S9 Fig Localization of Na^+^K^+^ ATPase in wild type and mutant photoreceptors. A. wild type, *w*^*1118*^, and B. *w*, *PIP82*^*1bpΔ*^ mutant photoreceptors stained for Na^+^K^+^ ATPase (green) and F-Actin (magenta). Each image is a single confocal section of a 1-day old light exposed retina. Scale bar is 10uM.
(TIF)

S10 Fig Sequence alignment of the PRBH among the subclade of schizophoran homologs. Astericks represent the two of three aPKC phosphorylation sites in the *Drosophila melanogaster* homolog. The boxed Serine (position 429 in *Drosophila melanogaster*) represents the conserved aPKC phosphorylation among homologs.
(TIF)

S11 Fig Transmission electron microscopy of Tribolium ommatidium. A-C Wild type ommatidium of v^W^ adult Tribolium. B and C represent higher magnifications of regions shown in A. The adherence/septate junctions between photoreceptors are highlighted with circles. Scale bar is 10uM and 1uM.
(TIF)

S1 Movie Serial block face scanning electron microscopy analysis of *PIP82* mutant photoreceptors. *w*, *PIP82*^*1bpΔ*^ mutant at 1-day post eclosion. Scale bars are marked on each movie and each movie samples ~ 300 80 nM sections thus covering a total depth of ~ 2.4 uM of the retina.
(MOV)

S2 Movie Serial block face scanning electron microscopy analysis of wildtype photoreceptors. *w*^*1118*^ at 7-day post eclosion. Scale bars are marked on each movie and each movie samples ~ 300 80 nM sections thus covering a total depth of ~ 2.4 uM of the retina.
(MOV)

S3 Movie Serial block face scanning electron microscopy analysis of *PIP82* mutant photoreceptors. *w*, *PIP82*^*1bpΔ*^ mutant at 7-day post eclosion. Scale bars are marked on each movie and each movie samples ~ 300 80 nM sections thus covering a total depth of ~ 2.4 uM of the retina.
(MOV)

S1 File Accession numbers and protein sequences of identified PIP82 homologs. (DOCX)
