# Supplementary figures and images for: The brachyceran de novo gene PIP82, a phosphorylation target of aPKC, is essential for proper formation and maintenance of the rhabdomeric photoreceptor apical domain in Drosophila
Source: PLoS Genet. 2020 Jun 24;16(6):e1008890. doi: 10.1371/journal.pgen.1008890 (PMC7340324; doi:10.1371/journal.pgen.1008890)

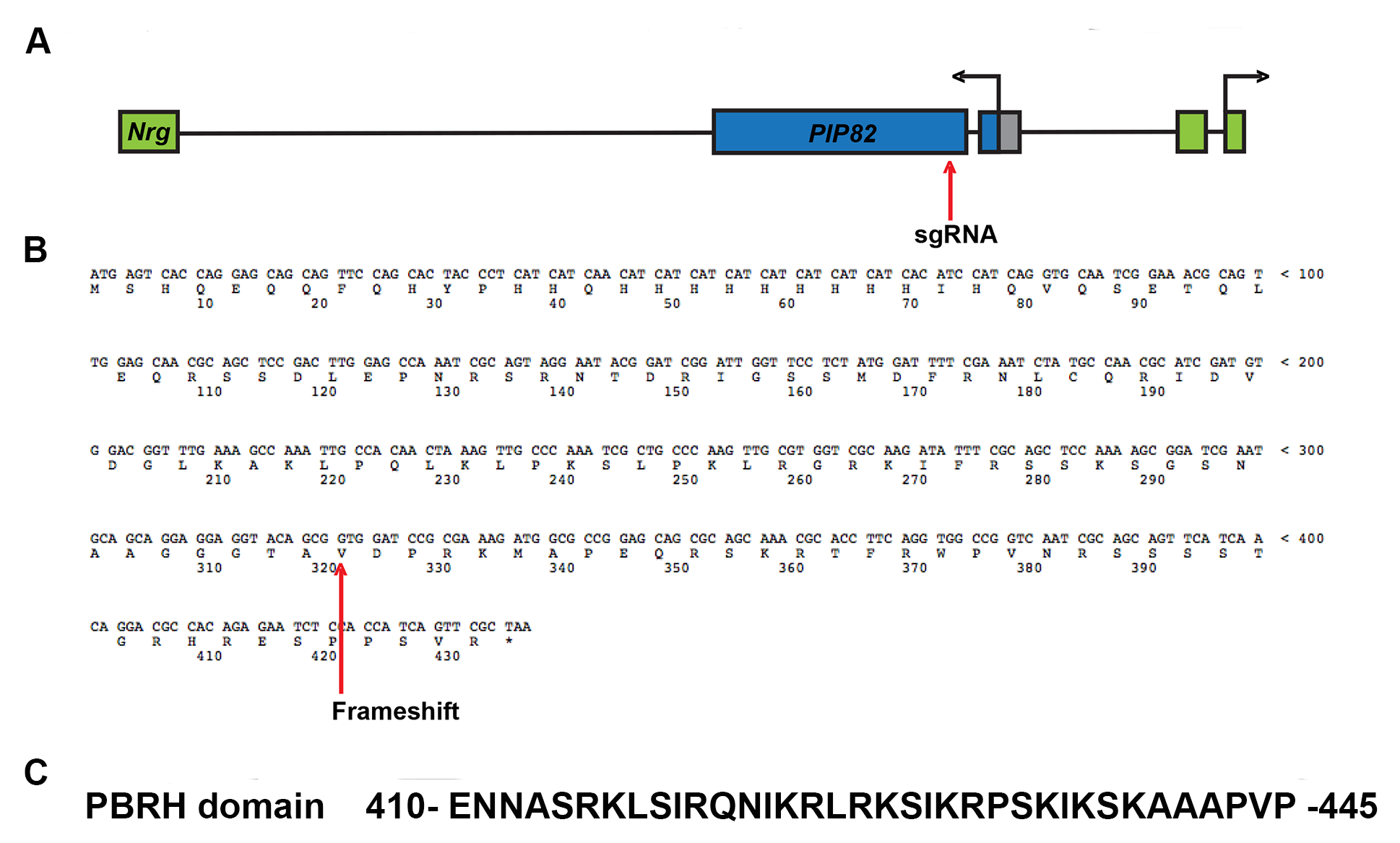

Supplement: S1 Fig — A. Localization of targeted region. B. Potential open reading frame of PIP821bpΔ mutant allele. The targeting of the second exon resulted in a single base pair deletion resulting in a frame shift truncating the protein to only 107 aa with additional 38 unrelated amino acids. C. The amino acid sequence of the phosphor-regulated basic and hydrophobic (PRBH) domain as predicted from Bailey et al. [31]. (TIF) [file pgen.1008890.s001.tif]

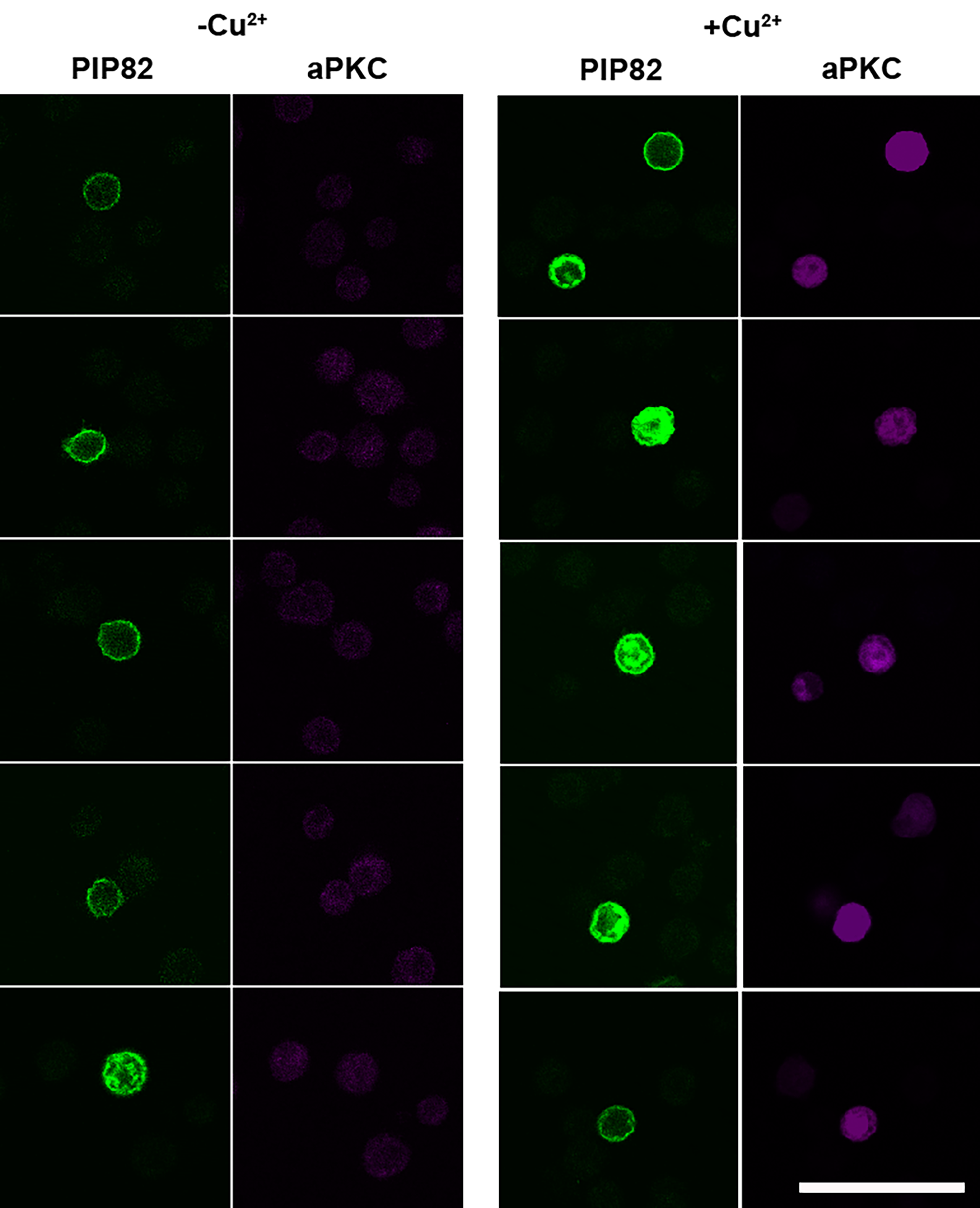

Supplement: S2 Fig — PIP82 is in green and aPKC is in magenta. aPKC expression was induced in the presence of Cu2+. Each image is a single confocal section. Scale bar is 25uM. (TIF) [file pgen.1008890.s002.tif]

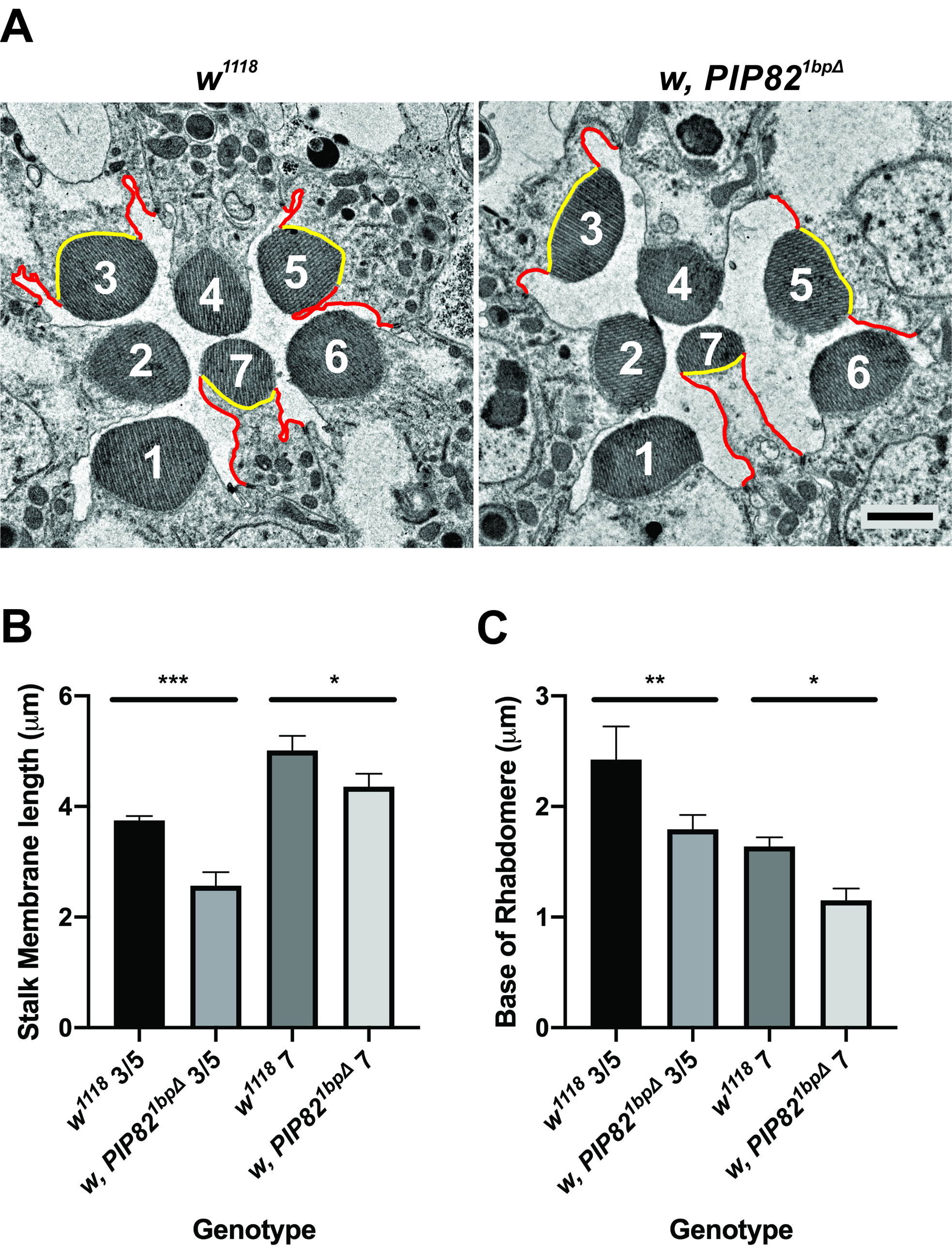

Supplement: S3 Fig — A. TEM images of w1118 and w, PIP821bpΔ retinas with stalk membranes (red), and bases of rhabdomeres (yellow) indicated. B. R3/R5, and R7 stalk membrane lengths of w1118 and w, PIP821bpΔ photoreceptors. C. Bases of rhabdomeres of R3/R5, and R7 of w1118 and w, PIP821bpΔ flies. * p < 0.05, ** p < 0.01, *** p < .001 by Tukey’s HSD post-hoc test. (TIF) [file pgen.1008890.s003.tif]

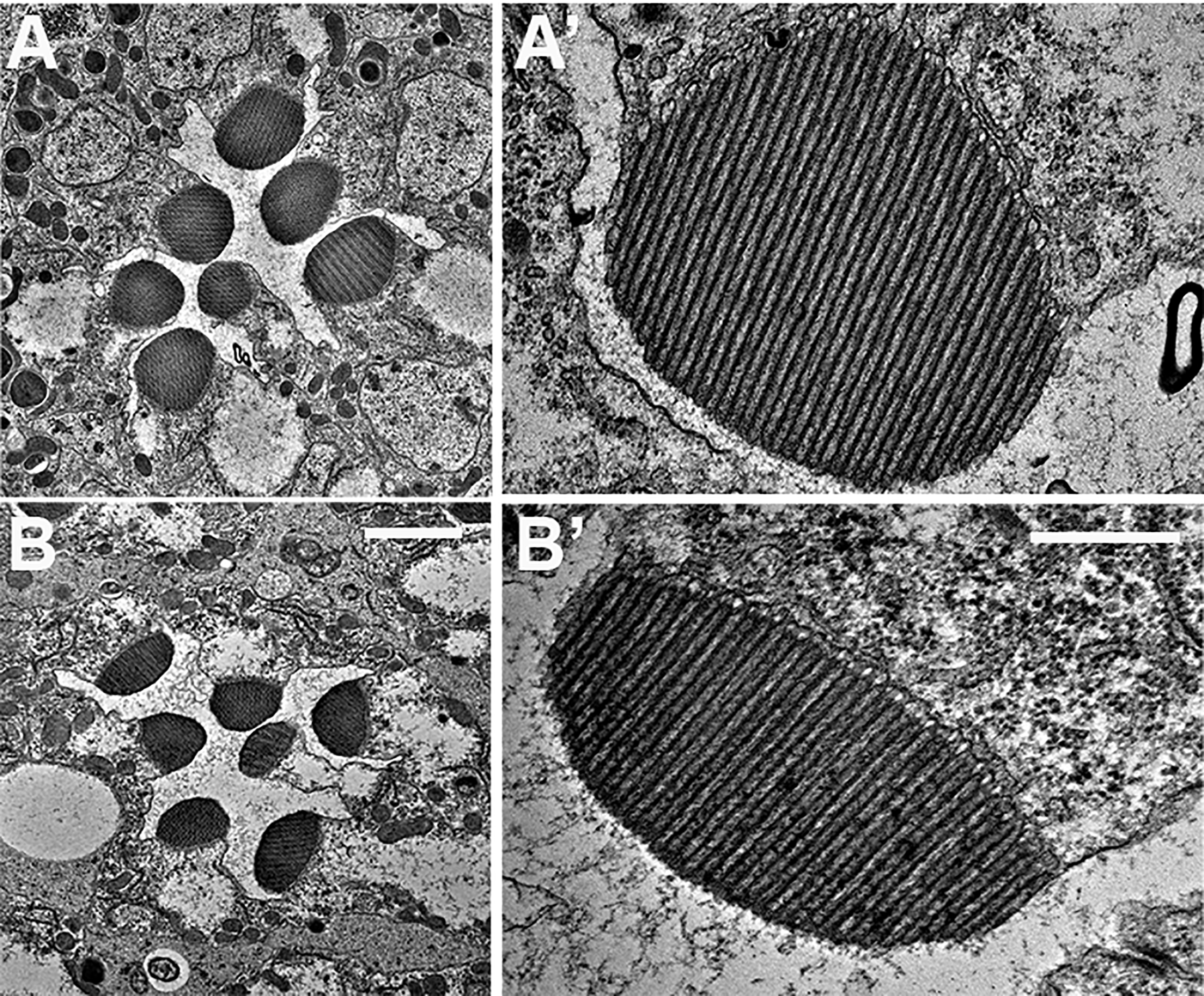

Supplement: S4 Fig — Transmission electron microscopy of wild type, w1118, (A-A’) and w, PIP821bpΔ mutant photoreceptors (B-B’). Scale bars are 2um and 500nm. (TIF) [file pgen.1008890.s004.tif]

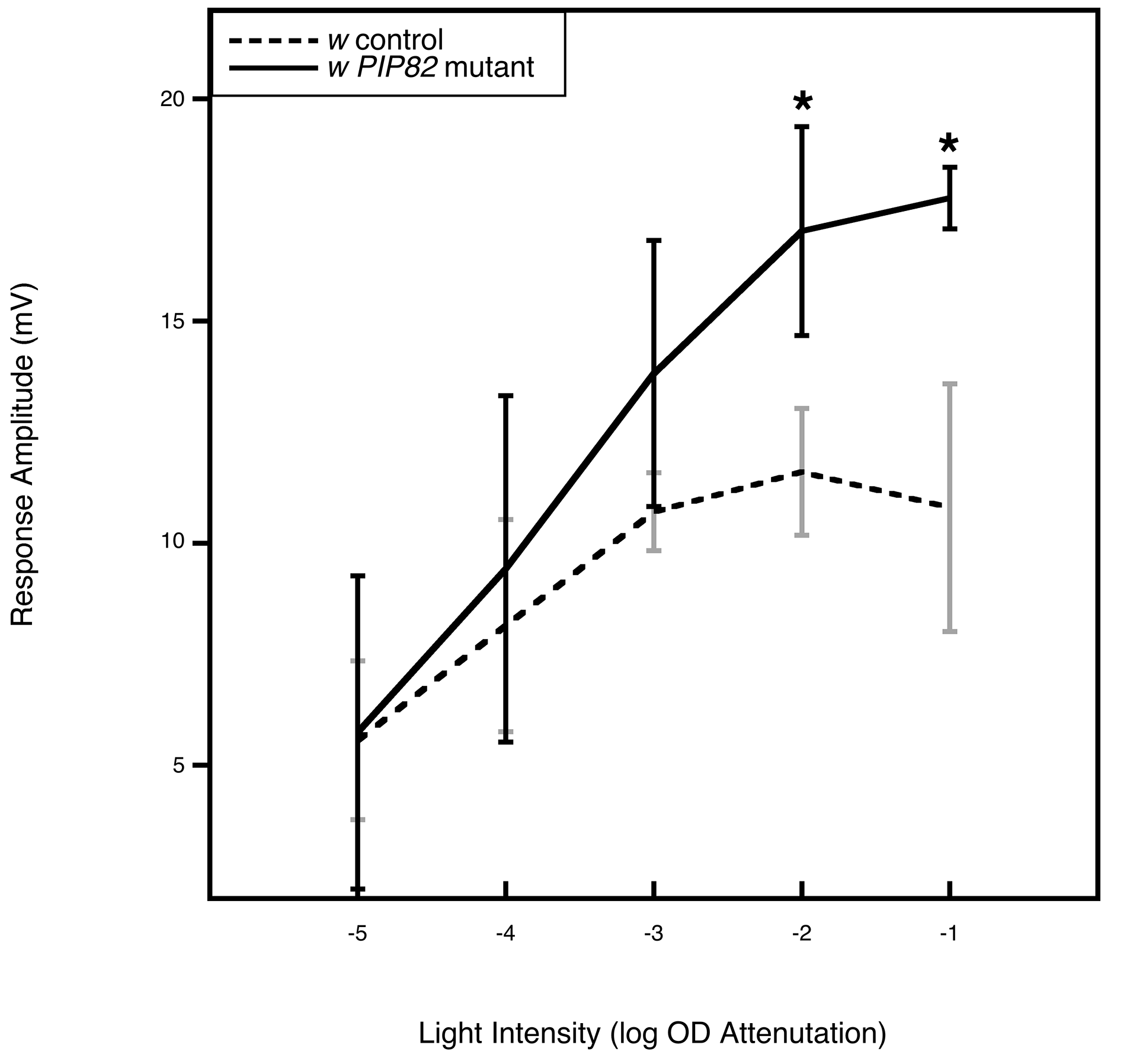

Supplement: S5 Fig — Electroretinogram response amplitudes were measured for 7-day old w1118 controls, dotted line, and w, PIP821bpΔ mutants, solid lines. Flies were stimulated with increasing intensities of light (from -5.0 attenuation to -1.0 log attenuation) at 470 nm, as in Fig 6. Error bars indicate +/- standard deviations of the mean amplitudes for w controls (gray) and w, PIP82 mutants (black). Asterisks indicate statistically significant differences between the two genotypes at -2 and -1 log attenuation, p = 0.003 and p = 0.004 respectively. Replicates for each intensity were w controls n = 6 and w, PIP82 mutants n = 5. (TIF) [file pgen.1008890.s005.tif]

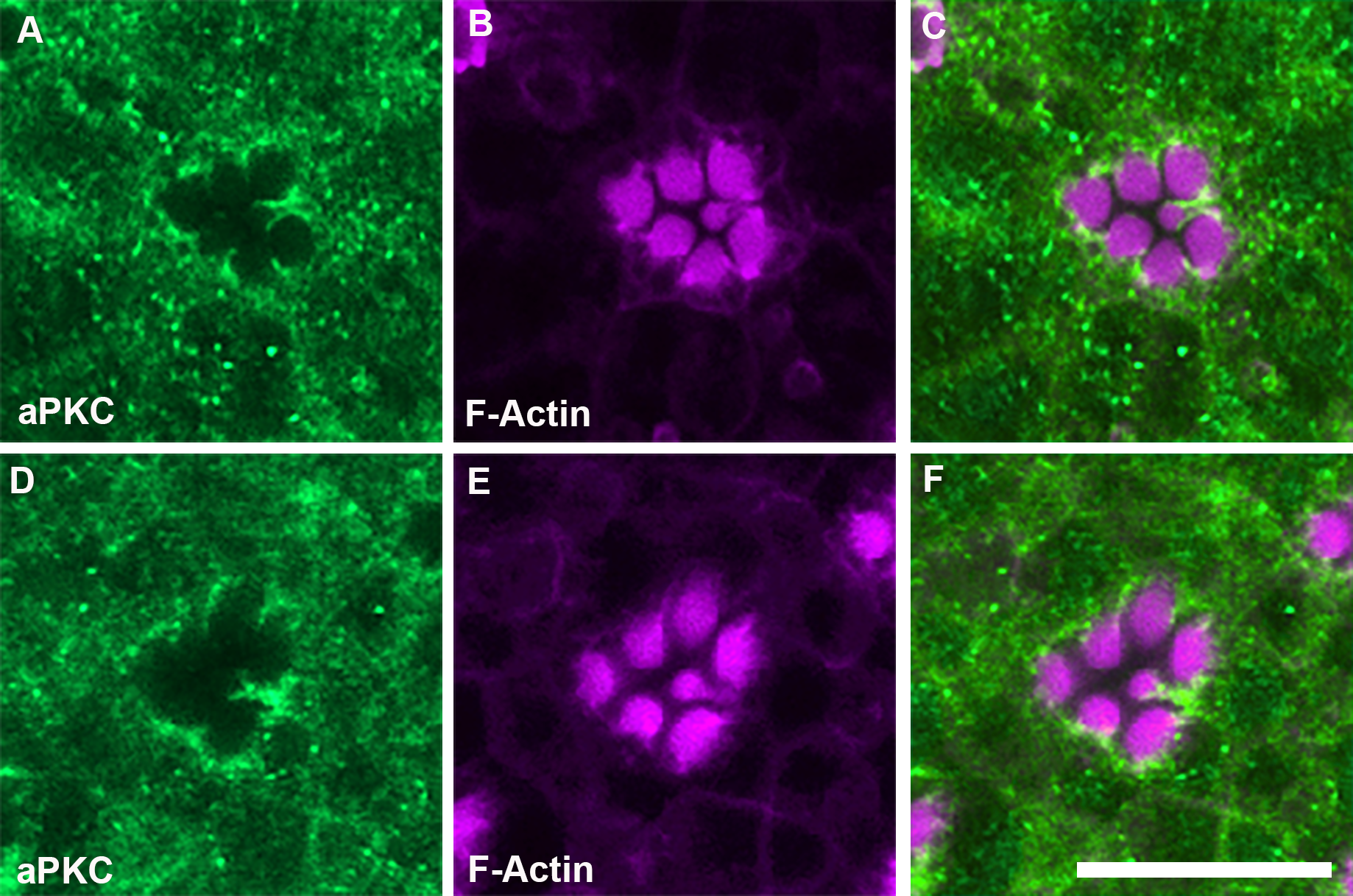

Supplement: S6 Fig — A-C. wild type, w1118, and D-F. w, PIP821bpΔ mutant photoreceptors stained for aPKC (green) and F-Actin (magenta). Each image is a single confocal section of a 1-day old light exposed retina. Scale bar is 10uM. (TIF) [file pgen.1008890.s006.tif]

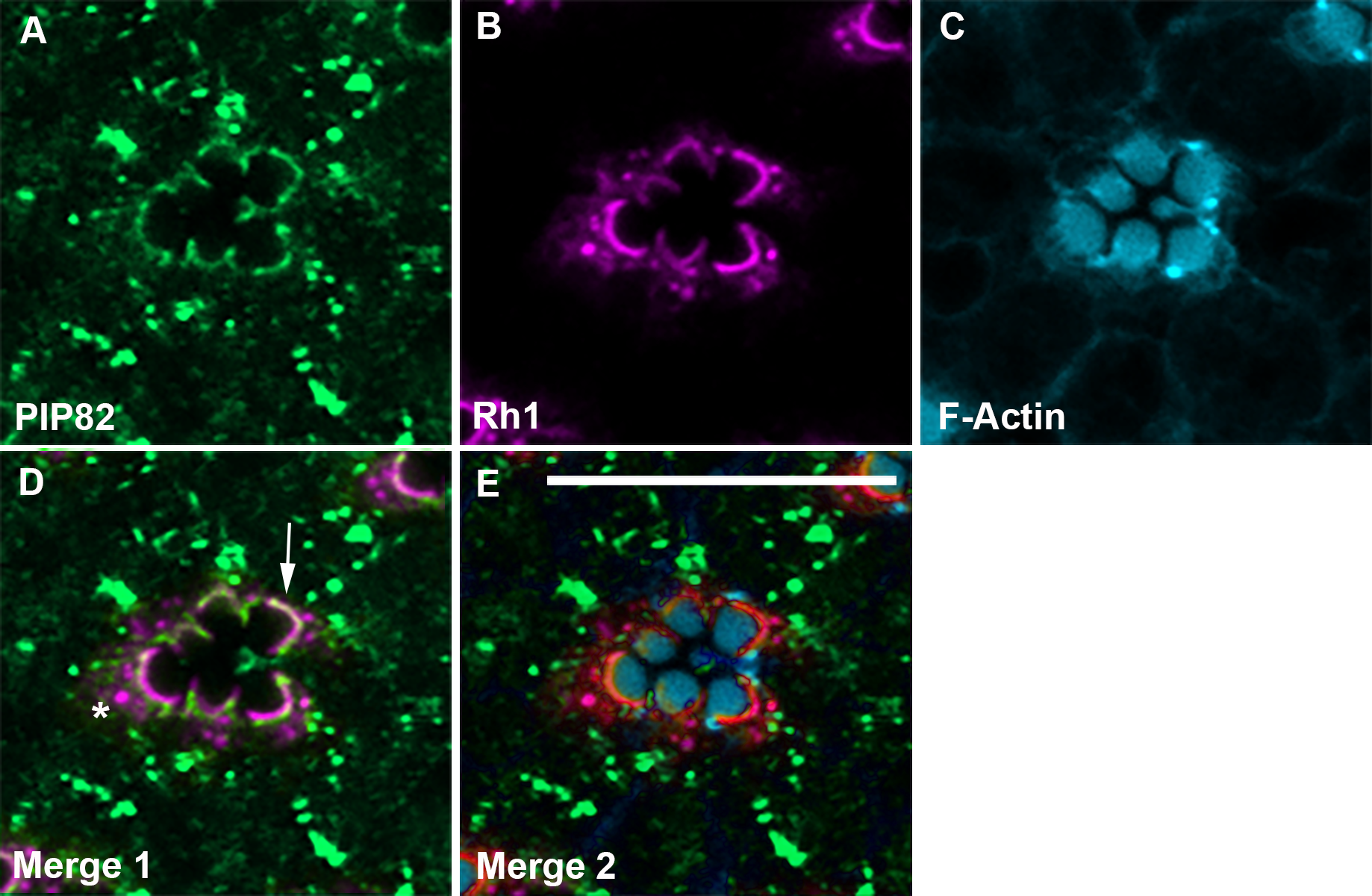

Supplement: S7 Fig — A-C. Wild type adult photoreceptors, w1118, stained for PIP82 (green), Rh1 (magenta), and F-Actin (Cyan). D,E represent merged images of PIP82 and Rh1 (D) and all three proteins (E). Each image is a single confocal section of a 1-day old light exposed retina. Scale bar is 10uM. (TIF) [file pgen.1008890.s007.tif]

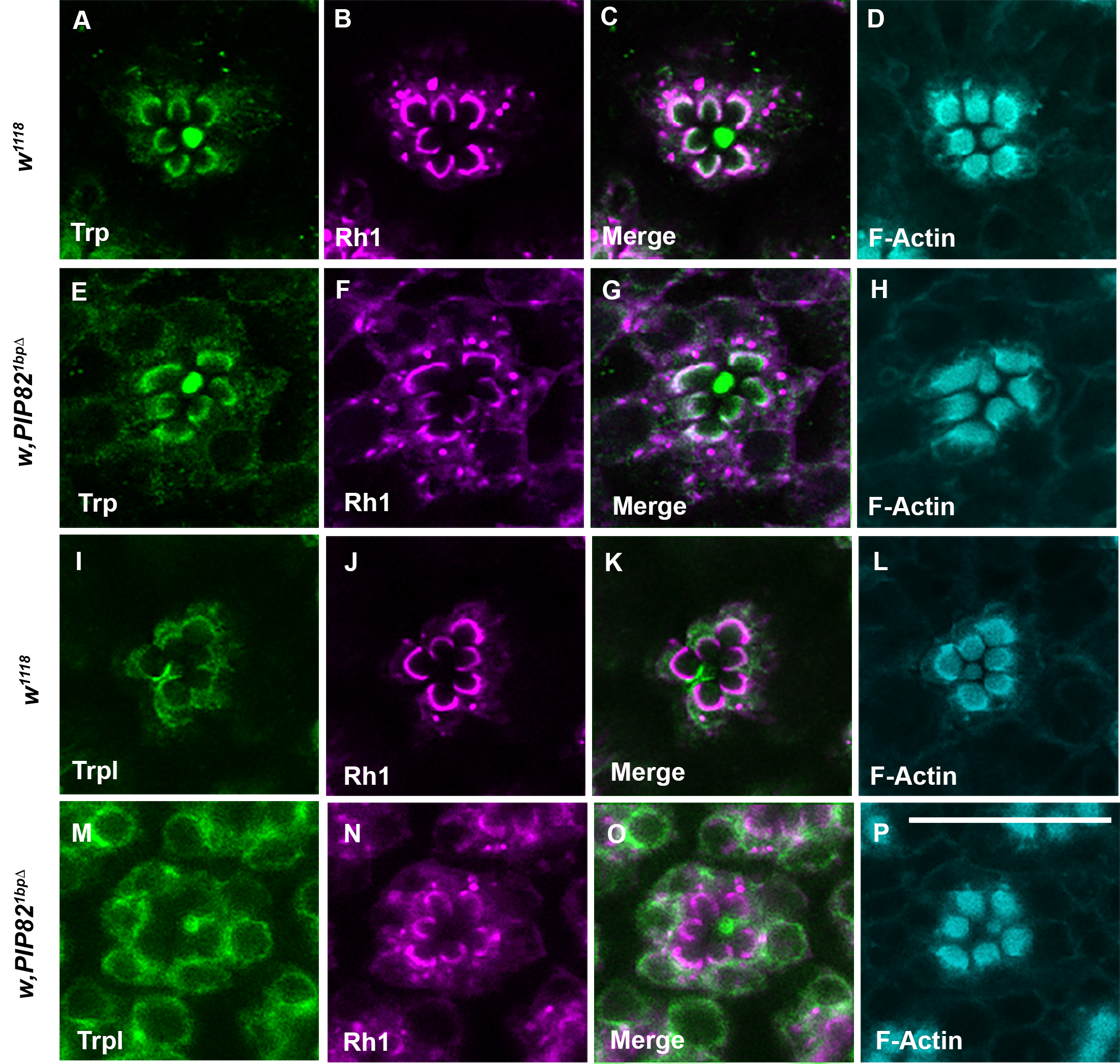

Supplement: S8 Fig — A-D, I-L. wild type, w1118, and E-H, M-P. w, PIP821bpΔ mutant photoreceptors stained for Trp (green—A,C,G,E) or Trpl (green—I,K,M,O), Rh1 (magenta) and F-Actin (cyan). Each image is a single confocal section of a 1-day old light exposed retina. Scale bar is 10uM. (TIF) [file pgen.1008890.s008.tif]

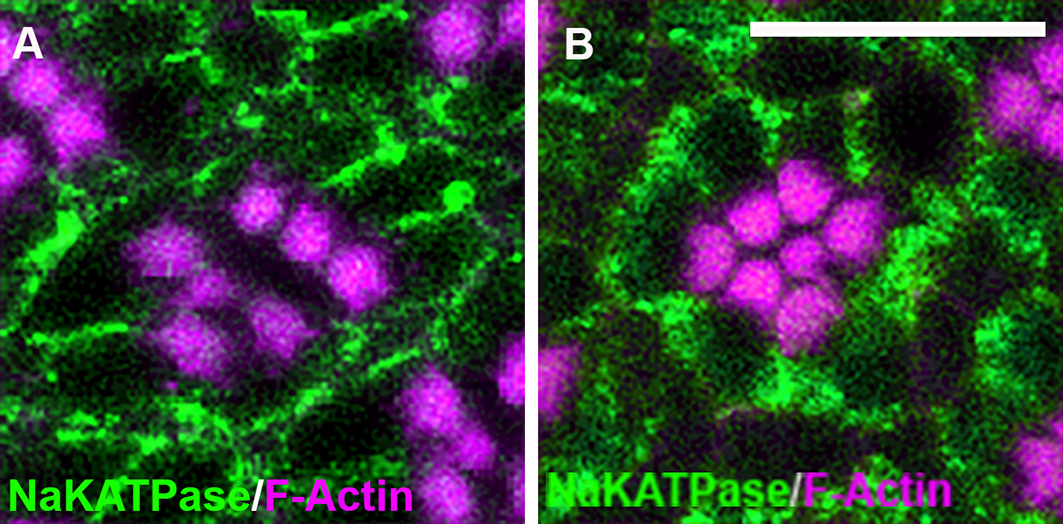

Supplement: S9 Fig — A. wild type, w1118, and B. w, PIP821bpΔ mutant photoreceptors stained for Na+K+ ATPase (green) and F-Actin (magenta). Each image is a single confocal section of a 1-day old light exposed retina. Scale bar is 10uM. (TIF) [file pgen.1008890.s009.tif]

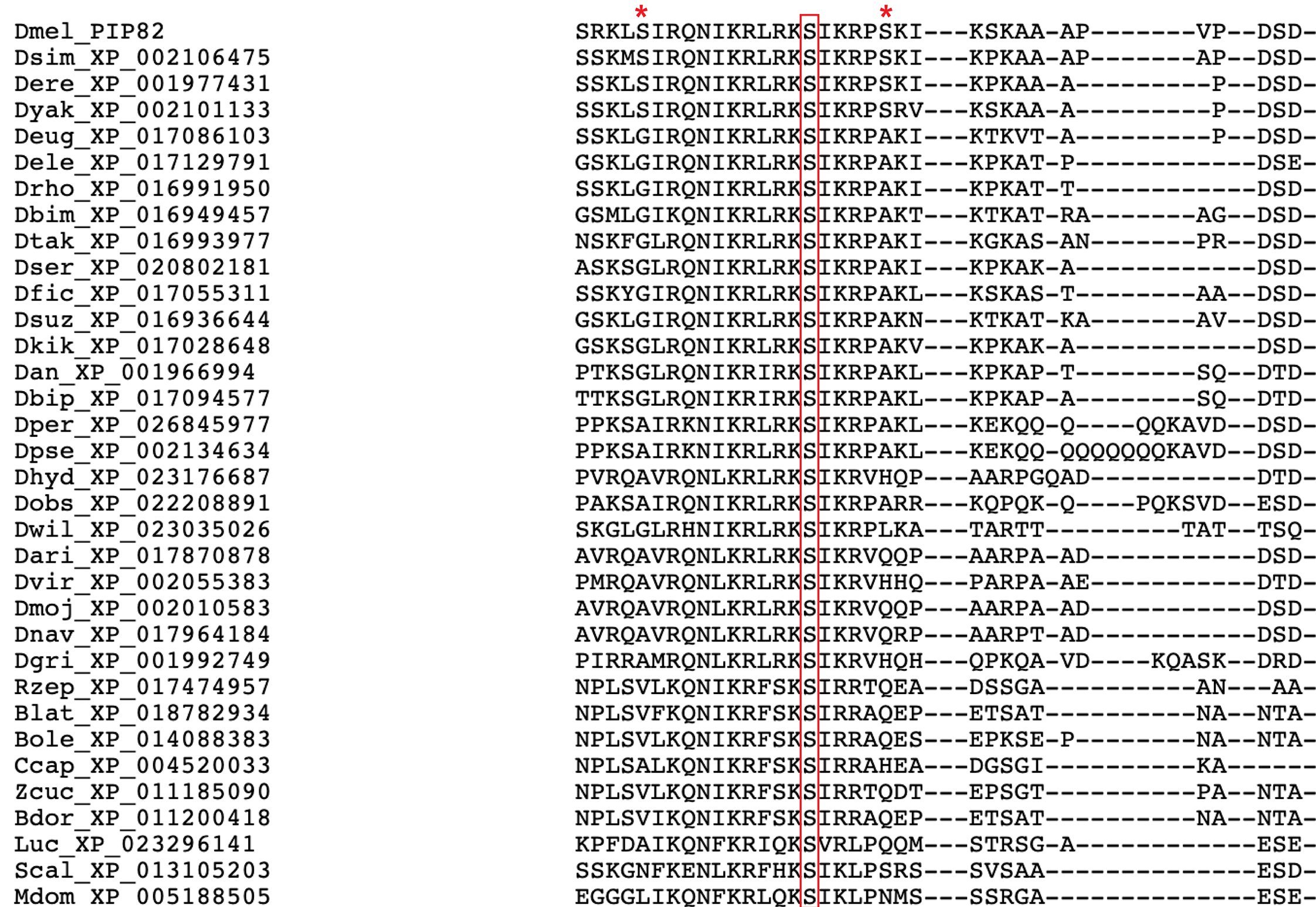

Supplement: S10 Fig — Astericks represent the two of three aPKC phosphorylation sites in the Drosophila melanogaster homolog. The boxed Serine (position 429 in Drosophila melanogaster) represents the conserved aPKC phosphorylation among homologs. (TIF) [file pgen.1008890.s010.tif]

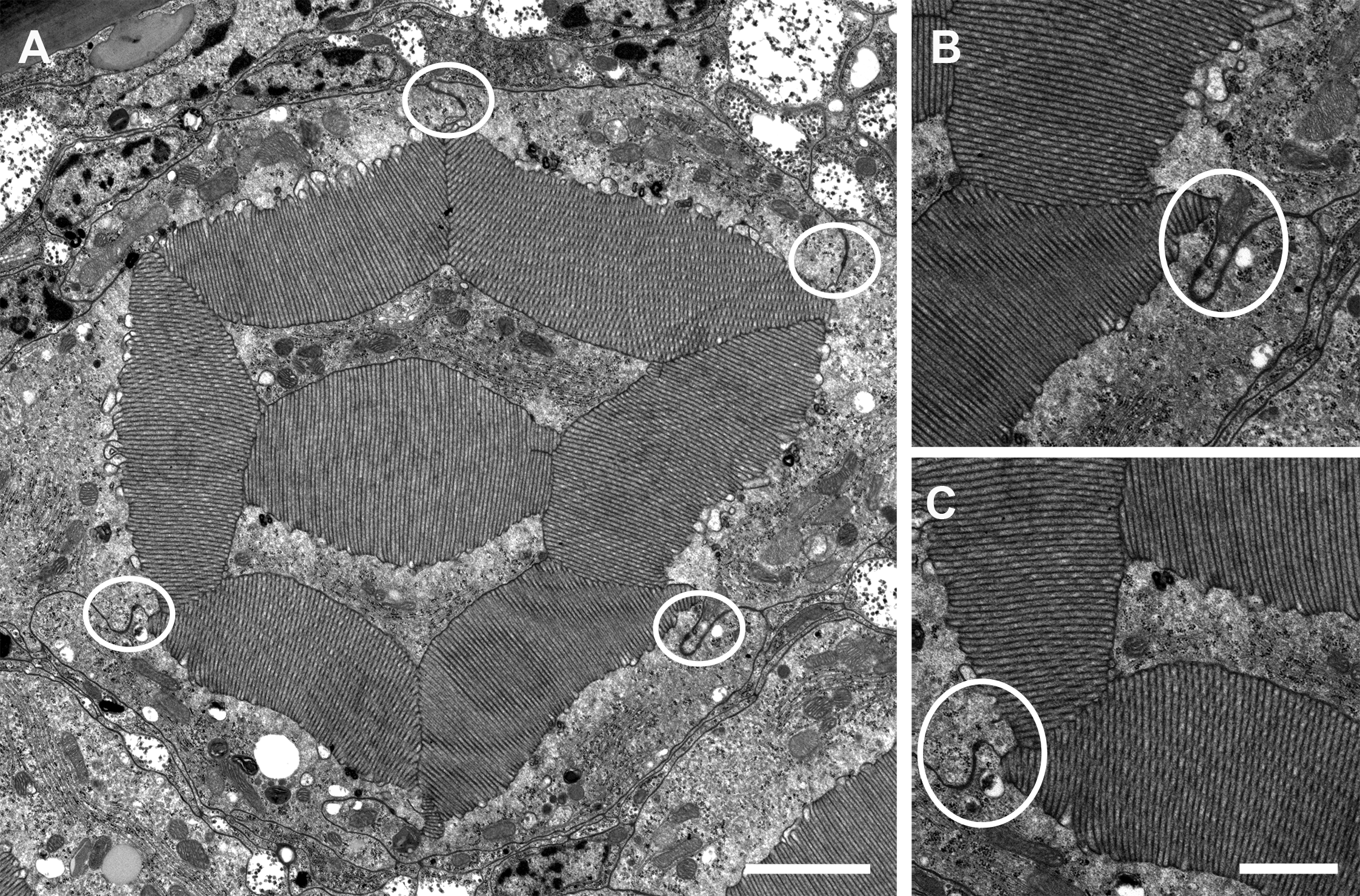

Supplement: S11 Fig — A-C Wild type ommatidium of vW adult Tribolium. B and C represent higher magnifications of regions shown in A. The adherence/septate junctions between photoreceptors are highlighted with circles. Scale bar is 10uM and 1uM. (TIF) [file pgen.1008890.s011.tif]
