# Supplementary material for: The brachyceran de novo gene PIP82, a phosphorylation target of aPKC, is essential for proper formation and maintenance of the rhabdomeric photoreceptor apical domain in Drosophila
Source: PLoS Genet. 2020 Jun 24;16(6):e1008890. doi: 10.1371/journal.pgen.1008890 (PMC7340324; doi:10.1371/journal.pgen.1008890)
Supplement: S1 File — (DOCX) [file pgen.1008890.s015.docx]

**Accession numbers of protein sequences, transcripts, and contigs:**

|  | Section | Subsection | Family | Genus | Species |  | Contig/protein sequence IDs |
| --- | --- | --- | --- | --- | --- | --- | --- |
| Orthorrhapha | - | Asiloidea | Asilidae | Dasypogon | diadema | partial | QYTT01089660 |
| Orthorrhapha | - | Asiloidea | Asilidae | Holcocephala | fusca |  | JXPE01062752/scaffold93651 |
| Orthorrhapha | - | Asiloidea | Asilidae | Proctacanthus | coquilletti |  | MNCL01000070 |
| Orthorrhapha | - | Stratiomyomorpha | Stratiomyidae | Hermetia | illucens |  | JXPW01122032_scaffold202986 |
| Eremoneura | - | Empidoidea | Dolichopodidae | Condylostylus | patibulatus | partial | BV_Cpatibulatus C30667165 |
| Eremoneura | Cyclorrhapha | Platypezoidea | Lonchopteridae | Lonchoptera | bifurcata | partial | C81360_a_6_0_l_437 |
| Eremoneura | Cyclorrhapha | Acalyptratae | Drosophilidae | Drosophila | simulans |  | XP_002106475 |
| Eremoneura | Cyclorrhapha | Acalyptratae | Drosophilidae | Drosophila | erecta |  | XP_001977431 |
| Eremoneura | Cyclorrhapha | Acalyptratae | Drosophilidae | Drosophila | yakuba |  | XP_002101133 |
| Eremoneura | Cyclorrhapha | Acalyptratae | Drosophilidae | Drosophila | eugracilis |  | XP_017086103 |
| Eremoneura | Cyclorrhapha | Acalyptratae | Drosophilidae | Drosophila | elegans |  | XP_017129791 |
| Eremoneura | Cyclorrhapha | Acalyptratae | Drosophilidae | Drosophila | rhopaloa |  | XP_016991950 |
| Eremoneura | Cyclorrhapha | Acalyptratae | Drosophilidae | Drosophila | biarmipes |  | XP_016949457 |
| Eremoneura | Cyclorrhapha | Acalyptratae | Drosophilidae | Drosophila | takahashii |  | XP_016993977 |
| Eremoneura | Cyclorrhapha | Acalyptratae | Drosophilidae | Drosophila | serrata |  | XP_020802181 |
| Eremoneura | Cyclorrhapha | Acalyptratae | Drosophilidae | Drosophila | ficusphila |  | XP_017055311 |
| Eremoneura | Cyclorrhapha | Acalyptratae | Drosophilidae | Drosophila | suzukii |  | XP_016936644 |
| Eremoneura | Cyclorrhapha | Acalyptratae | Drosophilidae | Drosophila | kikkawai |  | XP_017028648 |
| Eremoneura | Cyclorrhapha | Acalyptratae | Drosophilidae | Drosophila | ananassae |  | XP_001966994 |
| Eremoneura | Cyclorrhapha | Acalyptratae | Drosophilidae | Drosophila | bipectinata |  | XP_017094577 |
| Eremoneura | Cyclorrhapha | Acalyptratae | Drosophilidae | Drosophila | persimilis |  | XP_026845977 |
| Eremoneura | Cyclorrhapha | Acalyptratae | Drosophilidae | Drosophila | pseudoobscura |  | XP_002134634 |
| Eremoneura | Cyclorrhapha | Acalyptratae | Drosophilidae | Drosophila | hydei |  | XP_023176687 |
| Eremoneura | Cyclorrhapha | Acalyptratae | Drosophilidae | Drosophila | obscura |  | XP_022208891 |
| Eremoneura | Cyclorrhapha | Acalyptratae | Drosophilidae | Drosophila | willstonii |  | XP_023035026 |
| Eremoneura | Cyclorrhapha | Acalyptratae | Drosophilidae | Drosophila | arizonae |  | XP_017870878 |
| Eremoneura | Cyclorrhapha | Acalyptratae | Drosophilidae | Drosophila | virilis |  | XP_002055383 |
| Eremoneura | Cyclorrhapha | Acalyptratae | Drosophilidae | Drosophila | mojavensis |  | XP_002010583 |
| Eremoneura | Cyclorrhapha | Acalyptratae | Drosophilidae | Drosophila | navojoa |  | XP_017964184 |
| Eremoneura | Cyclorrhapha | Acalyptratae | Drosophilidae | Drosophila | grimshawi |  | XP_001992749 |
| Eremoneura | Cyclorrhapha | Acalyptratae | Ephydridae | Cirrula | hians |  | JXOS01136913.1_scaffold342677 |
| Eremoneura | Cyclorrhapha | Acalyptratae | Ephydridae | Ephydra | gracilis |  | JXPQ01036598.1_scaffold45734 |
| Eremoneura | Cyclorrhapha | Acalyptratae | Tephritidae | Bactrocera | latifrons |  | XP_018782934 |
| Eremoneura | Cyclorrhapha | Acalyptratae | Tephritidae | Bactrocera | oleae |  | LGAM01000060.1 |
| Eremoneura | Cyclorrhapha | Acalyptratae | Tephritidae | Bactrocera | dorsalis |  | XP_011200418 |
| Eremoneura | Cyclorrhapha | Acalyptratae | Tephritidae | Ceratitis | capitata |  | XP_004520033 |
| Eremoneura | Cyclorrhapha | Acalyptratae | Tephritidae | Rhagoletis | zephyria |  | XP_017474957 |
| Eremoneura | Cyclorrhapha | Acalyptratae | Tephritidae | Zeugodacus | cucurbitae |  | XP_011185090 |
| Eremoneura | Cyclorrhapha | Calyptratae | Calliphoridae | Lucilia | cuprina |  | XP_023296141 |
| Eremoneura | Cyclorrhapha | Calyptratae | Muscidae | Musca | domestica |  | XP_005188505 |
| Eremoneura | Cyclorrhapha | Calyptratae | Muscidae | Stomoxys | calcitrans |  | XP_013105203 |
| Eremoneura | Cyclorrhapha | Calyptratae | Rhinophoridae | Paykullia | maculata |  | NDXZ01020741.1 scaffold42832 |
| Eremoneura | - | Empidoidea | Dolichopodidae | Heteropsilopus | ingenuus |  | s11934_L_39310_0_a_35_0_l_2480 |
| Eremoneura | Cyclorrhapha | Platypezoidea | Phoridae | Megaselia | abdita |  | JXPG01001308.1_scaffold1565 |
| Eremoneura | Cyclorrhapha | Platypezoidea | Platypezidae | Platypeza | anthrax |  | s4671_L_6202_0_a_7_7_l_2037 |
| Eremoneura | Cyclorrhapha | Syrphoidea | Syrphidae | Episyrphus | balteatus | partial | s8567_L_28372_0_a_29_2_l_3010 |
| Eremoneura | Cyclorrhapha | Syrphoidea | Syrphidae | Eristalis | pertinax |  | s7865_L_21645_0_a_25_9_l_3667 |
| Eremoneura | Cyclorrhapha | Syrphoidea | Syrphidae | Merodon | equestris |  | C101934_a_40_0_l_3795 |

**PIP82 homolog sequences identified:**

**Species name abbreviation code:**

First letter = first letter of genus name

Letters 2-4 = first three letters of species name

>Dmel_PIP82

MSHQEQQFQHYPHHQHHHHHHHHHIHQVQSETQLEQRSSDLEPNRSRNTDRIGSSMDFRNLCQRIDVDGLKAKLPQLKLPKSLPKLRGRKIFRSSKSGSNAAGGGTAGGSAKDGAGAAQQTHLQVAGQSQQFINRTPQRISTISSLMYEGEQEEIRANLGQSQPGTYRSAGSLDDDYYAPGSGDRASRPISPIKIPVVGALDDSSPSENGNVRTTFTQRLQRGYKSLSELRIKHIFAKQTTVRRDNIEVDRYVEQYERELKSEKLARERRDREIAENYDIKIKTLAGTRQNTFDDDDVEHEQFERGKISHETDESGMEATPPLPSRRKPGIAATRFAKVRKPPLEMEEEQQQAAAEESPQANPPPPPPPRPSSYKQLLINKLPHLPSLPNLPQFSRTKEETTKTAENADENNASRKLSIRQNIKRLRKSIKRPSKIKSKAAAPVPDSDEEEATPDGQKTKDAPTRSSTANLRARLSRFASTEQLQQRWRKSFKVAKEPEELETKAEGSATGGASGVLGGLLVGSQLEKTLAKLNEKVHQLKFFQRNANNQNATTSKQPKPNTVGHEPIEIDDDELEATYHRSDSLEAENGNGNENDDSGEDISAEEAFGQIQEEDNEEDHSQDQTKRGQSSVSGIATAHAARQMAKLAEIQAASKSGAAAWSSESLEEIADEDYPRVLIHQEHSDAYESTLIIAVASKGSSMSPVVRSSGLKSSPAAGPKTSPHPEIRISASGPQKSMSYSPGNPRGEPVTKRSPSPEFKTPAGGNKIVPKSETSAWLPNEQIIAGFKEQTSWPAPALYKPKSIDIFEASAGGSAAFADFDEALRNAPVLRISAGSSIDTSGEEADDSCSRVTRIRVQSPQIGNSRESLMAQEEEDKEAERDSEEEEEERDPSERPPSESPPPPPLPQRRPPTKRPATPPIYDAVPPPLPVSKPPPPPSVETIPSVASLPSPAPVTRSMAQRSASMSRPAKPLVKTSSLRLTYNEQVRPGDVGKVNKLISRFEGGRPRLCPRRMHSEEYERCAQPEDEEPEMEQILELQIIERRAVDSVTPTNRAVVIPQITVNNNNNNERQLEQSDQSDQSAHQEITDTRKTKSMELALDRQNSNCSRSEYGSPLSFPSSRRSSTPTNLNANSNSNPNPSTNPNQNPSQILQHQRRSRRSMTRDDDNFYSFDSDEENSYYSISPSGSSRYVVEI

>Dsim_XP_002106475

MSHQQQQFQHYHHHQHHIHQVQSESQLEQRSSDLEPNRSRNTDRIGSSMDFRNLCQRIDVDGLKAKLPQLKLPKSLPKLR

GRKIFRSSKSGSNAAGGTAGGSSKDGAGAAQQSHLQVAGQSQQFINRTPQRISTISSLMYEGEQEEIRANLGRSQPGTYR

SAGSLDDDYYAPGSGDRASRPISPIKIPVVGADDSSPSGNGNARTTLTQRLQRGYKSLSELRIKHIFAKQTTVRRDNIEV

DRYVEQYERELKSEKLARERRDREIAENYDIKIKTLAGTRQNTFDDDDVEHEQFERGKMSHETDESGMEATPPLPSRRKP

GIAATRFAKVRKPPLEMEEQQQATGDGAAAEESPRANPPPPRPSSYKQLLNKLPHLPSLPNLPQFSRSKEEPTKTGENAD

ENNDSSKMSIRQNIKRLRKSIKRPSKIKPKAAAPAPDSDEEDQGDEVTPEGQKTKDAPTRSSTANLRARLSRFASTEQLQ

QRWRKSFKGGKEPKELETKAEGSATGGAAAAAAGASGVLGGLLVGSQLEKTLAKLNEKVHQLKFFQRNANNQNATTSQQP

KPNTVGHEPVEIDDDELEATYHRSDSLEDENRNENDDSGEDISAEEAFGQIQEEDDEEDHSQDQAKRGHSPVSGVATAHA

ARQMAKLAEIQAASKSGAAAWSSESLEEIADEDYPRVLIHQEHSDAYESTLIIAVASKGSSVSPVIRSSGLKSSPAAGPK

TSPHPEIRISASGPQSMSYSPRNPSGEPVTKRSPSPEFKTPAGGTKIPPSEASAWLPNEQIIAGFKEQTSWPAPALYKPK

SIDIFEASAGGSAAFADFDEALRNAPVLRISAGSSIDTSVEEADDSCSRVTRIRVQSPQIGNSRESLMAQEEEDEEAERD

SEEEQADEERDPSERPPSESPPPPPLPQRRPPPKRPATPPIYDAVPPPLPVSKPPPPPTAETIPSVAPVPSPAPVTRAIP

QRSASMSRPAKPLVKTSSLRLTYNEQVRPGDVGKVNKLISRFEGGRPRLCPRRMHSEEYERSAQAEDEEPEMEQILELQI

IERRAVDSVTPTNRAVVIPQITVNNNNERQLEQLDQSDHQEITDARKKKSMELALDRQNSNCSRSEYGSPLSFPSSRRSS

TPTNLNANTNPNPNPSTNPSQNPSQILQHQRRSRRSMTRDDDNFYSFDSDEENSYYSISPSGSSRYVVEI

>Dere_XP_001977431

MSHQQQQFQHYHHHHIHQVQSESQLEPRSSDLEANRSRNTDRIGSSMDFRNLCQRIDVDSLKAKLPQLKLPKSLPKLRGR

KIFRSSKSGSNAAGGTAGGSSKDGAGATAPQQSHLQLAAQSQQFINRTPQRISTISSLMYEGEQEEIRANLGRSQPGTYR

SAGSLDDDYYAPGSGERASRPISPIKIPVVADDSSPSGNAHTTLTQRLQRGYKSLSELRIKHIFAKQTTVRRDNIEVDRY

VEQYERELKSEKLARERRDREIAENYDIKIKTLAGTRQNTFDDDYVEHEQFERGKMSHETDESGMEATPPLPSRRKPGIA

ATRFAKVRKPPLDMEEQQQAAGYGEAVDESPRANPTPPRPSSYKQLLNKLPHLPSLPNLPQFSKSKEEATKTGENTDENN

DSSKLSIRQNIKRLRKSIKRPSKIKPKAAAPDSDEEDQGEEAAPEGQKTKDAPARSSTANLRARLSRFASTEQLQQRWRK

SFKGAKETNELETKPEGSATGAAAAGASGVLGGLLVGSQLEKTLAKLNEKVHQLKFFQRNASNENATTSQQPKPNTVGHE

PIEIDDDELEATYHRSDSLEDEKGNNDDSGEDISAEEAFGQIQEEDEEDHSPDQAKRGHSAVSGVATTHAARQMAKLAEI

QAASKSGAAAWSSESLEEIADEDYPRVLIHQEHSDAYESTLIIAVASKGSSVSPVIRSSGLKSSPAAGPKTSPHPEIRIS

ASGPQSLSYSPGNSSGVPVTKRSPSPEFKTPAGGSKIPPTETSVWLPNEQIIAGFKEQASWPAPALYKPKSIDIFEASTG

GSAAFADFDEALRNAPVLRISAGSSIDTSGEEADDSSSRVTRIRVQSPQIGNSRESLMAQEEEDEEAERDSEEEPTEDLR

DSSERPPSESPPPPPLPQRRPPPKRPATPPIYDAVPPPLPVSKPPPPPLTETIPSVAPLPSPAPVSRCIPQRSASMSRPA

KPLVKTSSLRLTYNEQVRPGDVGKVNKLISRFEGGRPRLCPRRMHSEEYERSAQAEDEEPEMEQILELQITERRVVDSVT

PTNRAVVIPQITVNNNNEPQSNQSDQQEIADSRKRKSMELALDRQNSNCSRSEYGSPLSFPSSRRSSTPTNLNANTNPNP

NPSSNPNQNPTQILQHQRRSRRSMTRDDDNFYSFDSDEENSYYSISPSGSSRYVVEI

>Dyak_XP_002101133

MSHQQFQHHHHHHHHQHIHQVQSESQLEQRSSDLEPNRSRNTDRLGSSMDFRNLCQRIDVDSLKAKLPQLKLPKSLPKLR

GRKIFRSSKSGSNAAGGTAEGGSLKDGPGGGAVQQSHLQVAAHSQQFINRTPQRISTISSLMYEGEQEEMRANLGRTQPG

TYRSAGSLDDDYYAQAGSGGERASRPISPIRMPVAGVDCVDVSSSSPPGGSARTTLTQRLQRGYKSLSELRIKHIFAKQT

TVRRDNIEVDRYVEQYERELKSEKLARERRDREIAENYDIKIKTLAGTRQNTFDDDDVEHEQFERGKMSHETDESGMEAT

PPLPSRRKPGIAATRFAKVRKPPLEMEEQQQAVGDVEESPRANPPQPPRPSSYKQLLNKLPHLPSLPNLSQFSRSKAETT

KTAENADANNASSSKLSIRQNIKRLRKSIKRPSRVKSKAAAPDSDDEDQVEEAAPEKTKDGAARSSTANLRARLSRFAST

EQLQQRWRKSFQGAKEPKELETKAEGTGANGATGAAAAGATGVLGGLLVGSQLEKTLAKLNEKVHQLKFFQRNASNQNAT

RCQQPKPNTVGHEPIEIDDDELEATYHRSDSLEDENGNNDDSGEDISAEEAFGQIQEEDEEDHSPAQAKRNNVSVSGVAS

AHAARQMAKLAEIQAASKSGAAAWSSESLEEIADEDYPRVLIHQDHSDAYESTLIIAVASKGSSLSPGIRSSGLKSSSAA

GAKTSPHPEIRISTSGPQRLSYSPGNPSAPPDSVTKRSPSPEFKTPAGGSKIAPSEASAWLPNEQIIAGFKEQTSWPAPA

LYKPKSIDIFEASAGGSAAFADFDEALRNAPVLRISAGSSIDTSGEEADDSSSRVTRIRVQSPQIGNSRESLMAQEEDDE

EADRDSEEEQAQKEEEEEEEDGERDASERPPSESPPPPPLPQRRPPQKRPATPPIYDAVPPPLPVTRPPPPPPVEAIPLV

APSLPSPAPISRGIPQRSASMSRPAKPLVKTSSLRLTYNEQVRPGDVGKVNKLISRFEGGRPRLCPRRMHSEEYERSAQV

EDEEPEMEQILELHISERRVIDSVTPTNRAVVIPQITVNNNNERPSDEADPTERQAIAGDSKRRRKQSMELALDRQNSNC

SRSEYGSPLSFPSSRRSSTPTNLNANPNPNPNPNANSNPSTITNPNPNTNQNPTLILQHQRRSRRSMTRDDDNFYSFDSD

EENSYYSISPSGSSRYVVEI

>Deug_XP_017086103

MSHHQQQQQQQFHHYHHHIHQVQSESQLEQRNSDLEHNSNRNTNRIGSSMDFKNLCQRIDVDGLKAKLPQLKLPKSLPKL

RGRKIFRSSKSGADGAAGGSSKDGAGGGQQQQTHLQVAAQSQQFINRTPQRISTISSLMYEGEQEEIRASLGRSQPGTYR

SAGSLDDDYYAPGSGERASRPISPIKIPVGGTDDTGNSRTTLTQRLQRGYKSLSELRIKHIFAKQTTVRKDNIEVDRYVE

QYERELKSEKRARERRDREIAENYDIKIKTLAGTRQNTFDDDEVEHEQFEKGKISHETDESGMEATPPLPSRRKPGIAAT

RFAKVRKPPLEMEEQQQQTGDEDSKESPPRKQTPPRPSSYKQLLNKLPHLPSLPSLPQLTRSKEETPKTEENAEANNPSS

KLGIRQNIKRLRKSIKRPAKIKTKVTAPDSDEDEDVTEGDRPKEPPVKSSSANLRARLSRFASTEQLQQRWRKSFKGSRE

TKDGDPKQGDNVEPSSGAAASGILGGLLLGSQLEKTLTKLNEKMHQIKFFQRHSNSQNSASSQQPKPNTVGHEPVEIDDE

ELAATYHRTDSLENENGNDDSGEDISAEEAFGQIQQEDQDQDQTKSSNSSVSGTSTTHAARQMAKLAEIQAASKSGAAAW

SSESLEEIADEDYPRVLIHQEHSDAYESTLIIAVASKGGSVSPALRTSGYSPCLGLKASPATGIRTSPYPEIKISSSGPQ

SLSYSPTTSYPDQTTTNRSPSPEFKTPAGGIKVSPSEASVWLPNEQIIAGFKEQTSWPAPALYKPKSIDIFEASAGGSAA

FADFDEALRNAPVLRISAGSSIDTSGEEADDSSSRVTRIRVQSPQIGNSRESLMAQEEEDEEEVEVDRDSEEDRDPSERP

PSESPPPPPLPQRRPPPKRPATPPIYDAVPPPLPISKPPPPPPAETPTPFVTPVPVPVPVPSPVPLSRGGIPQRSASMSR

PAKPLVKTSSLRLTYNEQVRPGDVGKVNKLISRFEGGRPRLCPRRMHSEELERCGHGDEEEPEMEQILELQITEKRVIAD

SVTPTNRAPVVIPQITLNNNNERQSDSDQDEIAKSRKKQSMDLALDRQNSNCSRSEYGSPLSFPSSRRSSTPTNLNANAN

PIPSLITNPNQNSVQSLQQQRRSRRSMTRDDDNFYSFDSDEENSYYSISPSGSSRYVVEI

>Dele_XP_017129791

MSHQQQHQFHHYHHHIHQVQSESELERRNSDSEQNPNRNTDRIGSTMDFRNLCQRIDVDSLRAKLPQLKLPKSLPKLRGR

KIFRSSKRGADGSGGTAAGLSKDGAQPQQSQLQLAPQSQQFINRTPQRISTISSLIYEGEQDEIRANLGRSQPGTYRSAG

SLDDDYYAPGSGERASRPISPIKIPAGGADTAASGNSRPTLSQRLQRGYKSLSELRLKHIFAKQTTVRRDNIEVDRYVEQ

YERELKSEKLARERRDREIAENYDIKIKTLAGTRQNTFEDDDELEHEQFEKGKICHETDESGMEATPPLPSRRKPGIAAT

RFAKVRKPPLEMEGQQAGEEDQPPPRGRPSSYKQLLNKLPHLPSLPNLAQLSRGKEEASKSGENAEANNPGSKLGIRQNI

KRLRKSIKRPAKIKPKATPDSEEEEEPEGVAEGNKPKDPLGKGSVMNLRARLSRFASTEQLQQRWRKSFKGTKEHKEGES

SAEPTAASGVLGGLLLGSQLEKTLAKLNEKVHQLKFFQRHASQNPETSKQPKPNTVGHEPVEIDDDEELAATYHRSDSLE

AEHDEANDDSGEDISAEEAFGQMQPDKGASVSGNASAHAARQMAKLAEIQAASKSGAAAWSSESLEEMADEDYPRVLIHQ

EHSDAYESTLIIAVASKRSSLSPGIRSSPTATGIKGSPYPEITISASPSPGQTPAAGIKVSPSEANVWLPNEQIIAGFKE

QATSWPAPTLYKPKSIDIFEAAAAGGSAAFADFDEALRNAPVLRISAGSSIDTSGEEADDSSSRITRIRVQSPQIGNSRE

SLMAQEEEDEEEVDLPEEDEDEEEDGSENGEDQDSDRDASERPPSESPPPPPLPQRRPPPKRPATPPIYDAVPPPLPVTK

PPETFVPAPPAVISRAVPPRSASMSRPAKPLVKTSSLRLTYNEQVRPGDVGKVNKLISRFEGGRPRLCPRRMHSEEYDRS

TRTDDDDDEVEPEMEQILELQITERRAMDSVTPTNRAVVIPQITLNNNNNNNNNNNNNNNDNNNSEKQLDDELDEIAKSR

KKKSTELALDRQNSNCSRSEYGSPLSFPSSRRSSTPTNLNFNSNSNPISNLSSNLNQNPSQVLQQQRRSRRSMTRDDDNF

YSFDSDEENSYYSISPSGSSRYVVEI

>Drho_XP_016991950

MNYQQQQQHQFHHYHHHIHQVQSESQLERRNSVTSEQNPNRNTDRIGSTMDFRNLCQRIDVENLRSKLPQLKLPKSLPKL

RGRKIFRSSKSGADAAGGAAAGSTKDGGQPQQSHLQVAPQSQQFINRTPQRISTISSLMYEGDHEEGRANLGRSQPGTYR

SAGSLDDDYYAPGSGERASRPISPIKIPAGGADTPPTGNSRTTLTQRLQKGYKSLSELRLKHIFAKQTTVRKDNIEVDRY

VEQYERELKSEKLARERRDREIAENYDIKIKTLVGTRQNTFDDDEMEHEQFEKGKISHETDESGMEATPPLPSRRKPGIA

ATRFAKVRKPPLEMEEQAAAEVESSANQPPPPRRPSSYKQLLNKLPHLPSLPNLPQLSRGKEEASKTDENAEENNPSSKL

GIRQNIKRLRKSIKRPAKIKPKATTDSDEEEDGEGEGDKPKDQPGKSSSMNLRARLSRFASTEQLQQRWRKSFKSSKASK

EGEGTPGANVEPTATSGVLGGLLLGSQLEKTLAKLNEKVHQLKFFQRHSSQNPEMSQPKPNTVGHEPVEIDDDEELAATY

HRSDSLENENDQNDDSGEDISAEEAFGQIQPEDPDQARKGPSLSGTAATHAARQMAKLAEIQAASKSGAAAWSSESLEEI

ADEDYPRVLIHQEHSDAYESTLIIAVASKGSVSPGIRSVGYSPRPGVKSFPATGPRGSPYPEITISSSGPRSSSCSPSSG

YPGDSGAQPTAKRSPSPEFKTPAAGIKVPPAEANAWLPNEQIIAGFKEQASWPAPTLYKPKSIDIFEASAGGFAAFADFD

EALRNAPVLRISAGSSIDTTTSGEEADDSSSRVTRIRVQSPQIGNSRESLMAQEEEDEEDAELAEEQEQEQDRYSERDAS

ERPPSESPPPPPLPQRRPPPKRPATPPIYDAVPPPLPISKPPPPPPETVATAVAPAAPASRGIPQRSASMSRPAKPLVKT

SSLRLTYNEQVRPGDVGKVNKLISRFEGGRPRLCPRRMHSEEYERSGQEEDEVEPEMEQILELQITERQTMDSVTPTNRA

VVIPQITLNNNNARQTTDEQDEIARSRKKKSMELALDRQNSNCSRSEYGSPLSFPSSRRSSTPTNINSNSNPIPNHSSNL

NQNPVQVLQHQRRSRRSMTRDDDNFYSFDSDEENSYYSISPSGSSRYVVEI

>Dbim_XP_016949457

MSHQQQQFHHHHHNFHPGQLESQLERRNSPDLEQNSNRNTDRIGSSMDFRNLCQRLDVDSLRAKLPQLKLPKSLPKLRGR

KIFRSSKSGPDAPGRSSKDAGGGGAPQQSHLQVAGAQSQQFINRTPQRISTISSLMYEGEQEEIRVGQLGRSQPGTYRSA

GSLDDDYYAPGSGDRASRPISPIKIADDSPPPGNTGTSTLTQRLQRGYKSLSELRLKHIFAKQTTVRRDNIEVDRYVEQY

ERELKSEKLARERRDREIAENYDIKIRTLVATRQNTFDEDEVEHEQFEKGKSGNHETDESGMETTPPLPSRRKPGIAATR

FAKVRKPPLEMEEAGKEHEDQEAPPARQRPSSYKQLLTKLPHHLPSLPNLSQFSRSKEEASSKTAESAGARESNPGSMLG

IKQNIKRLRKSIKRPAKTKTKATRAAGDSDEEKDGELEEQEVAPKHPPGRSSTSNLRARLSRFASTEQLQQRWRKSFKER

APKEPKEGANVDPTGAATGVLGGLLQGSQLEKTLAKLNEKMHQLKFFQQHSNANENPVKPEREPKPNTVGHEPVELDDDE

ELEATYHRSDSLDDEEEQNDDSGEDISAEEAFGQIQEEHKEEDARKRASLSGTATTHAARQMAKLAKIQAASKGGATAWS

SESLEEMAEDDYPRVLIHQEHSDTYESTLIIAMSSKAGSVSPSPYPEIKISSSCSPTPRSPSPEFKTPAGGSNVWLPNEQ

IIASFKEHIPSPSWPAPALYKPKSIDIFEASAGGSSAAFADFDEALRNAPVLRISAGSSIDTSGEEADDSSSRVTRIRVQ

SPQIGNSRESLMAQEEEEDRYSDEEEEQEEEDEDEERNASERPPSESPPPPPLPQRRPPPKRPATPPIYDAVPPPLPISK

PPTAPPAETAPLGAPVPAPAPAPAPAPDPTPISRGIPQRSASMSRPAKPLVKTSSLRLTYNEQVRPGDVGKVNKLISRFE

GGRPRLCPRRMHSEGEESQEDVPEMGRILELQITEKRAVDSVTPTNRAVVVIPQITLNNNNNNNNNNESDKDDELAQSGR

KRKSMELALDRQNSNCSRSEYGSPLSFPSSRRSSTPTNTTTSTPNLGSNPNQNLQHQRRSRRSMTRDDDNFYSFDSDEEN

SYYSISPSGSSRYVVEI

>Dtak_XP_016993977

MSHQHQQQQFHHHHQHIHQVQSESQLERRSSSDLEPNRNRHTDRIGSSMDFRNLCQRIDVESLRAKLPQLKLPKSLPKLR

GRKIFRSSRSGTDTAGEGGAGAVAAGRSSKDAGATAHQSQHHLQVAAPQSQQFINRTPQRISTISSLMYEGEQLEEQLGR

SQPGTYRSAGSLDDDYYAPGSGERASRPISPIKIPASPQGNTNAPITLTQRLQRGYKSLSELRIRHIFARQTTVRRDQIE

VDRYVEQYERELATEKLARERRDREIAENYDIQIKTLAGSRQNTFDDDVELEHRQLEQRSKLAQSHETDESGMEATPPSL

PSRRKPGIAATRFAKVRKPPLEMEEQSAEREETPPRPSSYRQFVSKLPSLPNLPQFSRSKEDPARQAENEGALENQSNSK

FGLRQNIKRLRKSIKRPAKIKGKASANPRDSDEEENEGEEQEKEPPGKISSSGNLRARLSRFASTEQLQQRWRKSFKERA

PKRDEAKEDATKPGGILGGLLLGSQLEKTLAKLNERMHQLKFFQRNSNENPAKTREPPKPNTVGHEPVEIDDDEELAATY

HRSDSLEHEDPDGQDDSGEDISAEEAFGQMQPEEEEQSSSVSGNATTHAARQMAKLAEIQAASKSGAAAWSSESLEEIAD

EDYPRVLIHQEHSDAYESTLIIAVASAKGSVIKTSPYPEIKISKPGPQRLSCSPPEKSKSLSRSPSPEFKTPKCDPNSAW

LPNERIIAGFKEQANWPAPALYKPKSIDIFEGSSSAFADFDEALRNAPVLRISAGSSIDTSGEEADDSSSRVTRIRVQSP

QIGNSRESLMAQEEEEDCYSEEEDEDRDASERPPSESPPPPPLPQRRPPPKRPATPPIYDAVPPPLPISKPPPPPPPSAE

VAPPAEAPPVPSPTPIARTMPQRSASMSRPAKPLVKTSSLRLTYREQVRPGDVGKVNKLISRFEGGRPRLCPRRMHSEGE

EEVDLPEMEKILELQMSEKRAVDSVTPTNRGAVLIPQITLNNNNQDDDRDHDDEIARSGKKKTMELALDRQNSNCSRSEY

GSPLSFPSSRRSSTPTNISIPNLSSNPIPNQNLNQNQNPNPNANQILQHQRRSRRSMTRDDDNFYSFDSDEENSYYSISP

SGSSRYVVEI

>Dser_XP_020802181

MSHQQQQFLHYHHHQVHQQVQSEMQMDRRSSDLDGKKSGDPQNRNRNTERGAGGVSMDLRNLCQRIDVDSLRAKLPSLPQ

LKLPKSLPKLRGRKIFRSSKSGVASGENGPGAGAVAGGSSKDGGQPQQMLQVAAQSQQFINRTPQRISTISSLMYEADHE

EVRHSQQPGGTYRSAGSLDDDYYAPSTGDRASRPISPIKIPTAGGAEASPSGTGHATLTQRLQRGYKSLSELRLKHIFAK

QTTVRRDNIEVDRYVEQYERELKSEKRAQARRDREIAENYDIKVQTLATTRQNTFEDDEVEREQFEKSKISHETDESGME

ATPPLPTRRKPGIAATRFAKVRKPPLEMEEQSVGKDEENQQETPPPPPPPRPSSYKQQLLNKLPNLPSLPNLSQLSKTKE

ERSNNQENTSASKSGLRQNIKRLRKSIKRPAKIKPKAKADSDEEEETAETDRDKDKSSDPPAKSSSANLRARISRFASTE

QLQQRWRKSFKSSSSKVNREATEKVDNTDSTPGATATTGVLGGLLLGSQLEKTLAKLNEKMHQLKFFQRYSNRNQEPMQP

KPNTVGHEPVEIDDDEELAATYHHSDSDEDEDEDADGDSGEDISAEEAFGKIQEEEDRYQNSQDNSRRGSSSLSGTAPAH

AARQMAKLAEIQAASKSGAAAWSSESLEEIADEDYPRVLIHQEHSDAFESTLIIAVASKGSVSVSPGVRNTGSQTYPVVK

YSSSGFEQTATGGAPSRSPGVKDSPSPEFKTPAAQTPDASAWLPNEQIIAGFKDQTNASWPAPALYKPKSIDIFEAASGG

SAAFADFDEALRNAPVLRISAGSSCDTSGEEADDSTSRVTKIRVQSPQIGNSRESLMAQEEEDEEDAEADADVDMELEME

GVLDLDLDLERDSSERPPSASPPPPPLPQRRPPPPKIPATPPIYDAVPPPLPVSKPPIPDKAPTTAPGIKSPPPTVTKEI

PPVSKENPLLGRGIPQRSASMSRPAKPLVKTSSLRLTYNEQVRPGDVGKVNKLISRFEGGRPRLCPRRMHSEEYERGRED

DEAEEEDAEPLLELKNFQKRAVESVTPTNRGVIIPQITVNNNNNNSNEKSTEEEGTEEDDITKSRKKKRESLELSLDRQN

SNCSRSEYGSPLSFPSSRRSSTPTNLSSNPNPHPILNPNANPIQNPVQILQQQRRSRRSMTRDDDNFYSFDSDEENSYYS

ISPSGSSRYVVEI

>Dfic_XP_017055311

MMMTNHQQHHQQQQQHQQFHHYHHHQIHQVHSESQLERRNSDLEQNRSRNTTDRIGSAMDFRNLCQRIDVDSLRAKLPQL

KLPKSLPKLRGRKIFRSSKSDEHPAGGAATGSSKNGGQLQQQQQSHLELPGQSQQFINRTPQRISTISSLMYEGEQEEMR

ANQGQLQPGTGTYRSAGSLDDDYYAPGGSGERASRPISPIKIPAGGADGSPPTGGGGGVRSSITQRLQRGYKSLSELRLK

HIFAKQTTVRRDNIEVDRYVEQYERELKSEKLARERRDREIAENYDIKIKTLAGTRQNTFEEDEVEHEQFERGKLNHETD

ESGMEATPPLPNRRKPGIAATRFAKVRKPPLEMEEQSGELEEDQPQAPPRPSSYKQLLNKLPHLPSLPSLPQLSREKKEE

PATKAEENAEEINPSSSSKYGIRQNIKRLRKSIKRPAKLKSKASTAADSDEEEDPVDKPKDQLPRKTSSINLRARLSRFA

STEQLQQRWRKSFKGSTKETREPEAHGATEEPSNAGGVLGGLLLGSQLEKTLAKLNDKVHQLKFFQRHSGPNPEISQPKP

NTVGHEPVEIDDDGELPETYHRSDSPESGENGRDNEEDSGEDVSAEEAFGQIQRETQDPNRERRKPSVSENKSTHAARQM

AKLAEIQAASKSGAAAWSSESLEEMADEDYPRVLIHQEHSDAYESTLIIAVAAGKGSLSPRLPKGSPAAAGAARSSPYPE

ITISSSLEVVSPAVAVSSNPAKRSPSPEFQTPAGGIKVSASEASIWLPNEQIIAGFREQQASWPAPSLYKPKSIDIFEAS

AGGSAAFADFDEALRNAPVLRISAGSSIDTSGEEADDSSSRVTRIRVQSPQIGNSRESLMAQEEEDEEDAELVADRNGNE

DEEADEDEEEEEDSSERDASERPPSESPPPPPLPQRRPPPKRPATPPPIYDAVPPPLPISKPPNAAPPSPNPAPPASPAV

GTAAAAPAAAVAIPPVVSRGVPQRSASMSRPAKPLVKTSSLRLTYNEQVRPGDVGKVNKLISRFEGGRPRICPRRMHSEE

YERSGHEDEPEMEEILELQLTEKRIVDSVTPTNRAVVIPQITLNNNNNSNEDQDALLLAQSRAKKKSMELALDRHNSNCS

RSEYGSPLSFPSSRRSSTPTNLSSQSSSIANPNPNPNPNQNSVQSLQQQQQQRRSRRSMTRDDDNFYSFDSDEENSYYSI

SPSGSSRYVVEI

>Dsuz_XP_016936644

MSHQQQQFHHYHHHIHQVQSESQLERRNSPDLEQNRTRNTDRIGSSMDFRNLCQRIDVDSLRAKLPQLKLPKSLPKLRGR

KIFRSSKSGTEAAGRSSKDAGGGGGAPQQSHLQVAGTQSQQFINRTPQRISTISSLMYEGEQEEIRVGQLERSQPGTYRS

AGSLDDDYYAPGSADRASRPISPIKIPDDPPPTGNTGTSTLTQRLQRGYKSLSELRLKHIFAKQTTVRRDNIEVDRYVEQ

YERELKSEKLARERRDREIAENYDIKIRTLVATRQNTFDEDEVEHEQFEKGKSANHETDESGMEATPPLPSRRKPGIAAT

RFAKVRKPPLEMEEAGKELEVQEPPPVPQRPSSYKQLLNKLPHHLPSLPNLSQFSRSKEEASFKTEENAGAGETNPGSKL

GIRQNIKRLRKSIKRPAKNKTKATKAAVDSDEERDGEEEEQETPKDAPGRSSTSNLRARLSRFASTEQLQQRWRKSFKER

VPKEPKEGTNVDQTGAATGAATGVLGGLLLGSQLEKTLAKLNEKMHQLKFFQRHSNANENPVKSERERKPNTVGHEPVEL

DDDEELPTTYHRSDSLEDEDEQNDDSGEDISAEEAFGQIQEEHQEEHERGKRESLSGAAATHAARQMAKLAEIQAASKGG

ATAWSSESLEEMAEEDYPRVLIHQEHSDAYESTLIIAMSSKASSVSPYPGIKASSSIGRKTSPYPEIKISSSYSPTARSP

SPEFKTPAGGSNVWLPNDRIIAGFKEQTSSSWPAPALYKPKSIDIFEASAGGSSVAFADFDEALRNAPVLRISAGSSIDT

SGEEADDSSSRVTRIREEEEEEEVDRYSDEEESEEEEEERDASERPPSESPPPPPLPQRRPPPKRPATPPIYDAVPPPLP

ISKPPPAPQALTAPLVAPAPAPAPAPAPDPTPTPISRGIPQRSASMSRPAKPLVKTSSLRLTYNEQVRPGDVGKVNKLIS

RFEGGRPRLCPRRMHSEGEESQEDVPEMEQILELQITEKRAVDSVTPTNRAVVVIPQITLNNNNNNDNESDRDDEVVLSG

RKKKSNELALDRQNSNCSRSEYGSPLSFPSSRRSSTPTNTTTSMPNLGSDSNQNPNPNQILQHQRRSRRSMTRDDDNFYS

FDSDEENSYYSISPSGSSRYVVEI

>Dkik_XP_017028648

MSHQPQQQQFLHYHHHVHHQVQSETQMDRRNSDLDLERNRAERDHQNRNRTTDRGGGGGVSMDLRNLCQRIDVDSLRAKL

PSLPQLKLPKSLPKLRGRKIFRSSKSGATAGEAGATTGVAGSSKDGGQPQQLLQVAAQSQQFINRTPQRISTISSLMYEG

DHEEMRQSQQQQPGTYRSAGSLDDDYYAPSNGDRASRPISPIKIPTAGGADGSPSGTGHATLTQRLQRGYRSLSELRLKH

IFAKQITVRRDNIEVDRYVEQYERELKSEKRAQARRDREIAENYDIKVQTLVATRQNTFEDDEVEHEQFEKSKISHETDE

SGMEATPPLPTRRKPGIAATRFAKVRKPPLEMEEQMSSKEEENQQETPPPPPPQRPSSYKQQLLNKLPNLPSLPNLSQLS

KSKEERSNGQENTSGSKSGLRQNIKRLRKSIKRPAKVKPKAKADSDEEEEVETDKDKPSDPPAKSSSTNLRARISRFAST

EQLQQRWRKSFKGGGSKVNREAADKIDHADPAPGVAPTTGVLGGLLLGSQLEKTLAKLNEKMHQLKFFQRYSNRNQEPAQ

PKPNTVGHEPVEIDDDEELAATYHHSDSDEDEDGDGDSGEDISAEEAFGKIQEEEDRYQNSQDNSRRGSSSLSGAAPAHA

ARQMAKLAEIQAASKSGAAAWSSESLEEIADEDYPRVLIHQEHSDAFESTLIIAVASKGSVSVSPGVRSSGSQTSGYPVY

PVIKCSSPCPQNASSGGVTHPAFNRSPSGYPEVKRSDAGVKSSPSPEFKTPAAKTPDASAWLPNEQIIAGFKDQSNSSWP

APALYKPKSIDIFEAGSGGSSAFADFDEALRNAPVLRISAGSSCDTSGEEADDSSSRVTRIRVQSPQIGNSRESLMAQEE

EDEEADMEMEMEMEGVLDLDLDLERDSSERPPSASPPPPPLPQRRPPPPRIPATPPIYDAVPPPLPVSKPPIPDKAPTTS

ACVKSPTPPVTRENPPVSKENPPLGRGIPQRSASMSRPAKPLVKTSSLRLTYNEQVRPGDVGKVNKLISRFEGGRPRLCP

RRMHSEEYERGREEDEEEEDAEPMLELKNFQNRAVDSVTPTNRGVIIPQITVNNNNEKANEEGLEEDEIAKSRKKKRESM

ELALDRQNSNCSRSEYGSPLSFPSSRRSSTPTNLSSNPNPHPILNPNANPIQNPVQILQQQRRSRRSMTRDDDNFYSFDS

DEENSYYSISPSGSSRYVVEI

>Dan_XP_001966994

MQQQQQFQPFQHLHHHHHHHHHHQQQQQIQQVQSDSQLEHRNSPDPEAERNPNRDRQQNRSSMDFRNLCQRLDVDSLRAK

LPSLPQIKLPKALPKLRGRKIFRSSKSGSTRANAGSTKDRGYTGPPHQQSYQSQLQVAATSQQFINRTPQRISTISSLMY

EGGGGGGEAGEDIRRSESQPGTYRSAGSLDDDYYAPGSGERASRPISPIKMPSGSEEAAGGSRPSQPPTLTQRIQKGYKS

LSELRLKHIFAKQTTVRRDNIEVDRYVEQYERELRSEKRARERRDREIAENYDIKIKTLAGTRQNTFEEDEVEHEQFERR

DKGHQDTDESGLEATPPLPSRRKPGIAATRFAKVRKPPLEMEAAGQEHDPDQDEEAKASPPKENQPPPRSSSYKQLLSKL

PHLPSLPNLPQLSRTKAEKPPSEEVAPTKSGLRQNIKRIRKSIKRPAKLKPKAPTSQDTDDEEESEEKKEPGKDPASKPP

SSATNLRARLGRFASTEQLQQHWRKSFKRTGKDEAPKEETAAVAGGVLGGFLLGSQLEKTLGKLNEKMHQIKFFQRQGQA

AGAASKQPKPNTVGHEPVELDDDNELEATYHHSDSDDSGEDVSADEAFGQIQPETSPEDPIRRKKPLLAEPREREREKEA

PAPAPPPAKDQAALPAAKAAHASRQMAKLAEIQAASKGGAGTTAWSSESLEEIADEDYPRVLIHQEHSDNFESTLIIAVA

TKSPSPQQLPAPKASPWLPNDQIISGFRQEREREKEQAWPAPALYKPKSIDIFEASGGGGTASAFADFDEALRNAPVLRI

SAGSISSSIEEKDEGHTRVTRIRVQSPQIGNSRESLMAQDEEEEEEEEEEGEPEEEDEDDEEEDLEMELPVEAIDLDDSL

SERPPSASPPPPPLPQRRPPPIPKVIDNTPIYDAVPPPLPITKPPPNEAKAPEAPTTAPTPVPARVSLPQRSASMSRPAK

PLVKTSSLRLTYNEQVRPGDIGKVNKLISRFEGGRPRLCPRRMHSEEYEREEEDELRQEEVLVQKIRVIDSLTPTNRNVV

VIPQITLNNNSDREEKPKGESPLTKDTKDLTLDRQNSNCSRSEYGSPLSFPSNRRSRTPTNLNNNNNPTTSPSNPRQNQN

PSQNPNQNQNPITIQHQRRSRRSMTRDDDNFYSFDSDEENSYYSISPSGSSRYVVEI

>Dbip_XP_017094577

MQQPQQFQHFHHHHHHHHHQQQAIQQVHSDSQLEHRTSPDLERNRDRDRQQNRSSMDFRNLCQRLDVDSLRAKLPSLPQI

KLPKALPKLRGRKIFRSSKSGSTRANAGSTKDRGYSGPPQQQQYQSQLQVAATSQQFINRTPQRISTISSLMYEGGGGEA

SRDDIRRSESQPGTYRSAGSLDDDYYAPGSGERASRPISPIKIPSGGEEAAGSSQPPTLTQRIQRGYKSLSELRLKHIFA

KQTTVRRDNIEVDRYVEQYERELKSEKRARERRDREIAENYDIQIKTLAGTRQNTFEEDEVEHEQFERNKGHHHQDTDES

GLEATPPLPSRRKPGIAATRFAKVRKPPLEMEASGDQEPDPDQDREVKTSPPKENQPPPRSSSYKQLLSKLPHLPSLPNL

PQLSRAKPEKEAKPEATTKSGLRQNIKRIRKSIKRPAKLKPKAPASQDTDDEEESEEKKDANKDSNKAPTSSTNLRARLG

RFASTEQLQQRWRKSFKRTKDETPKEETAAVAGGVLGGFLLGSQLEKTLGKLNEKMHQIKFFQRQGQAAKEPKPNTVGHE

PVELDDDNELEATYHHSDSDDSGEDVSADEAFGEVQPEASPEHLRKKKPLLVEREKDSAAKEQAEDQASLPAAKAAHASR

QMAKLAEIQAASKGGAGTTAWSSESLEEIADEDYPRVLIHQEHSDNFESTLIIAVATKNPTPSPAASPPKASPWLPNDQI

IAGFKQEAAWPAPALYKPKSIDIFEASGGGTAFADFDEALRNAPVLRISAGSIASSIEEKDEGHTRVTRIRVQSPQIGNS

RESLMAQDEEEEEEEEEPEEDEDDEEEDLEMELPVEAIDLDDSLSERPPSASPPPPPLPQRRPPPIPKAIDNTPIYDAVP

PPLPITKPPENVAKTEPIPSSNPQPVAARVSLPQRSASTSRPAKPLVKTSSLRLTYNEQVRPGDIGKVNKLISRFEGGRP

RLCPRRMHSEEYERACDEEEEVLQEVLLQKVRAIDSVTPTNRNVVVIPQITLNNNSNTEREPEKPKEKVTTADPSLDRQN

SNCSRSEYGSPLAFPSNRRSSKPTNNNNNNPTIMPSNPNPNPITIQHQRRSRRSMTRDDDNFYSFDSDEENSYYSISPSG

SSRYVVEI

>Dper_XP_026845977

MSHQQFHHYHHHDQLQQVQSESVLKGNSSSDLGCERNPQNLHNPGHSTTARGIGGGGSMDLRNLCQRLDVDRLRASLPQL

KLPKSLPKLRGRKVFRSSRRNHSSNSNKDGAGGAAVKGVDGGGMCTLPLPLPQQSQQFINRTPQRISTISSLMYEEQQQE

VGTGMGMGMGQGTGHRQPSTYRSAGSLDDDYYAPDSGVGDRASRPISPVKIPSAPVEGGGQPPTLTQRIQRGYKSLSELR

LKHLFTKQTILRRDDIEVDRYVEQYEAELRSERRALARRDREIADNYDIRFKTLARSGQNSPDREAGQAEERTKTQKPRS

VAEQSGDESGMEGTPPLPARSRKPGIAATRFAKVRQPPLQMEEQEVQREREEQPDREASPRRSSYKQLFQKLPSFPNTLH

SLQKGRSKEEKGAGEAASSTPQTPPPKSAIRKNIKRLRKSIKRPAKLKEKQQQQQKAVDDSDEEPQQGRESDRDKEPPAK

SSGNLRARLSRFASTEQLQERWRKSFKTAATKSQESPKGGAGAAAGAGVGAATGMAIGVQLERTLTKLNEKMHQLKFFPR

QGGAASSPSETVGGQSKPNTVGHEPVFIDDDEELAATYHHSDSDEDEEERGSQDSGHDVCAAEAFGQQEDDSEDGDEDEE

EETQMEHPLPGTASAHAARQMATLAELQAEAAKGGCPNGGRSFAAWSSDSLEEIPDEDYPRVLIHQEHTDDSCESTLIIA

VATKTSKASSPALVRSPTPTPTPTPTPTGTTTQWLLNEPTEQQPGGAEEAECWPGPRPLCKPKSIDIFEASGAAFADFDE

ALRNAPILRISAGSSCDTSGEEADDSSSRVTRIRIQTSPKIGNSRESLMAQEREDEQEQEQEEAKDAVEEEEMERRALER

PPSASPPPPPLPKRRPPQRRIFTPPPAPIYDAVPPPLPTSKPPPLSMTIPPNRAASPAVPASLPAVSSLPQRSRASMTRP

AKPLVKTSSLRLTYNEQVLPGDIGKVNKLISRFEGGRRPRLCPRRLHSEEYELTATSASFNWDEDQDDAAEEEDNGQKEE

AASPITPTNRPVVIPEIVTTQPSNNNNNNNNSHRRREKEEEELLTMELSLDRQNSNCSRSEYGSPLAYPYPSRRRSSAAT

GAPPFTLPAPQQQQQQQQQQQQQRQQARRSRRSMTRDDENFYSFDSDEENSYYSISPSGSSRYVVEI

>Dpse_XP_002134634

MSHQQFHHYHHHDQLQQVQSESVLKGNSSSDLGCERNPQNLHNPGHSTTARGIGGGGSMDLRNLCQRLDVDRLRAKLPSL

PSLPSLPQLKLPKSLPKLRGRKVFRSSRRSHSSNSNKDGAGGAAVKGVDGGGMCTLPLPLPQQSQQFINRTPQRISTISS

LMYEEQQQEVGTGMGMGMGMGMGQGTGHRQPSTYRSAGSLDDDYYAPDSGVGDRASRPISPVKIPSAPVEGGGQPPTLTQ

RIQRGYKSLSELRLKHLFTKQTILRRDDIEVDRYVEQYEAELRSERRALARRDREIADNYDIRFKTLARSGQNSPDREAG

QAEERTKPQKPRSVAEQSGDESGMEGTPPLPARSRKPGIAATRFAKVRQPPLQMEEQEVQREREEQPDREASPRRSSYKQ

LFQKLPSFPNTLHSLQKGRSKEEKGAGEAASSTPQTPPPKSAIRKNIKRLRKSIKRPAKLKEKQQQQQQQQQKAVDDSDE

EPQQGRESDRDKEPPAKSSGNLRARLSRFASTEQLQERWRKSFKTAATKSQESPKGGAGAAAGAGVGAATGMAIGVQLER

TLTKLNEKMHQLKFFPRQGGAASSPSETVGGQSKPNTVGHEPVFIDDDEELAATYHHSDSDEDEEERGSQDSGHDVCAAE

AFGQQEDDSEDGDEDEEEKEETQMEHPLPGTASAHAARQMATLAELQAEAAKGGCPNGGRSFAAWSSDSLEEIPDEDYPR

VLIHQEHTDDSCESTLIIAVATKTSKASSPALVRSPTPTPTPTPTPTPTPTGTTTQWLLNEPTEQQPGGAEEAECWPGPR

PLCKPKSIDIFEASGAAFADFDEALRNAPILRISAGSSCDTSGEEADDSSSRVTRIRMQTSPKIGNSRESLMAQEREEEQ

EQEQEEAKDAEEEEERERRALERPPSASPPPPPLPKRRPPQRRIFTPPPAPIYDAVPPPLPTSKPPPLSMTTPPNRAASP

AVPASLPAVSSLPQRSRASMTRPAKPLVKTSSLRLTYNEQVLPGDIGKVNKLISRFEGGRRPRLCPRRLHSEEYELTATS

ASFNWDEDQDDAAEEEDNGQKEEAASPITPTNRPVVIPEIVTTQPSNSNNNNNNNNSHKRREKEEEELLTMELSLDRQNS

NCSRSEYGSPLAYPYPSRRRSSAATGAPPFTLPAPQQQQQQQQQQQQQQQQRQQARRSRRSMTRDDENFYSFDSDEENSY

YSISPSGSSRYVVEI

>Dhyd_XP_023176687

MSHRQYQQEPHQQTPERNGHSHQGIMDLRNLCQRLDVDHLKAKLTALPQLKLPKSLPKLRAARRIFRSSRGNGSGKEAPA

AGAAAELQSEASGLAQAQFINRTPQRISTISSLMHEQAAPQHLQPNAGSTYRSAGSLDDDYYAPYGTAARASRPISPVKM

PPTLALGDAAMAMAMPTPTPAQTQTTTLRNGSSLTQRLQRGYKSLSELRLKHLFAKQTVIRRDGIEVDRYVEQYEAERRV

EQQAQARRDRQIADNYDIHISTLPITRENTLRAQQQLEEQALPQHRRSVAEHSGDESGMEEAMPQQQLPPVPRKKPGIAA

TRFSRVRQPPLPMPVEAHAEEQQQRPKEPQPVRQAVRQNLKRLRKSIKRVHQPAARPGQADDTDDDEEPQQQQQQQRQPQ

PKTRASTGGTLRARLRRFASSEQLQQRWRRSFKSNSESQDQSAQSSGGAAASGAAFGFGMGAHLERTMTKLNEKMQQLKF

FQRSGADKAATAEAGNKPNTVGREPVEVDDEELEATYHHSDSSDSEDSGQDVCADEAYGQMELEPQRALSPALVKRQTLA

SLAQLHANASTAWSSESLEEDATVEQQMAAAASDYPRVLIHQQHTDAYESTLILAVAATPPPKPSPVVEKHQWVSNQQII

ADFKASVWPAPALYKPKSIDIFEGDGGAAAAFADFDEALRNAPVLRISAGSSCDSGEDADDSSSRVTKIRLQTPQIGNSC

ESLMEQQPLEDEEEVEDKNQTADAEADVDADVDADVDADMDMDMDVDLDDQEARMAERPPSESPPPPPLPQRRPPPPSIV

APIYDAVPPPLPISKPPPIQAASATPAPVAAAPLVVAAAASSVASLPQRSTSMTRPSKPLVKTSSLRLTYNEQMHPSDFG

KVNKLISRFEGRPRLCARRLHSEELTTCSDADDEPEPEPAKQEQQQTPTNAKQSAIPNIVTTQPNHSNNNNNNNVSSSAQ

DQAELQLSLDRQNSNCSRSEYGSPLAYPLSNSRRRSSTPTIASTLNAPALAPQSQLQSQRAQARRSRRSMTRDDDNFYSF

DSDEENSYYSISPSSSSRYVVEI

>Dobs_XP_022208891

MSHQQFHHYHQHHHLHQVQSDSQLERTLGTERNQHQQHPNPNRNSNTERVSRIGSMDFRNLCQRLDVDGLRAKLPSFPSL

PPLKLPKSLPKLRGRKIFRSSRRNNSSSNTHKDVAGAGGGGATAKGGDAGGMCTLPLPLPPQSQQFINRTPQRISTISSL

MYEEQQEGRGRGQGQGQPGTYRSAGSLDDDYYAPESGGGVGGGVGERASRPISPVKMPSPPAGSQREGQSQPTLTQRLQR

GYKSLSELRLKHLFAKQTVVRRDNIEVDRYVEQYEAELRSEQRAQARRDRQIADNYDIRFKTLADGEEEGHAGHARHARQ

EERSKKPQKPRSVAEQSGDESGMEGTPPLPTRTRKPGIAATRFAKVRQPPLQMEEEDKHEMEMELVVEGEGEVAKEQREA

APRRSSCKQLFQKLPSFPNSLASLQKRSKVSEEKGEKAEKAEKGGQGEQTSPPAKSAIRQNIKRLRKSIKRPARRKQPQK

QPQKSVDESDEEEQQQQEQGQGQGQEEGAPPVAKSSGNLRARLIRFASTEQLQERWRKSFKTAGQDAQRSPKAKADDGAG

AGAGAATGMAIGVHLERTLTKLNEKMHQLKFFQRQGAAAASTEEVGGVQPQLQPKPKPNTVGHEPVYIDDDEELAATYHR

SDSDEDDDEDEAERGSQDSGQDVCAEEAFGQQDEDEDEDEDGESGEETQMEHPLSGASSAHAARQMATLAELQAAAAAAA

EGRRSFTAWSSESLEEIPDEDYPRVLIHQEHTDDSCESTLIIAVSTKPSPSPLPSSPAPPTPSGSGAWLPNAEIISSFKE

QQLGEAESWPGARSLYKPKSIDIFEASGPAFADFDEALRNAPILRISAGSSCDTSGDEEADDSSSRVTRIRMQPDAPPRI

GNSRESLMAQEHEEEEEEEAEEESTKEDERIWERPPSASPPPPPLPQRRPPQRKATPPSPAAPIYDAVPPPLPTSKPPPL

SMTPPAAAAAPAAVLPQRSSASMSRPAKPLVKTSSLRLTYNEQVHPGDIGKVNKLISRFEGGRRPRLCPRRLHSEEYELH

ATSSSFSGGEEEEEKEDDAEEQQEQEEEAASPITPTNRPVVVIPEIVTTQPSISNNNNNNNSGSGSSRRRKEESLSLSLD

QCLDRQNSNCSRSEYGSPLAYPYPSKRRSSTGAAPPSQLTPPQQQMLQQQQQQQQLQLQQAARQQARRSRRSMTRDDENF

YSFDSDEENSYYSISPSGSSRYVVEI

>Dwil_XP_023035026

MDFHNLCQRLDVDNLRAKLPALPQIKLPKSLPKLRGRKIFRSSRDNVNNNKAGTAMAGGGGGGGVGDTSHHHTSLSSSSL

QLPQQQQQSQQFINRTPQRISTISSLMHEQQERMGPTSTYCSAGSLDDDYYAPDQHHNNRDVHRPISPIKMPSNQTESGT

VERSSLTQRLQRGYKSLSELRLKHLFAKQTTVRRDQIEVDRYVQQYEAELRSEAKAQARRDREIADNYDIRIKTLATPQT

SRQNTLDEGNEADVATPPPTIKQRRSVAEHSGDESGMEEEATPPAARRKPIGSGIAATRFAKVRQPPLQMPVNEEATATT

PPEPPRPPSGGAYKQLLQRLPASLTSLPTLSRGKEKRESTPENAAEPSKLSTLRFPMKSKTSVEDSPPQPPADGASSKGL

GLRHNIKRLRKSIKRPLKATARTTTATTSQESGDEDQTNARPNSSVTLRTRLSRSTDQLQQRWRKSFKFSTESDPSEANA

DGAGGAAAGAAYLERTFNKLNEKMHQLKFFQRQSSNVGVASPPSRKGPKPNTVGHEPIEIDDEELAATYHHSDSDDDDSG

QDVCAEEAFGQVDEDAAAGGNKPIPANTSTNPRREMAKLAELQAKRSSTAWSSESLEEIPDEDYPRVLIHQEHTDAFEST

LIIAVATAPPPIPPPPVATNWLPNEKIIASFQEQVWPAPALYKPKSIDIFEASSVGGAAGAFADFDEVLRNAPVLRISAD

VEEEDEEEEEEEDEEEDDSSSRVTRIRVRSSSPQIGNSRESLMEAQQEEDEEEEDKEEMAARDKSSPETERPPSTSPPPP

PLPQRRPNQVPVPVPVPPVPIYDAVPPPLPTSRPPLPLPLPIVSQAIVPPQRSASMSRPSKPLVKTSSLRLTYNEQVRPG

DVAAEEETRVEIEAMAKTIETDVTRTPTNRTPIPEIVTSQPVNENLPEAKGKVTKTTTSTEIQLNLDRQNSNCSRSEYGS

PLAYPSSRRCSTPTVSKLPPPPLTSSSSLTSGAGGLVAAPAGHSQAHLRQINRRSRRSMTRDDDNFYSFDSDEENSYYSI

SPSGSSRYVVEI

>Dari_XP_017870878

MSHLQYQQEPQQQPPERSGHSHQGIMDLRNLCQRLDVENLKAKLTSLPQLKLPKSLPKLRAARRIFRGSRGNGSGKEAPA

ATAAELQSEVSGMGMAQAQFINRTPQRISTISSLMHEHEHDQVAPQNLQPSAGGTYRSAGSLDDDYYAPYGAAARVSRPI

SPVKVPPTMAPGDASMPTPTPTLRNSGSSSLTQRLQRGYKSLSELRLKHLFAKQTVVRRDGIEVDRYVEQYEAERRVEQM

AQARRDRQIADNYDIHISTLPITRENTLKAQQQQQQQHRRSVAEHSGDESGMEEAIPQQQQVPPVPRKKTGIAATRFARV

RQPPLPMPLEDQSEEQQPQKPREPQAVRQAVRQNLKRLRKSIKRVQQPAARPAADDSDSDEEQQQQQQRKSKTRSSTGDT

LRARLRRFASSEQLQQRWRRSFKSNSDSQEQAAQQPSGAASGAAFGFVGMGAHLERTMTKLNEKMQQLKFFQRSGADRAE

AGNKPNTVGREPVEIDDEELEATYHHSDSSDSEDSGQNVCADEAYGQMEVEPQPDASPTVLAKRQTLTSLQASASTAWSS

ESLEDDATVEQQLAAAASDYPRVLIHQQHTDAYESTLILAVAATPPPVPSPVALEKHQWVSNQQIIADFKAQQMSSPSAW

PAPALYKPKSIDIFEGDGGAAAAFADFDEALRNAPMLRISAGSSCDSGEDADDSSSRVTRIRLQTPQIGNSCESLMEQQP

LEDDDEVPNKIEAMDLDADADANVNVDANANANVDQDVDLARLAERPPSESPPPPPLPQRRPPPPSTVAPIYDAVPPPLP

ISKPPPPPMQAAPLAPAPLPVAAPLTLGTLPQRSASTTRPSKPLVKTSSLRLTYNEQMHPADVGKVNKLISRFGGRPRLC

ARRMHSEELTTCSDADDEPQPEAEPEPQLKTQLQQPKADKQQQQQQQTPTNAKPSAIPNIVTTQPNNNNNNSNSAKTETE

LQLSLDRQNSNCSRSEYGSPLAYPLSNSRRRSSTPTVAHTLNANSSALALAPQSQRAQAQAQARRSRRSMTRDDDNFYSF

DSDEENSYYSISPSSSSRYVVEI

>Dvir_XP_002055383

MQYHQYQREPQQQPSERNGHSHHSVMDIRNLCQRLDVDSLKAKLSTLPQLKLPKSLPKLRAARRFFHSSHENGSGKAAPG

TGTGIGTSTGTGTGTGTDSLAPAMPQAQFINRTPQRISTISSLMHEQAAVDRQVTAATTQQLQSNASTYRSAGSLDDEYY

APYGTGGRGSRPISPIRMPPDTTLTTPTTTTTTTTSSLTQRLQRGYKSLSELRLKHLFAKQTTIRRDGIEVDRYVEQYEA

ERRLEQLEQARRDREIADNYDIHISTLPMSRQNTMKEEEDQQQQQQQQQQDQHGRSVPEHSGDESGMEEAMPQQQAPPRK

KTGIAASRFAKVRQPPLPMPVQEQQEQAEQQRQKEQQPMRQAVRQNLKRLRKSIKRVHHQPARPAAEDTDEDEGQTKTRT

STGGTLRARLRRFASSEQLPQRWRKSFKSNSETQEQSAQQLPGGAAAGTAGASAGATLGLAVGAHLERTMTKLNEKMQQL

KFFQRAGDKKSAEQMGNKPNTIGREPVDIDDDELEATYHHSDSASDDSDQEMVTAEVEQERAQQQKERVAASPGLVKRQT

LTTLAQLQAHSTTAWSSESLEEDASAEQQLATAASDYPRVLIHQQQTDAYESTLIIAVTATPPSSSPPAPIAAGKQRSTA

HQWVSNEQIIADFKAQQATSSVWPAPALYKPKSIDIFEGDVGAAAAFADFDEALRNAPVLRISTGSSCDSGEDADDSSSR

VTKIRLQTPQIGNSCESLMEQQPLEDEEEDDDAAEEDDEDDDDDDDDEQALAERPPSVSPPPPPLPQRRPPPSTVAPIYD

AVPPPLPISKPPIATAIQTTAAPTATAPLATAALAAAAMPLGLAKLPQRSVSMTRPSKPLVKTSSLRLTYREQMHPGDVG

KVNKLISRFEGRPRLCARRLHSEELTMCSDADDEPDQEPDKNIKKEKEMAEQQLPPTPTPANVKRNVIPDIVTTQAISNN

NNNNNQNHNNNNNNNNISGHTSSRDEAQLQLSLDRQNSNCSRSEYGSPLAYPLSNSRRRSSTPTMATALNTSSSLHAFTP

QSQSQSQLLQQQQQQQRAQARRSRRSLTRDDDNFYSFDSDEENSYYSISPSSSSRYVVEI

>Dmoj_XP_002010583

MSHLQYQQEPQHQPPERSGHSHRGIMDLRNLCQRLDVENLKAKLTSLPQLKLPKSLPKLRAARRIFRGSRASGSGKKAPA

ATAAELQSEASGMDMGMGMAQAQFIDRTPQRISTISSLMHEHEHDQVAPQHLQPSAGGTYRSAGSLDDDYYAPYGAAARV

SRPISPVKVPPTMAPGDAAMPTPTPRNSGSSSLTQRLQRGYKSLSELRLKHLFAKQIVVRRDGIEVDRYVEQYEAERRVE

QMAQARRDRQIADNYDIHISTLPFTRENTLKAQQQQHRRSVAEHSGDESGMEEAIPQQQQVPAVPRKKTGIAATRFARVR

QPPLPMPLEDQSEEQQPQKPKEPQAVRQAVRQNLKRLRKSIKRVQQPAARPAADDSDSDEEQQQQQQRKSKTRSSTGDTL

RARLRRFASSEQLQQRWRRSFKSNSDSQEQAAQQPSGAASGAAFGFVGMGAHLERTMTKLNEKMQQLKFFQRSGADRAEA

GNKPNTVGREPVEIDDEELEATYHHSDSSDSEDSGQNVCADEAYGQMEVEPQPDASPAALAKRQTLTSLQAGASTAWSSE

SLEDDATVEQQLAAAASDYPRVLIHQQHTDAYESTLILAVAATPPPVPSPVALEKHQWVSNQQIIADFKAQQMSSPSAWP

APALYKPKSIDIFEGDGGAAAAFADFDEALRNAPMLRISAGSSCDSGEDADDSSSRVTRIRLQTPQIGNSCESLMEQQPL

EDDDEVTNKIEAMDVDVDADVNVDANANVDQDVDLARLVERPPSESPPPPPLPQRRPPPPSTVAPIYDAVPPPLPISKPP

PPPMQVAPLAAAPLAAAPLAAAPLAAAPLPVAAPLTLGTLPQRSASTTRPSKPLVKTSSLRLTYNEQMHPADVGKVNKLI

SRFGGRPRLCARRMHSEELTTCSDADDEPEPEAEPEPQLKTQLQQPKADKQQQQQQQQQTPTNARPSAIPNIVTTQPNNN

NNCNNNSAKTETELQLSLDRQNSNCSRSEYGSPLAFPLSNSRRRSSTPTVAHTLNANGSALALAPQSQRAQAQAQARRSR

RSMTRDDDNFYSFDSDEENSYYSISPSSSSRYVVEI

>Dnav_XP_017964184

MSHLQHQQEPQQQPPERSGHSHQGIMDLRNLCQRLDVENLKAKLTSLPQLKLPKSLPKLRAARHLFRGSRANSSGKEAPA

ATAAELQSEASGMGMGMGMAQAQFINRTPQRISTISSLMHEHEHEHDQVAPQHLQPSAGGTYRSAGSLDDDYYAPYGAAA

RVSRPISPVKVPAPGDAAMPTPTLRNSGSNTLTQRLQRGYKSLSELRLKHLFAKQTVVRRDGIEVDRYVEQYEAERRVEQ

MAQARRDRQIADNYDIHISTLPITRENTLKAQQQQQQQALPQHRRSVAEHSGDESGMEEAIPQQQQVPPVPRKKTGIAAT

RFARVRQPPLPMPLEDQAEEQQPQKPKQPQAVRQAVRQNLKRLRKSIKRVQRPAARPTADDSDSDEEQQQQQQQQQQQRK

SKTRSSTGDTLRARLRRFASSEQLQQRWRRSFKSNSDSQEPAAQQPSGAAFGFVGMGAHLERTMTKLNEKMQQLKFFQRS

GADRAEAGNKPNTVGREPVEIDDEELEATYHHTDSSDSEDSGQDVCADDAYGQMEVEPQPDASPTALVKRQTLTSLQASA

STAWSSESLEEDATVEQQLAAAASDYPRVLIHQQHTDAYESTLILAVAATPPPVPSPAALEKHQWVSNQQIIADFRAQQM

SSPSAWPAPALYKPKSIDIFEGDGGAAAAFADFDEALRNAPMLRISAGSSCDSGEDADDSSSRVTRIRLQTPQIGNSCES

LMEQQPLEDDDEVPNKTEAMDVDADADADTNVDANANVNVNANAHADQDEDLARLVERPPSESPPPPPLPQRRPPPPSTV

APIYDAVPPPLPISKPPPPPMQAAPLAPAPPVVAPSTIGNLPQRSASTTRPSKPLVKTSSLRLTYNEQMHPADVGKVNKL

ISRFGGRPRLCARRMHSEELTTCSDADDEPEPDPEPEPEPQLKSPLQPKADKQQQLQQQQTPTNAKPSAIPNIVTTQPSN

NNNNNNNDSAKTKTEQQLSLDRQNSNCSRSEYGSPLAYPLSNSRRRSSTPTVASTLNANGSALALAPQSQSQRAQAQARR

SRRSMTRDDENFYSFDSDEENSYYSISPSSSSRYVVEI

>Dgri_XP_001992749

MTHLQQQQQQDLQREPQQRNNHSQHGIMDLRNLCQRLDVESLKGKLASLPQLRLPKSLPKMRAARRIFCSSRGNNASGSN

KAATTAGETAVETEQVPAALFINRTPQRISTISSLMHEQATLGANRGQMAPSSSNHLAAGTYCSAGSLDDDYYAPYGNTR

VSRPISPIKIPPPPTAEAAATRNGGNSLTQRLQRGYKNLSELQLKHLFAKETIVRRDGIEVDRYVEQYEAERRVEKLAQE

RRDREIADNYDIHIRTLPLTRQNTLKDLQQEQLAETARHESDDESGMEEAMSAPVPPTQRKKSGIAATRFAKVRQPPLEM

PVQEQQQHQQEQQQEQGQPPIRRAMRQNLKRLRKSIKRVHQHQPKQAVDKQASKDRDDVNDDEEEDGDLPKPSQSGVAGG

TMRARLRRFTSSEQLQQRWRKSFKSSSETQVAQGAQGAAVGAAAGATFGMGMMHKLNEKMQQLKFFRQRGSGDSSKDKPP

AATQLGGNKLNTVGQEPADIDDDEELAATYHHSDDSDDSNGSDDSGQDVCADEAFGQLDAGEQVKRERERDRPTIGMLAQ

LQAHSTTAWSSESLEEEDAALGGQQQLAAAGDYPRVLIHQQHTDAYESTLIIAVATTLTPPPTRDNRHQWVSNEQIIAGF

KEQQQLTAQACNSSRVWPGPALYKPKSIDIFESEGGPAGAFADFDEALRNAPVLRISAGSSEEDADDSSSRVTRIRRQTP

QIGNSCESLMEQQPLEEDDDNDNNGNNEDMDMDMEMDVDVTVDDEQLELAERPPSVSPPPPPLPQRRPPPSMMAPIYDAV

PPPLPISKPPLTIVAAATAATAAPAPAPVPVASPAAAKLPQRSVSMTHPSKPLVKTSSLRLTYREQMHPGDVGKVNKLIS

RFEGRPRLCGRRLHSEELTICSDEDEPADDLFEQMQVESVSQLTPTNVKCKPIPDIVTTPTPTPTTSNNNSCHNLSRDLS

REQLELSLDRQNSNCSRSQYGSPLPFPNSQSQRRSSTPTMPTTSISSRSARSLAPQTHSHSQSQLARSNRRSMTRDDDNF

YSFDSDEENSYYSISPSSSSRYVVEI

**>Rzep_XP_017474957**

MDFKNLCQNIDVSSLRARIPSLPAMPAMPQIKLPKSLPKLRPRRIFSRSREDLSRDSKGSKKEQQLQQQQQHSPFQQLSV

RPPIPPADFVNHSPQRISTISSLMHQQKRGDSQRGTYRSACSVDDDYPRPREFEVPERLSRPISPIHTSISGVTNERDAE

GGAVAKMSLSEKLQKGYKDITEFRLSHIFAKKTVVRRDVIQVDHYVERFNEDRERERAEQEQRDRKIADNYRFNFKVSRQ

DTDNSKRSMSSDDSPKQEPQKGGKTVHVQSEDESGMEATPPPRKPGIASTRFARVRNPPLEQYISDESNNELDEDEELPV

ARGGHQKQRIVQSSPESGPKAPAESRGAKALYKLRSLGKRSEETPEKLGEKKRRAPLTPQQSEEKPELPIAAENDNPLSV

LKQNIKRFSKSIRRTQEADSSGAANAAEDTEGEGTPKKETKQISRTEKLAARLRKFASHEASTENLDETASPKQSEEQRS

PIRAAISNKLQTWKKSFKRKPNEIDGAQTGEETSPEREGDKEKRTDKLMKKLRNMRQRKRASSTDDLDGTEDEANQTPTG

KSKDDNCRRAVNFEERFEQARKRTLKKVNEKMQQIKFFHKSQENIEKESGGGSKKDAGKPAKQEYEDQSDDERTIYVRPH

TAARRAAARDTPSDSEEESQPEREEHVEREFDGTHIQHTRAIWTTKNLLTDSLDSETDTELPRVLIHQDNSDQFESTLII

AVTRPAPPVTPTTPIIEEIHDEPPMKPLRASRTPSPGSSRAEWIPNNEIISSFLEITPPSRRRLSSEAILPAPRGHLRKM

SLDSNSDSDSWIPEAARAKKLDQQSREGSEEGASAATLNSIDEKEEPWKVHHTHCDDKLYKTRSIDIFEASVRKGGAIAA

FDDFEEELRDEPILKVPQRHIDSSFEKDSSEEPFAADDDNSSRVTKILRSVIREECEEVQENVDEKLEMKLPQVPQRKFS

NSSESLIANIDENESERIKLREKDNDENKDYDDDEDDDDRPPSAAPSPPPSMSPPPPPLPLRKSVLITSKTTEEGSPPLP

TTKPPVPPIPPAKPSQPPRIPDRTPSMTRIAKPLVKTSSLRLAYNEQVNPSDVGKVNKLISRFETPQSKLTQRPRIIRRG

LTRDESEEYSDDDDDLEEDEDEDGEQDTDEANANAERDVTPTPTNTPRTYSLESETESLHVISKHATTTKPRTLSLEETT

TTTNRSIVGNSNQTKVYSEVPRITLNYDNNSNLSLSRASSEYGSPLEYPSSLIGSTETTPIPERRPPDLSTLRNAERNRR

SMTRDDDKFLSFDSDDENSYYSISSTGSSRYVVEI

**>Blat_XP_018782934**

MDFKNLCQNIDVSSLRARIPSLPAMPAMPQIKLPKSLPKLRSRRIFGRSREDLSRNIGSTKEQQRQQSIRQHPTHPPIPP

ADFINHSPRHISTVSSLMHQQQQEQRGDSQRGTYRSACSVDDDYPSPRQFQTAGHISRPISPIHTSGNDGKSEHVEPTVA

KLSLAEKLQKGYKDISEFRLSHIFAKKTVVRKDVILVDQYVERYNEEREHEQAEQERRDRKIADNYRFNFKLSRQDTDNS

KHSVSSDESHKQVGNRRAKNIHDQSEDESGMEATPPPRKPGIASTRFARVRNPPLEQYITDESNNELEEDDEIPAPIAAK

QKSKIVQSSPESMSKGVVNRNALSKLRNMSQSSEETPEKLGERKRRRAPLSPQQSEEKQEVPAATVNENNPLSVFKQNIK

RFSKSIRRAQEPETSATNANTAEDTDGDGTPKKETKQISRTGKLAARLRKFASHEASTENLDEATPKKTEEQRSPIRIVI

TNKLQNWKKSFKRRPTESEAQTGDETSPERNAEKGEKRTDNLMKKLRNIRQQKRAHSTDDLDGTEEDEAHNTPTGTTKDA

NARKTVNFEKRFEQARQRTLKKMNEKMQQIKFFHKSQENLDKECSATTKEASKHDEVDAEEESEDERTVYVRPHTAARRA

AARDTPSDSEDELQQTAGREHLKTDKLIQNENEYDDEDMQHMRTLWTTKNLLNDSLDSETDVAFPRVLIHQDNSDQFEST

LIIAVTRPAPSKPSTPIIEEIRDDAPKFKPPRRSRSPSPGSGRAEWIPNNEIISNFLEITPPSQRRLSAEAILPASRGHT

RKISMDSNSDSDSWIPENARHKKISLPSKEDSMETAIGITLNSIDENEEPWKVYQHAYCDEKLYKAKSIDIFEASVRKGG

AKAAFDDFEEELRDEPILRVPQKHIDSSFEKDSSEEPFAADDDNSSRVTKISVSVIREECEKECENNKKNIKLPQGSPKS

DGFKAHLDEHERKIVHKMDENSDKDEDDDDDRPPSIAPSPPPSVSPPPPPLPLRKPILLPSKSMEESPPIPTMKPPMPPT

KPPPRIPDRTPSMTRIAKPLVKTSSLRLAYNEQVKPSDVGKVNKLISRFETPQSNVSQRPRIIPKRLTRDESEEYTDDED

DLEEDEDGEQETDAINANSERDVTPTPTNTPRMYSLESDTESLSTVATRVNTTKRTTVKIDENVTITQRGNVSNGNTSKV

YSDLPRITLNFDNNSNLNLSCASSEYGSPLEYPSSLIGSTETTPIPERRPQEVQNLRSAERNRRSMTRDDDKFLSFDSDD

ENSYYSISSTGSSRYVVEI

**>Bole_XP_014088383**

MDFKNLCQNIDVSSLRARIPSLPAMPAMPQIKLPKSLPKLRSRRIFGRSREDLSRNKGSTKEQERQQSIRQLSTQPPIPP

ADFINHSPCHISTVSSLMHQQQQQQQRGDSQRGTYRSACSVDDDYPSPRQFQAPDRLSRPISPIDGESERVEPTVAKLSL

TEKLQKGYKDISEFRLSHIFAKKTVVRKDIILVDQYVERYNEEREREHAEQERHDRKIADNYRFNFKLSRQDTDNSKRSI

SSEESHTQVNNSRAKNMHEQSEDESGMETTPPPRKPGIASTRFARVRNPPLEQYITDESNNELDEDEEVPAPRAAQQKSK

IIQSSPESMSNQAMNRKTLSKLRNMRQSSEETPEKLGERKRRRAPLSPQQSEEKQEAPAATVNENNPLSVLKQNIKRFSK

SIRRAQESEPKSEPNANTAEDTDGDGTPKKEAKRISRTEKLAARLRRFASHEASTENLDEATPQKSEEQRSPIRTVITNK

LQDWKKSFKRRPTESEAQTGDETSPERNAEKGEKRTDTLMKKLRNIRQQKRANSTDDLDGTEEDEAHNTPTGTTKDANAR

KTVNFEKRFEQARQRTLKKMNEKMQQIKFFNKSQENLEKECSATKKEASKHDEVEAEEESDDERTVYVRPHTSARRAAAR

DTPSDSEDEQQQTAGEERVKNDKLTQSDYEYDNEDMQHTRTLWTTKNLLNDSLDSETDVAFPRVLIHQDNSDQFESTLII

AVTRPAPSKPSTPVIEEIHDDAPKVKPPRRSRSPSPGSGRAEWIPNNEIISNFLEITPPSQRRLSAEAILPAPRGHTRKM

SMDSNSDSDSWIPDNPRNKKISQPSKEDSMEAASGVTLNSIDENEEPWKVHQHAYCDEKLYKAKSIDIFEASVRTGGAKA

AFDDFEDELRDEPILRVPQKHIDSSFEKDSSEEPFAADDDNSSRVTKISVSVMGEECKEIKECDNNKANIKLSQGSAKTD

GLNTHLDEQKRKIVHNRDENVDTDEDDDYDRPPSAAPSPPPSISPPPPPLPLRKPILLPSKSMEESTPVPTMKPPMPPTK

PPPRIPDRTPSMTRIAKPLVKTSSLRLAYNEQVKPSDVGKVNKLISRFETPQSNTTQRPRVIRRRLTRDESEEYTDDEDD

LEEDEDGEQETDAMNANSERDVTPTPTNTPRMYSLESDTESLSAVSTNVNKTKRTTVKMEENITITQRGNVVNGNTSKVY

SDLPRITLNFDNNSNLNLSCTSSEYGSPLEYPSSLIGSTETTPIPERRPQEVENLRSAERNRRSMTRDDDKFLSFDSDDE

NSYYSISSTGSSRYVVEI

**>Ccap_XP_004520033**

MDLKNLCQNIDISSLRARIPSLPAMPQIKLPKSLPKLRSRRIFSRSREDLSRSGSSKGATAKDQQLQQPGVHLTLSRQPP

LPPPIPPADFINHSPCHISTVSSLMQQHQQRQQRGDSQRGTYRSACSVDDDYPSPRQFQAPGRLSRPISPIHTPMNDGGG

EEAVVKMSLTEKLQKGYKDISEFRLSHIFAKKTVVRKDVIQVDHYVERYNEERAYEQAEQERRDRKIADNYRFNFKVSRQ

DTDNSKRSMSSEEAPEDAVDTHARKVREQSEDESGMEVTPPPRKPGIASTRFARVRNPPLEQHLDYESNNELDEDEEVPA

PRGVSQQQRKSKMEQSSPESMTQEPVKRGALNKLRRMSKSSEGTPEQTAERKRRRAPLSPQQSEEKLEAPVTTENEHNPL

SALKQNIKRFSKSIRRAHEADGSGIKADETDGDDTPNKQETKHISRTEKLAARLRRFASHDASTENLDETTPKNTEEPRS

PIRAVITNKLQTWKKSFKRRPNETETQTGDDTTPEHGVAEEGKRSDTLMKKLRNMRQRKRASSTDDLDGTEDEEGRNTPA

ATTTSKSKDASSRKTVNFEARFEQARKQTFKKMNEKMQQIKFFHKSQENLEKDEGATTQNAVGRRADMEAARADEESEDE

RTVYVRPRTAARRAAARDTPSDSEEEHQGLQPTDEPRAESFETTRHEYDYNGEHIQHTRALWTTKNLLTDSLDSEMDTEF

PRVLIHQDNSDQFESTLIIAVTRPAPSTPNTPIIEEIHDDLQNVKPPRRSRSPSPGSSRAEWIPNNEIISTFLEITPPSQ

RRLSAEAILPAPRGHTRKMSMDSNSNSDSDSWIPENTRNKKLGQQNKEGSVEANSGVALNSIDEHEEPWKVHQHAYCEEK

LYKTKSIDIFEASVRKGGARAAFDDFEEELRDEPILRVPQKNIDSSFEKDSSEEPFAADDDNSSRVTRISVSVIREEYEE

MQENVVSKEEIKLTEDTVRPVCESENESLIANVDEEERKQLNEEKYFEVRAGDNDDGDDGDDDDVDDDEDEDDRPPSATP

SPPPSMSPPPPPLPLRKPMLLAAKSIEESPPVPNTKPPVPPAKPPPRIPDRTPSMTRIAKPLVKTSSLRLAYNEQVKPSD

VGKVNKLISRFETPQSKVTQRPRVIRRRLTRDESEEYTDDEDDLEEDEESEQEKAANSERERDITPTPTNTPRTLSLESD

VENLLTRANPVTKHQKASAKLEEKNTTPTNRKNSGNGNQPKVYSDLPRITLNFDNNSNLDLSCASSEYGSPLEYPSSLIG

STETTPIPERRPQELQNLRSAERSRRSMTREDEKFLSFDSDDENSYYSISSSGSSRYVVEI

**>Zcuc_XP_011185090**

MDFKNLCQNIDVSSLRARIPSLPAMPAMPQIKLPKSLPKLRSRRIFGRSREDLSRNQAGSAKEQQRQQSIRQPPIPPADF

INHSPRHISTVSSLMHQQQQQQQQPQRGDSQRGTYRSACSVDDDYPSPRQFQAPGRLSRPISPIHTPVNDGGDERVEPTV

AKLSLVEKLQKGYKDISEFRLSHIFAKKTVVRKDIILVDQYVERYNEEREREQAEQERRDRKIADNYQFNFKLSRQDTDN

SKRSVSSDESQKQVDKGRAKNMHEQSEDESGMEATPPPRKPGIASTRFARVRNPPLEQYITDESNNELDEDEEVPVPRAT

QQKPKIVQSSPENMSKGAVNRKALSKLRNTSQSSEETPEKLGERKRRRAPLSPQQSEEKQETPATTVNENNPLSVLKQNI

KRFSKSIRRTQDTEPSGTPANTAEDTDGDGTPKKEEKQISRTEKLTARLRRFASHDASTENLDEATTKKTEEQRSPIRTV

ITNKLQNWKKSFKRRPTESEAQTGDETSPERNTEKSEKRTDNLMKKLRNIRQQKRANSADDLDGTEDDEAHNTPTGTPKD

TGARKTVNFEKRFEQARQRTLKKMNEKMQQIKFFHKSQENLEKETGANKKEANKHEEVEVEEESDDERTVYVRPHISARH

AAKRDTPSDSEEEQQQAAGRERARNDKPTQHEYEYDDDDMQHTHTLWTTKNLLNESLDSEIDVAFPRVLIHQDNSDQFES

TLIIAVTRPAPSIPTTPIIEEIHDDVPKYKPPRASRSPSPGSARAEWIPNNEIISNFLEITPPTQRRLSAEAIPPAPRGH

TRKMSMDSNSDSDSWIPDSARNKKMSLPNREDSVEAASGVTLNSIDENEEPWKVHQHAYCDEKLYKAKSIDIFEASFRKG

GVKAAFDDFEEELRDEPILRVPQKHIDSSFEKDSSEEPFAADDDNSSRVTKISVSVIREECEEMKECENIEFPQVMDEQF

IAKSDAPRSYVGEYECKMLYTSAENVGKADDDDEDRPPSAAPSPPPSISPPPPPLPLRKPILLPTKSLEESPPVPTMKPP

MPPSKPPPPPRIPDRTPSMTRIAKPLVKTSSLRLAYNEQVKPSDVGKVNKLISRFETPQTKVTQRPRVIRRRLTRDESEE

YTDDEDDLEEDEEDGEQETDIVNANTERDATPTPTNTPRMYSIESDTESLPAVATNVNKTTKRTTIKIEENITTTQRSNA

SNGNPPKVYSDVPRITLNFDNNSNLNLSCASSEYGSPLEYPSSLIGSTETTPIPERRPQELVQNLKSAERSRRSMTRDDD

KFLSFDSDDENSYYSISSTGSSRYVVEI

**>Bdor_XP_011200418**

MDFKNLCQNIDVSSLRARIPSLPAMPQIKLPKSLPKLRSRRIFGRSREDLSRNKGSTKEQQRQQSIRQQSTQPPIPPADF

INHSPRHISTVSSLMHQQQQQQRGDSQRGTYRSACSVDDDYPSPRQFQAAGRISRPISPIHTSGNDGESEHVEPTVTKLS

LAEKLQKGYKDISEFRLSHIFAKKTVVRKDIILVDQYVERYNEEREREQAEQERRDRKIADNYRFNFKLSRQDTDNSKRS

VSSEESHKQVDNRHAKNMHEQSEDESGMEATPPPRKPGIASTRFARVRNPPLEQYITDESNNELDEDDEVPAPIAVKQKS

KIVQSSPESMSKEAVNRNALSKLRNMSQSSEETPEKLGERKRRRAPLSPQQSEEKQEVPAATVNENNPLSVIKQNIKRFS

KSIRRAQEPETSATNANTAEDTDGDGTPKKETKQISRTEKLAARLRKFASHEASTENLDEATPKKTEEQRSPIRTVITNK

LQNWKKSFKRRPTESEAQTGDETSPERNAEKGEKRTDNLMKKLRNIRQQKRAHSADDLDGTEEDEAHNTPTGTPKDANPR

KTVNFEKRFEQARQRTLKKMNEKMQQIKFFHKSQENLEKECSATTKEASKHDEVDAEEESEDERTVYVRPHTAARRAAAR

DTPSDSEDEQQQTGRERIKTDKLTQSEYDYDDEDMQHTRTLWTTKNLLNDSLDSETDVAFPRVLIHQDNSDQFESTLIIA

VTRPAPSKPSTPIIEEIHDDAPKFKPPRRSRSPSPGSGRAEWIPNNEIISNFLEITPPSQRRLSAEAILPAPRGHTRKMS

MDSNSDSDSWIPENARNKKISLPSKEDSMETATGVTLNSIDENEEPWKVHQHAYCEEKLYKAKSIDIFEASVRKGGAKAA

FDDFEEELRDEPILRVPQKHIDSSFEKDSSEEPFAADDDNSSRVTKISVSVIREECEEIKECENNKENIKLPQGSPKSDG

LKAHLDEHERKIVHNKDENSDKDEDEDDDRPPSAAPSPPPSVSPPPPPLPLRKPILLPSKSMEESPPLPTMKPPMPPTKP

PPRIPDRTPSMTRIAKPLVKTSSLRLAYNEQVKPSDVGKVNKLISRFETPQSNVSQRPRVIRRRLTRDESEEYTDDEDDL

EEDEDGEQETDTMNANSERDVTPTPTNTPRMYSLESDTDSLPTVATKVNTTKRTTVKIEENVTITQRGNGSNGNASKVYS

DLPRITLNFDNNSNLNLSCASSEYGSPLEYPSSLIGSTETTPIPERRPQEVQNLRSAERNRRSMTRDDDKFLSFDSDDEN

SYYSISSTGSSRYVVEI

**>Luc_XP_023296141**

MDLRNLCSKVDVDSIRAKIPPLPQIKLPKSMSKLKSRKIFRSSREDVNLDSRRSSKRLPANSENIPPLGFINRTPTRIST

ISSLMNQTGGDSVDSKKWKSHNYDDDENSYRSAGGIDDYPSPLSQRFEMRSSRPISPIKEPRFSHQAHGQSEQNIKMSFG

EKLQKGYKDVTEFKLKHLFAKKTVVHTDKIEITDYIQSFDEDRRKCERVQQKRDNDIADNYTFQFKESKQKSEESYIDDD

SGPSSPQQTIMPKKNSKNLSELSGDESGMESSPPPRKPGIASTRFSKVRHTPLNMSLEDDYDNELDVEEVDAPKIDSSAI

SLRDGKHLKSPEKSNNSYQNLRRMTTKATRDRPPPPPSSAPLNISMEEYLDDDEDEGDDHNDDDYGTDDNYDLNVVEVGT

PKKIQPNTSSLYDIKTQKPTEESVDSQQNPRKFKSIKERMSRLSKQKSEGDDTAISEKKRKAPLSPQPSEENEADSSAAA

RKSDKPFDAIKQNFKRIQKSVRLPQQMSTRSGAESENEEATSDNKTKISKSQQFTERLKRFRSIDNVNKNSEDNENSESP

IHKFTNKLQDWRKSFRKKGDSHNTYNDDMDVTTSPQKQERNVGSLMKRLKHIRARKRDSIEDPDDDEEGGDTYKESLAAR

AKFQLEKGVVVASQVPQITMKKISETKKYFKKKQDNEVKNRTEKPQSEEEELEDGQEKTISKPSRPPPPIPRPYFRNSSE

EDDDDVDNNKFITKSIAESQLIAVPAATAVWATKPMITDDEDDYDEELPRVLLHQDNSDIFESTLIIAVTRPISRTVSPI

ITELSPDSAEESTPDTKPDDWIPNQDMVATFLEMTPPSHRRLSKDSIHSRGHSRKHSIDSSSEDSWIKDIPKGPSTAYLN

SVDENEEPWKIQKTNNEDNLYKTKSIDLFEMQKKHGGVLTAFEDFDDELKNTPVVRIEKESSSVCENTPAEGKIINIAAV

ENENVPDVKIQMKTVNVEKVENENTPIIKIETESSNVVKVENENISSIVDLEDEKSKDLEIPRNVDYYERESSEELEIPD

DDNSSRVTKISSHRREPRTEELQNSDNDNSSVVSKEIVNSTEPKRVELQNTENGNSSDVKNISKVHKEKIQNTSESNKTV

LSEVPKCKDLGNHHIENSSRLQENKTPKAETKLKLQHFGNSQESLTGHLDENKLFDSGEEEDNEESDDDHQFKDVDSPPS

KSPSPPPTISTTPPPLPQRKPPISLLTSQPSIDLTSLPPPILQPTKPPVPPQRINPTAPPPKIPDRIPSTTRVSRPLVKT

ASLRLAYSEQVNPQDIGKVNKLISRFEPEGNKQRPKIIPRKPLITYESDDYSDEEDLIQLLPLKPKDKFRDTTPTNPTRI

QKSFSIDSTDIPNNNVKPIPEPEIHTSLSRKAISQISLDNSEIISPIPSISISSSCLDVNSNFSQPSSVYGSPLAFPSSL

VGSTEVTPLTNRKDCEHLAMRRSRRSLARDDDHFYSFDSDEENSYYSISSTGSNRYVVDL

>**Scal_XP_013105203**

MDFRSICSKIDVDSIRAKIPPLPDMVTQIKLPKSMSKLKSRKVFRSSRDDLNTSPHAKRSSSRRSESSDIAGQAFAIPLA

PPSDFVDHTPRKISTISSLVGQSPVRGQTNRGGGGVGGRSYRPTTLKGPHFDDDDDEFSYRSAGGIEDYCPSPLNGPRKK

STISRTSLDMSPSHLQATDGPPAKKSLMQRAKQGYKEASEFRLKHIFVKKTVVRQDTIQVDNYVHRYEEDRQRQQMFEAR

RNSGISDDYEFEFKLSSRKSEESFGDGSPEGSSSHQWKSHNKKTLSEQSGDESGMEQSPPKRKPGIAATRFSRVRKDPLH

MSLEEDDGIQQHLKAAKRPQHVDDDDDAEMQEIPLHPIESALKKKLSQKQEHLSPPQKMTKSKSIKKGLGELKNESQEES

LSLDEKEDGDDEEEDEDFQAAEQELEMETVIAKRPKTRPLPPRPPTQKSQSIKNPPLANLRNVSQEESLSMDDDNVDGSP

EEQTKTASRSSKILSPIQSSLYRLKGTLSKNNSADDSLSSGSTKKRKAPLRGQKSQESLKSPGGSEKASSEKASSKGNFK

ENLKRFHKSIKLPSRSSVSAAESDVENQSDKVKQSKSQQFAERLKRFRSVDNVDKHTEEGQEERQTPGSPLQKLSSKLQD

WKKSFKIKASEKASHTEDDDEMSGATSPKRKKGTLIKRLQQIRMRKQHSQDDPDDDEEGGATYRESFRSRAKLQWEKGVV

AATQMPQMTMEKLKESKKYFRKRQEKEKEKDKEDDKNKKEKPEIGVANNASGESDLEDSEEETAAAARRTKKPAAKALAK

AQDNSRHYYSSDDEEPEEEEDLTKVTLEPYQPNVLVPLSRAAWSAKPIHPDSTGDEDDDMLPRVLIHQDNSDVFESTLII

AVTRPIPSRSSTSSPYITELPPDYQESPKGSPTDSPKPSTPETKADNWIPNREIISAFNAASSSSRKCRAIKRDLSKDSL

DSTSPQRAYSRNQSMDSSSDESSWLRGVNREPSTAIGLIGNRHIDLEEEEGPWKVGAAADNTKDSLYKTKSIDIFELHKQ

QGQALHIFEDFDDELKNTPVVKIEREAKEQQTNNNNSAEDEEQEDDSSDDEYDNAADDDNSSRITKILRSEENDNKIKEA

EASVTPPLAPKSSLDNLVLHISEDEDLADLPTIAIDLAENLLENCRGIAASREVNNKQPAKVETKAEAKEMGEIPKHNQD

PNGNKPSGNTFQITKPLVQPLLATNQVPLVEEAVANLQQRLGEIPRDQLTIECNLATQLHGSSRECLLQEEIKPLPTIIA

AAKPKLANFQYANSQESLMGNVDPNETPDDEEDSSYKEDDQDSDEIEDMDRPPSSAPSPPPVQSPPPPPLPQRQTSPKVY

KAQQQAAPPPPTAPQKERTPSPPPPLPQTKPPLPPNRPIAGPPKIPERTPSVSKVAKPLVKTASLRLAYSEQVKPQDVGK

VNKLISRFEPQGRPRIIPKRNYMTQESEEIFSDDEEETIKRLEKLEIKDVNKNIINENEPLLQPLNIIPDEDVMPVRQTF

SRDRTPTNANISLETTIATKTEETSFNSNNNNLQTSRKSSKKLNRAPPPLITTSQPQPEPSSSPTNLLLTPTPGSTTNSF

NQFDRNSNYSQSNSEYGSPMDFPSSLMGSTEVTPISMRKETEFLNTHRSRRSMTRDDAERYYSFDSDEENSYYSISSTGS

SRYVVEL

>**Mdom_XP_005188505**

MDFRNLCSKIDVDAIRAKFPPLPEVMTQIKLPKSMSKLKSRKIFRMSREDVSRSSSKRRSSSIDDIAKQQQNPLGPPFSI

PIPPTPSDFVNHTPERISTISSLVDRSGGGKGRSLGRGATMKKRHAFEDQDEENFYRSAGGIDDYCPSPEHMPATQRSYS

TRPMSPPRKTTGLSPTRRTASEQNVKMSFKEKCQKGYKDASEFRLKHVFAKKTVVRQDTIQVTEYVRRFEEERRQEESEE

EREARELEENYDFNFRTSRRKSDESFGVSTPRDSPQRQQQPHLARLPTSNPKKTLSEQSGDESGMELTPPPRRPGIAATR

FSRVRKDPLRMSLDEDDGIKSPPKNQKSEEEEEEEEGDDNDIELDEVEVDKPPMPLHPIESALYNKINKQREEEEAEHLR

KKEKLQKSRSIKKRLGTFKSVSQEGSLASNSPLKKRRAPLSPQESEEKSKSPKAEGGGLIKQNFKRLQKSIKLPNMSSSR

GAESEGEADAESGKSKPSKTQQFAERLKRFRSVDNVDKQTDNEDGGGGEGTKTPGSPLQKLTHKLQDWKKSFKRKPSENT

DDEMATNSPQKKEKPVGTLIRRLQHIRNRKQASTDDPDDDEEGGHSYRESFKSRAKLQLEKGMAAAQHMPQLTMEKLKES

KKLFRKKQEKDKDKDKDKDKKKDKAGAAAAMKSNESGVSDLEESDEEEENNDGKMRMARKKKSKDNKEKATTRHYYSSDD

EEEDDEAGAAGGGEDEKSTKITLEPYQQNVMVPLSRAAWTAKPILTDSGDDLDEEDDVLPRVLIHQDNTEGFESTLIIAV

KCPISRSPTRSPAYIEELPPDYEDSPKASSPMDSPKPSTPETRADSWVPYKEKVANLAEFQTPLPRRGERRASKDSMLSS

GSRSHHSRKQSIDSSSEDSWMKDVPRDTSNVIGLDRVYESEEPWKIHNGPKMDEKLYKTKSIDIFEKHKQQGGGMALHIF

EDFDDELKNTPVVKISKEEEDREWDEGKQEGGSKNQNEPDESGEEMDQPDDDNSSRITKILRQPEVPKTEKEEEAVKKKE

EDLEKSKFGNKLYGNSRESLNGNMERNAPQTDSGAVEDNEDSSEEDDDNDDDDPEEEESDEDRPPSHKPSPPPQEPTTPP

PPVPQRQPSIRLSCNIPRQPPPPLPETGKSEEEEEDEEGVTSPPPPLPQTKPPLPPNRPRVAAPPKIPERTPSMNRVSRP

LVKTASLRLAYSEQVNPEDVGKVNKLISKFEPQRRPRIIARRGYSSQESSQDYSDDEEGEEGETESEVEKPRSRNMSLDR

TPTNKPKLQKSFSIETPDSEQEMKVNNKKEESSSSRMQRRAPEPPTAGKRNEENSSNNNNNNLRDLEKLQDLHITETKHP

STNNNNNNPEIVIQRGPPPLPPLPIIITTSQASPVKNSTTKRNNSTLSQYDTNSNCSQPNSEYGSPMAYPSSLMGSSTEV

TPTSNRKETEFLSTTQQQYRSQRRSMARDDEHFYSFDSDEENSYYSISSTGSNRYVVEL

**>Ebal_s8567_L_28372_0_a_29_2_l_3010**

MKEGMSQEFDSTWNLQGIRNRFTKDDTVTEQKNKRNLQSQESLDEHSDLEKKSGKLSKVQLRRGESTDKNSEPAISSPEGKFSGIKMSINRLKSRKPQRQTSEESEIDLDVQESPRKNMSDQLKAGLRRLKKTDRTLTLDEEESKTPKESTTSRLQNWKKSIKLKRPSEVATEDNDGEAPPANPSPLGRSHTLMRKLKQIRSRTVVSEDDADSADPSQGNDQPKQKSSNLEKLDQARAKALQKVNAQVQKMKNFHGKSKDLSTEESPDKPNRKTKPEASASRPLPTVPSSHSLTSQLSLDDNDEIPRLYPQIAPRRINKPETKPSKPGILASSNSMESTISLDEEPPVLQLSKPNKSGSLTKDNISKPIILGHSHSMDDEDDDQDDEEDGVPRVFLHQDNSDSYESTLTVAVTRRAPPVPTALITPKAKYLAPKTEWIPNKNAISSFLEMTPSPESSTQQLDTGSRGHLRKRSMDSNDSDSWIPDNFRNKSCSSSTIAGLNKISENTEPWKVHSVFSEEKLYKARSIDIFANRDGGAFKDFDDEMKNVPVRRIGAESAASLNKSYGGESSDDDDYDNASKVTRVKYESPMVSTKAAIESMRSGNSYESTESNQASRGSPVPTNKKTTAQIGNSQESLDANYDENDEISSEDEEDEEDEDDDEAPTMAPSPPPSFSPPPPPLPLRKPPRLVPERNDSMVRLAHPLVKQGSLRRHFSEEIEPADLGKVNKLITRFEKNPTYENVPRIQLIPSDDDDGSVVQVEEPKVTKTKENDDKKRSVQYSPTISSDDNLLKREKKHAEQVKDNGAVKKHQSRNGTSDEEGSIDQKKVARNSSKRRPAPIPNQPEKLDDSRNNNEDIGDKSQNKNTNEKNDNNKNNSDKDDINDIRTKQEHRRPSPSPEYQENLDNVSIQSPNSIYGSPIGSICLSERISTSSVYGSPIGVSYPVRTSDHQMRSNRRLSNRSSLRDDDTFYSFESDEENNFYSLGDSSDMRYVIEI

**>Mequ_C101934_a_40_0_l_3795**

MDLKSFYKKIDVDNIKSKLPPMPQIKIPKIKSRKIFKSSREDLSQQQSTAAGAFAGPPMIPPPLPPPSAFIDRTPTRISTVSSLMNIDEGYVCKNQYAMSTYRTASTFDVENYPDTTENGDMTTSGSATSKFSDKFKKTYKDISEFKIKDFFGKKTVVKKTEIEVDIYARQYERERREEEELDEDEADSTYEFQIKEARRNLSKSSDSISPPDSLASGVPSPIRSELSHLQRNITPPLLARRRNAIDEPGPSRLHLEKSMSDDSTHGPPRKPNRSSLWGIAEKDSIEEAQTSQLKSYKAGISKLSGFKMSRKETPTSSDQPTTSDSVWKLKSLRARFASEDSGSEHKPKKRMQSNDSIDEPSETEKSEKKEWKMSNLSLKQSAYDNRNSESHTSSPEGKFSTIKQSIKRLKQRKARPHASDDSEPEQNDEVAKPNMSDQLKAGLRRLRLKERSFTFDDGETKDSQTPTQTTSQTTTQRLRNWKKSFKVNRPTDSVNEEDETTTRPPPPPPGRSATLLRKLHHIRSRKAESDNGTDDESGDQKLSNDNQQRPKSSNMEKFEHARDKAMKKVNEQVQKMKKLHSSKSRDSPIESSPEKPPRSRRIEIEDQSEGVIAELPRSAPTVWATSRAISQSFESDNDFELPRVLIHQDNSDQYESTLIIAVTRPAPAIPTAIINEIIPKNKHKLDSDWIPNKSGISSFLEEIPSPSPTSKQTEADRGHGRKRSMDSNDSDSWIPENYRNKNSSSSSTNALNKISETSEPWRIYGANKDEKLYKTRSIDIFSNRDEGAFADFEDEMKNVPVRRIAAVSVESVAHSTDDDDDYDNASRITWLKRSSPRLNINLSLDATECDSMKGSMEGNPEVDLPPKKGQIGNSQESLDANYDENETYSSDDNDDDIPPAMTPSPPPSYSPPPPPLPLRKPPRHIVDRNESMVRLAKPLVKQASLRRHYSEEIEPSDLGKVNKLISRFETNPSEEDIEATETNSTESARDKQEVEEKKPTKPKEDTHRNGSKKRAAPLPRKSEEIPTIDEITPTHQNNNYNNITSISDDNDNIVAPKDMSTLETLDNISIQSPNSEYGSPIGSICLSERTISEYGSPIGVAFPTKTIARRLSNRSSLRDDETYYSFESDEENNFYSLGDSSSTKYTIEI

**>Eper_s7865_L_21645_0_a_25_9_l_3667**

VMDLKSFYKKIDVDNIKSRLPPLPQIKIPKIKSRKIFKSSREDISQQQPPGGMMAGATPLTPQLLPPPSAFIDRTPTRISTVSSLMNMADGMRDKHENEWSTYRTASTFEAEHCPDDGVDKVSGSAASRFGDKCKKTYKDLTEFKIKNLFAKKTVVKKTEIEVDIYARQYERELQEQLELEQRQNDDRDYEFEFKEARRRMSKSSGSESPPGSDQSGVPTPLRSQMEYLQRGATPPPVGRRRNAIHDGAQTKLRLHRSMSEESSNEPPMKPNRSSLWGINEQDSMDDVPESQVISHRAGISKLGGFRKSRKEKAPSEASAHSDSVWSLKSIRTRFTTEASVNERMSRKDLQAQDSLEDQSEPEKKGAVPRRKPLERKESLDKNSDTHASSPEGKFSNIKQSIKRLKQRKRSETEESETEQGNQSTKSNMSEQLRAGLRRLKMKERSISFDEEESKPPQATATTRLRDWKKSLKIKRPTEPMPDEEVKSPPTRSLTLMRKLNYIRSRKSDSDEGEQQTEAAANDEQRKPKLNNLEKLEQARDKALRKVNAQVQRMKKFQGKSRDSPTESSPEKQERRIQSPEKRPGRLNKFGSDEQSEGEVAEYSRTTAAVWTTSKIMSQSLESESDIEMPRVLIHQDNTDQYESTLIIAVTRPAPPAPTTIIKEIPPRTKQKSDWIPNKNAISSFLEMTPSPPPIKHLDISRGHFRKRSMDSNDSDSWIPDNYRNKSCTSSTSAGLNKITENSEPWKVHSVFSEEKLYKTRSIDIFSKHENTAFEDFDDEMKNVPIKRIAATSADSIDKSCTESTDEDDDYDNASRITRVKRPSPLTSTTAAIETQHSMDSNKPCRGSPFTTRKGQIGNSRESLDASFDDVSTDDDDAPPSVAPSPPPSFSPPPPPLPLRKPPKMIPERNESMVRLAKPLVKQASLRRHFSEDIEPNDLGKVNKLISRFEKNPSEEHLPTIVADDEPTIATQVEEKKTVKPKEDGNRSGSKKRPAPIPKQPEIQKADTLNNNTNNNNNTKTNDSGMKEDKSSLQLPVSPETPRRKNLDNASIQSPRSEYGSPMGSMYFSESVSISSEYGSPTATPFPPKNMDQLHSCRRLSNRSSLRDDDTFYSFESDEENNFYSFGESSSSARHTIE

**>Hfus_scaffold93651**

MVLFSYFFCLFLDKLDKLPPVPLPKIKTRKIFSTKGPSECDPPPLPKVPPSTVVDRIPCQISTISSLVEGAPKYDYSFGGAEVPAEFIERHAKEDNFKKDNGGNETEKRSKGFSKLTKTYKELSELKFKNIFGKKTIVRKTEIQVDMYRQRYEEELEECSSEKSEYEFDVHSLPTGNERRISVATSSQSQESNQQQYLQQKAESFHSIDEISPNDSALYKIKRRAESTDAIASGTSTNITPQNSEENSNKPPHLRALDKARNKARKAKKAVNIKIQKLLHLKDKNIEEYSENPEENNPHLVRFKRKAAIKKRSSLPRNDSIESGRDSLQNSRESSVDYHPKKDWTSQRMLSTESKETPHCLKSQFSFESSDYERENIAVRPAPPIPSIPKPPKIESVSNRKIVIEFNKISPPLEQKNIGTDFLKGHSRKPSMDSIDSDSWIPEGFRNSKQQVVGLNKIEENMEQAWRVRSLDNMKKLKKTQSIDIFTGEESENAFEDFDDILKNVPVISIKNKIPSKDSIVSMDRPKRSTAPLVKQSSLSRQFSDEIEAENLNINVNKLISKFEPNDISKGERIKVIATVEKSRSQSIESSPEKTAKLVPQERLLRPWENSTSMEDLNIKSVKSETGPVRPITAKEWAQKVIAARSLGSTNQGSDDVFYSFESCNAGKQIKVKISDRIIFFFLTSKTMSF

**>Hill_scaffold202986**

MPKIKSRKIFSTKTPAQPEVVEPPPGPKPYVVDPSPVRISTISSLVHDAPDPDYSERPQFGRLTLAHSAVDLTEHVATKFDDSKDDVAAKNLTLSEKLTKQYKEFSDFKFKHILGKKTVVRTTSIDINNFSFRPDDSSPEKSEYDFEIHSLRAQKLDSGELDMEDDESIQSSGNRRRHCQRQQSAESHKSFQSNKSSEKSAPLASANEEGPINVQPNKSSLFKQVKTMSMKKIADFSERHERSEKPEKVEKPEKAEKSHEDSAEPSKRPSLSKIPFNRQSIEDACYRAKNTVSNKIQKLKSLQSRGRNPDASVQKQDTYTKKSEPDIILPNISYGPNDAGYIRSLSPQGDSSRPSSRSWRHESAKRCSDLSIGHSRFLQHQDSIESFDEEVPSPKHVQVLRHNIGGRTGEGASISDEFSEEEGYGYLRKDTPIQSEPLQAPVRRFSQNSWESVKMKKSVKLRSDNXRSDNAQQNIKIARPAPPAPVPKTTGHIGFPLQQPSKMEMIRKRFSQKQDDPIPTNNNFDMNVSNDSLDSDAWIPDAFKGPTYNQISFGVIKETEGAWSTAPRKRPILQKSRSIDIFNANGEEAFDDFDEALKDVPPVVIPSRGGHKHSQRAKQQPIIKQNALLKEEDKILTGGTERRSQNFNKLL

**>Mabd_JXPG01001308.1_scaffold1565**

DIFIILAKFPEIKIPTFKSRKIFKASKEELSKKNNSYTVSSAEQQDLPRYPPATFIERTPRRISTVSSLQTQEHVDDILVDSRVNGSYTNYEHRDFDEKKSRPVDEGLHQRFTRHYREFQEFKFKNIFGKRTVVRKTEISMDMYADRYKEDLKVERQRRPQGRQDSIEYNSHFQNNNQYSNEMFSSPTVSIKSNISTPIRNQIKEEFQKPIRVQMKECKFQSEPIESEEEKKPKVEYSQSWRFGTLRGRSLNRKGSLNKNKQSIRQKSIKKQNIVKKTNEKFRWTLKSNNKSKAEPSEVTVEAGIKKKWTFKKPKEEVENNQEDLSKLDEHRKNRWTLRKPKPVTSNDDEASNPPPPKKKWTLRSNTSIDDEPENRQTDSSKPLKKIKKKHKTRPKEFEIEDVPPSKEVRDATTKRRSWSVKKHFSSSIESYSSAKDDIRNSSWNFKKIKGSEVLSRWKTRTMQVLSQPNLSTVGLEGSSPEKKQKITLRIWKRNKHIKKSSSSEDVQHPKADSKSFFNIKYFNKKDHEEPVSVEEVIIDNKKLTHKERYENAKDRAKQVVNDQIQRLHLFRGQQASTNSEDSAKAQKRKTGFDEDLDDVVAAANIEEIQNSPAAIWASEQEFSQTINSLETNDFNMNKPKVLIRQQSYENREPTLVITMTTSRLHPHISPLFRGRKLNSLDSNDSDSWITNTYNSSIAEKELVENSAHFVTKDQTVNKEEIKKRSFKKEEVRKTSVDVFENKEPFENFENELQDTPVINIDSEIPPPPPSTSPPREESPEKEVFETIDIRSNLKVIKKNEKPLKKISKDSIVARSRSSFRRPAAQPPSIPERTASMVNLTRPIIKQGSLRRKLSADLESKNMGIGKVNSLINKFENSYEDLTDSTNGLIFSTLGQTININENNIHKSDDSIKMNEQTNEDPIVKLKTIDVRRKTGVSAVPIPSPRLTLEELPSPLNTDTVVDIPVILPVGNTATTTNEDNIDNNTKKNNNINNNNSKCCTLEEITEANNNLKQATQSSMIHSKEDENYFSLSDEGEFEIS

**>Pmac_scaffold42832**

AKMPPLPQIKLPKSMSKLKSRKIFRSSREDVNSVSRRGTLKRIPANSENISLPLGPPYTIPPSGFINHTPTRISTISSLMNQRGGDGAGSKKWKSQQNDDDDDNEHFYRNAGGIDDYYPSPLFQERLSRPISPVRAPPIAQEIQAQSEQNIKMSFSEKLQKSYKDVTGLKFKHLFAKKTVVHTDKIEIKEYIHRFDEDRRRDERIQEKLDHDIAENYRFQFKISKQKSEESFVDDSPASSPPQHNVEPKKNRKNLSELSGDESGMEASPPTRKPGIASTRFARVRHAPLNMSLEEDNDYDDSPKINFDLNAFDDTTQKQKSPKKFIDSWQSSQKLIKGIPSTSCSRKQHTSFNMSLEEDDDTDNNIELNVEDVDSPKMKPNTSSLYDIKKHKSHEKLSNSQQNNRKSKSIRERMSRLAQHKSEGDENKVTSDKKRRAPLSPQQSEENAADSSAAPTKSDKPFDAIKQNFKRIQKSIRLPPHMTTQSGAESENDEAYSENRTKLSKTQQFADRLKRFRSTDNVDQSNDNHEVIESPIHKIAHKLQEWKKSIKKKVDAHSNGNEDVDMSHSPQKQPAGSLMQKLKHIRSRKHESTDDPDDDEEGGETYKESLTARAKLQLEKGVVAASQMPQITMKKISETKKYFKKKQENEVKNKTPNPQSDEEDVDEEDNQEKTTCKPVAKSIPSRPPPPIPRPFFGNSSEEEDDIDNQRFITKSVAESKLMAVPAATAIWTTKPILLDDTTDYDEELPRVLLHQDNSDVFESTLIIAVTRPISRATTPIITELPPDSVEESTPDIKQDKWIPNQDMVATILEMTPPLQRKLSKDSNATTSSRGHSRKHSIDSSSDDSWIKDIPRGPSTAVLKSVHENEEPWKIQKENTDEKLYKTRSIDLFEMQKKHGGVLSVFEDFDDELKNTPVVKIGNDKNSAIDKQEKEKLQDLKKRRNVDNYDYGSSEELENPDDDNSSRVTKIFVHSQGPKNIEKAKVETELKHQSYGNSRESLTGNLDDKDAIDSGEEDDDEDSDDDGDDYHKYGDEECEVNQPPSKTPSPPPLPQRQPSFRISLLNQSSLDQACPSVPPPPLPLTKPPIPPNRPTPPPPKIPDRNSSTTRISKPLVKTSSLRLAYSEQVNPKDIGKVNKLISRFEPEGPKQRPKVIPRKPFITNESEEYSDEGDLVDLLLTKQTEQIKDTTPTNHPRMQKSFSIDSDGFPNNNDTTTPNQKIHTSLLQNVTSKNTVHKTGSATPIPSISISPSSLDVNSNFSQPSSVYGSPLPYPSSLVGSTEVTPLTNRKDSEHLGMRRSRRSMARDDDHFYSFDSDEG

>**Chia_scaffold342677**

CKPMDMANTVETKTINEEKNPRNVINISPLKRKQENSSSRFSKVVHPDQIMPLEFEKSIAHDFIKKEIVSHDTIKSVVQKHPNDGSTRCGKMQKFKKKLKQLHARDNICHIYNSSNVDIKTIINEKIQIWKNSLKRTNVTNCEYDIKNVNNFVNREKHLPALKKSKKHIIKQGVGDNADSPMQKHNRSETLMRKLKYIRARKSSNSIEDNEEDGVSVETSTCHCDGTPKLTVEKLTNAIDPLAIVQNEITNSDPQISNKSDLGTYNRSPLSTVSLSSSEVNHYNHERNANMEDGYGCMNEYAARNCTKWTNNSILNESIESYDKNCDYPQVLIHQDNSDFFESTLVISVTHPATSTPSPNVKKKCFQNAKENNKPQQNFRCASNELITDETELPELSHRRINFENKLENFSVSKILTSANLKMRTCSAADINESNGNTNERRQLLKCRSIDIFHANNMSISTAFDNFDEELCNEPILKISPIIDITRDNDNNNEESSSKKLKQFCCSNENITLTNEFGKSNIEKIIQFGHNEDNLSTWVNECLDRGSIDNDHVAEDQCNRSPPEILSTSQIQISALLVPSPKQNANRITSIQKGTDCIGKKKGSMSLPAIRPTLGQYKMTRCTSFHKPAESQKIRFENRCVKPLVRTSSLNVPFEQFKVTDIGKVNQLISHFEQPVERTQSLHKQNKKSSILFASKTTTTETKNNDYNKLESYLNLQINNVTNNNKIREEKNYRYNNARNWDANSNYNHSDSEYNNSFAVSSKLYFSPNVTPTNIKPNRLRVEEINNNRKQRGFMKDPDNYYSFDSDEGRFYKTFVPRLHVIIKI-INMYFTENSYYSLSSSNSSRNVVEI

>**Egra_scaffold45734**

IDDDYAMCTERSNRDTIVSTDSIHHHSSTPAIIRTPTEDSFTEKLHRRYIDFSDFKLKHILSKKTVVRKDNIEVDQYVKNFKREIAIEQEEIKKRDIEIGDDYCINISLSPQKLSDSDCTSNTIETKSLNEDNCQENFDHFSCLKHKPGSALRRFSKGLNRVQLMSLQYEENTVQNCIEEQLQSHDTIKAVVQKPPNHSNEKISSTKLQKFKTKLKRLHSRENIFSDLYNTRNVDITSTMNHKIRIWKKSLTKNDVNKFENNINIVNKKLNEEKGVPSHITVLCLLKKWKKHTINQGTRENVDGSMQTQKRSETLIRKLKYIRSRKNSNSVEDITLEKGTNAIQQLTIVQNEDIINSVQQSPRKPDISLSPLKIERHTPERHSKEDEYGCMDEYTARKCSESLFSTLNLSPSKIESYKPDTDDNMDDEYDCLNVYTADNCTNSNKKAISKESIVTDDKNFEYPQVHFHQDNSNFFESTLIITVTEAASASPTPKLGKKSIKHAEDSKIQNLKWASNKNTIAESKSSELTNRMINFENIETYPGDNIPPLANLKKHTYSTGDIDGQTNGDAKKRQIFKSRSIDILDVNNLSTSKAFDNFDEELCNEPILKISPIRFLSRDNEKNNEEQLKRNYLPNEHMTVINEISKAKMKKCYQFRDSKEHFITRANESLDDDVEFNWPSPKTPSSPLLLISPLLQPSSEQRINNNSSIRNGSEFIEEKKGSLSLSRISTELVQWKPIQCNSLHNELSKTTELLSTKFEKSYSKPLVRTSSLSSAFQQVEVTDIGKVNKLISHFEQPVKRALSLNKTNYDNSNTSSILFSSKTPPEKIKNNINKQSERYLNVEMNNVTKNNIFRGEEKKNKCYNSYTCHANSNLTYPDSEYNCSSANSSQVYYSPKVTPTNVKPFRLAVSDTNNNKKQRAIIKESDNYYSFDSDEGGFKIMFIAFSTNIKIYFTENSYYSLSSSNSSRYVVEI

>Pcoq_MNCL01000070

LDKLPQVQLPQVQLPKIKSRKIFSTKGPGENVPPPLPKVPPPTIVDRWPVTISTISQLVDGAPTYDYSFGGAEVREEIVERHQREDEPAKEEGRPAKANLRFSEKLTKHYKELSEFKFKNILGKKTVVKTTVIQVDEYKRRYEEELKDRTSDHSDYDFELDSTPATSANPSRRCSMRTPLPAKKSFSEIQLKPESLDNVGELEEVPPNDSSLYRIKERAKLSASSIVMVQESLKISKETVKEAARGTLVSEGSTKKSHRQMLDKACGRAKKAVNAKLQKLKQLASKNSQDQSQDQKVTQPKLRRKPEIKRKNTKKETPSIRKESVSDSCESALSEQESLDSKNVSLDLEPPRPEWVAKRILSNESETPRCLKSQYSLDSYDSEREVIATRSAPPPPPPKRLQEQQQEQEPPLDEWGPPPPLSSQTNVIFHKISSPVLAKGAFAELPKGHLRKPSMDSIDSDSWIPETYRNTPRPQIGLNKIEENDPEPWSTQAAVIPQAPGVCRRQKLSKKKSIDIFASEEGEKAFEDFDDVLKNVPVIPIGTENNDVELPLQAEPPLPAKPLVKQTSLTRQFSDDIEAENLDAIDVNKLITHFEPLQTDSTFTEVKQIQVMAIMEQGKLQHPNSLPXXXTARTRRISAKEWAVRAKVTRSLDSGAQGSDEIFYSFDKEQHGK

>Hing_s11934_L_39310_0_a_35_0_l_2480

TTVIQDNQSEIPIIDNTSKDSVFIKTMSTTPTRRPIATPPPAPLRYKKESSIPPAILPRKSKLIKQNTSLEVEDGSEASICDLYEKIQDFANFTNNKKEAIPNSDAHLTSLATLKAIQAKQQYEAKLKAEKIKTSNSLLESSKMKIPNKNENIVVGNSSTAKILQYQQQHQLNQKHLEQQQRSEENIQKEQIPTQQQQQLQQSLEKPQPTTTIRTKTSTTTKLIENDINSNPIIKKTEMSKTNSNIDNNNEPQSIIMTTATGTTATKTTTIAPINTNSTNIQPDDKEIATTVIDDEGNYYSTTRIKLADIKKDETFYSLDDEEYQQYSLSGPTIVNELYTIEIXPKESPPPLPPCSPPPLLDMTKIYDDIQLEFEDAVELMPDFDTPHKFNFKPDTLQVILESPSSATNSLDIVELSIESPSTPAFFTPIQSPPVPKRTTPSPKPITPVESSIPEVLISTITTLNTLPIESTIPKTSSPVTETHLQTEKVESNKSAIPRPRHSRKPSMDKPQIQASTFAKDILPRSPIVGLNKIGEITEGVGQNGSEIVKKWSSVDIFNSNDEAFNDFDEALRNVPVIPIVIENTDDTLLNDDNNNVNNNNNNSHSNIRECKSMSPSLEKSSELLMREHINYYSDDDDDDDDDDDDESLNIENRMPTGVPVVPEKPTRPVVKQASLRRQFSDEKDAEDLNKNIDVNKLRSRFEGSDLPPRPPERMSFRKYEKVQVYYTGYL

>Pant_s4671_L_6202_0_a_7_7_l_2037

DRLKSLRGSRKTMTVDEAIRDNKKLSHLERFENARDKAKLRVTNQMQKMNFFQDKTPKARDSLDSQNADTAGTANGFSFVRNDPVDECDGDIEEIPRRSPVAIWASEQQFSQSFDSDDFPHIPPVMIHQQSNGDNEPTLFVSMVGRRAPPRPFPPAELSDDSSPQSQNTVKCLKTPELNPNETFMSPTPQRANSQSPTEAEENFSSVQIPRPKLAPRPSENAWIPNKQVISDFLSSSPPSQLKSGRGHSRKPSMDSNDSDSWIADTIKRASPLTLKKIDEKTEPWRSRPPISPLKEYGPPPPVVNLATNNASVDIFTGEGKTPFEDFDDEMCDVPVLIVTEDKEEAIKVVTKVQEKQEIAHILETQKAASVEKELPQMIPMQISSSIDEEEESSDDDEDLPPSLAPSPPPSFSPPPPPRPPPIPIRKPTGPPKFPERTDSMRNLPKPIIKQGSLRRQFSENLASDSFNFGSVNKLISRFEKQPSPSSESDNSLDNSQYDSPNENTEKIQVVAVIEPKATTKTNPIPIPPKSAAVLLCDKSKTTVQQEPALSLSSRFKQRMKNNNPTPADENKNQRLPSPPPPPQASPPPPPPPTNPPPQMATKKDLLSVQTTTREEDTFYSFDSDEETDNKFYSLGETGVVTSRRSYVIEF

>Lbif_C81360_a_6_0_l_437

SYQKFGDTNDWIPNKQIISSFLRNSPPVTSKLFMEIPTRGHARKQSIDSNDSDSWIPDNYRNIKLSPIQSLNKIEEKYEPWKPQSLALEKKITKDESLDIFSNQQSGAFEDFEDAMKNVPVMEIVDLENIPPKTLPPAPPLPPPP

>Ddia_QYTT01089660

LPKIKSRKIFSAKNSSENEPPPLPKFPPPTIVDRSPVRISTISALVEGAPEYDYSFGGAEIPKEFKERHLKEDEIKKETESASKRTKGFSKKLTRHYKELTEFKFKNILGKKTIVRSTEIQVDMYKQRYEEEMDEHSSEQSDYDDFDVHSIXXXSSDGENEIMATRPAPPVPKILKPPNSPVTSSFNKAKKPNNVPEFPKGHTRKPSMDSIDSDSWIPETYRSNRAQIGLIKIDENVEPWMTKPIENVKKLTKAQ

SIDIFSGEECEKVFEDFDDALKNVPVIAIPTNNPPTETPLRTTFKRRERPLVKQTSLTKQFSDEIEAENLNVNVNRLI

>Cpat_C30667165

IVKDVLPRSPIVGLNKIKGKNEETSRNGSEIVKKWSSVDIFNNNDEVFNDFDEALKNVPVIPIVIEKNQALLNNDTNSKNNGENSTTNNNIREYKSMSPSLEKSFDLLMQEQENDDSDDDNDFEDIKLLNMEYKTATGVPLISERSKPTRPVVKQASLRRQFSDEKDAEDLNKNIDVNKLRSKFEGSNLPPRPPERMSFRKYEKVQVYYTGYL
